# Newborn blood DNA epigenetic variations and signaling pathway genes associated with Tetralogy of Fallot (TOF)

**DOI:** 10.1371/journal.pone.0203893

**Published:** 2018-09-13

**Authors:** Uppala Radhakrishna, Sangeetha Vishweswaraiah, Avinash M. Veerappa, Rita Zafra, Samet Albayrak, Prajna H. Sitharam, Nazia M. Saiyed, Nitish K. Mishra, Chittibabu Guda, Ray Bahado-Singh

**Affiliations:** 1 Department of Obstetrics and Gynecology, Oakland University William Beaumont School of Medicine, Royal Oak, Michigan, United States of America; 2 Department of Studies in Genetics and Genomics, Laboratory of Genomic Sciences, University of Mysore, Mysore, Karnataka, India; 3 Department of Obstetrics and Gynecology, Icahn School of Medicine at Mount Sinai, New York, New York, United States of America; 4 Department of Obstetrics and Gynecology, Wayne State University School of Medicine, Detroit, Michigan, United States of America; 5 Biotechnology, Nirma Institute of Science, Nirma University, Ahmedabad, Gujarat, India; 6 Department of Genetics, Cell Biology & Anatomy College of Medicine, University of Nebraska Medical Center Omaha, Omaha, Nebraska, United States of America; New York Medical College, UNITED STATES

## Abstract

Tetralogy of Fallot (TOF) is the most common Critical Congenital Heart Defect (CCHD). The etiology of TOF is unknown in most cases. Preliminary data from our group and others suggest that epigenetic changes may play an important role in CHD. Epidemiologically, a significant percentage of CHD including TOF fail to be diagnosed in the prenatal and early newborn period which can negatively affect health outcomes. We performed genome-wide methylation assay in newborn blood in 24 non-syndromic TOF cases and 24 unaffected matched controls using Illumina Infinium HumanMethylation450 BeadChips. We identified 64 significantly differentially methylated CpG sites in TOF cases, of which 25 CpG sites had high predictive accuracy for TOF, based on the area under the receiver operating characteristics curve (AUC ROC) ≥ 0.90). The CpG methylation difference between TOF and controls was ≥10% in 51 CpG targets suggesting biological significance. Gene ontology analysis identified significant biological processes and functions related to these differentially methylated genes, including: CHD development, cardiomyopathy, diabetes, immunological, inflammation and other plausible pathways in CHD development. Multiple genes known or plausibly linked to heart development and post-natal heart disease were found to be differentially methylated in the blood DNA of newborns with TOF including: *ABCB1*, *PPP2R5C*, *TLR1*, *SELL*, *SCN3A*, *CREM*, *RUNX and LHX9*. We generated novel and highly accurate putative molecular markers for TOF detection using leucocyte DNA and thus provided information on pathogenesis of TOF.

## Introduction

Tetralogy of Fallot (TOF) is marked by a constellation of anatomical findings, including ventricular septal defect (VSD), pulmonary stenosis (PS), right ventricular hypertrophy and an overriding aorta. In the United States, the prevalence of TOF is estimated at 3.9 per 10,000 live births [1] and it accounts for 7–10% of congenital heart defect (CHD) cases. Further, TOF the most common critical congenital heart defect (CCHD) defined as CHDs that require surgical or other significant medical intervention before one year of age.[2]

Early prenatal and newborn diagnosis is, pivotal to reducing morbidity and mortality in CHD. Most cases of CHD occur in pregnancies with no identifiable risk factors for the disorder. A recent population-based screening study reported that nearly half of major CHD cases are missed on prenatal screening [3], this is consistent with other population- based studies reported in the literature. Effective prenatal screening will require markers with high diagnostic accuracy. In addition, a significant percentage of CHD continue to be missed in the newborn period. Indeed, over 50% of reported deaths in CCHD cases are due to missed or late diagnosis [4]. The current standard of care requires pulse oximetry screening of all newborns to detect CCHDs [5–7]. Despite official recommendations by the American Academy of Pediatrics and the Secretary of Health and Human Services among others, utilization of pulse oximetry is far from universal.[8] Further, simulation models suggest only modest detection rates for TOF using pulse oximetry screening.[9]

The etiology of TOF is unknown in most cases, however the disorder is believed to have a significant genetic component [10]. There is a strong association between TOF and 22q11 deletions [11] and aneuploidies, such as trisomy 21, 18 and 13. Mutations in a few genes have also been reported in TOF, including *NKX2-5*, *GATA4*, *ZFPM2*, *GATA6*, *GDF1*, *JAG1*, *TBX20*, *HAND1*, *HAND2*, *PITX2* and *TBX1*. [12–18] However, these mutations appear to be responsible for only a small fraction of TOF cases.

It is now clear that focusing on individual genes and gene mutations cannot provide a comprehensive picture of CHD pathogenesis [19] given the complexity of cardiogenesis which is impacted by an extensive list of environmental influences. The study of epigenetic contribution to CHD development is still relatively novel but has the potential to make significant contributions to our understanding of CHD pathogenesis. Epigenetic changes are largely tissue specific and the inaccessibility of the heart in living subjects is a monumental challenge to studying cardiac development in general. Work by our group [20, 21] and others [22] have reported evidence that a significant minority of epigenetic marks in blood DNA mirror similar changes in inaccessible organs such as the heart and the brain respectively. This opens the possibility of ongoing epigenetic analysis of such organs.

The objective of the current study was to analyze cytosine methylation marks in leucocyte DNA to investigate the pathogenesis of and for the detection of TOF in newborns. The study was limited to non-syndromic TOF cases.

## Methods

IRB approval was provided by the Michigan Department of Community Health (MDCH) and Wayne State University. The ethics committee waived the requirement for informed consent. The specimens were completely de-identified to the researchers prior to the analysis. DNA was extracted from archived dried neonatal blood spots collected for the newborn screening program run by the MDCH. The study subject consisted of 24 TOF cases and 24 controls matched for gestational age, gender, and maternal ethnic origin. Specimens were collected at 48 hours on average (range 24 and 79 hours) after birth. Demographic and other information was available for each subject including postnatal day of sample collection, maternal age and race, gestational age at birth and newborn sex. The average age of mothers was 29.8 years, average gestational age at birth was 38.8 weeks. Cases of known or suspected genetic syndromes, e.g. chromosomal or Mendelian or with extra-cardiac defects were excluded. An equal number of unaffected individuals were used as controls. To reduce potential variability due to race only Caucasian, non-Hispanic mothers were included in this pilot study.

### Epigenome-wide methylation scan using the Infinium methylation 450 array BeadChips

Multiple prior studies have reported genome-wide DNA methylation profiles from archived dried blood spots using Infinium HumanMethylation450 BeadChips. [20] [21]. The Infinium HumanMethylation450 BeadChips array for methylation (Illumina, Inc., California, USA) contains >485,000 CpGs per sample in enhancer regions, coding regions, promoters and CpG islands at a single-nucleotide resolution and requires only 500 ng of genomic DNA per assay. These sites include 96% of RefSeq genes and 95% of CpG islands. DNA samples for this study were bisulfite converted using the EZ DNA Methylation-Direct Kit (Zymo Research, Orange, CA), and fluorescently-stained BeadChips imaged by the Illumina iScan. Data was analyzed with Illumina’s Genome Studio methylation analysis package program. Prior to bioinformatic and statistical analysis, data preprocessing and quality control was performed including examination of the background signal intensity of negative controls, the methylated and unmethylated signals, and the ratio of the methylated and unmethylated signal intensities. The processing was done according to manufacturer’s protocol, and 99% of the CpG loci are determined unequivocally.

To avoid potentially confounding factors including sex-specific methylation bias, CpG sites on the sex chromosomes and those containing SNPs in the probe sequence (listing dbSNP entries near or within the probe sequence, i.e., within ten bp of the CpG site) were excluded from further analysis.[23–25] CpG targets associated with SNPs near or within the probe sequence may influence corresponding methylated probes.[26]

### Statistical and bioinformatics analysis

Following the preprocessing described above, a DNA methylation level or ß-value was assigned to each remaining CpG site. Differential methylation was assessed by comparing the ß-values per individual nucleotide at each evaluated CpG locus between CHD subjects and controls. The p-values for methylation differences between case and control groups at each locus were calculated as previously described.[27] Filtering criteria for p-values was set at <0.001 to enhance for the most discriminating cytosine loci. P-Values were computed with and without False Discovery Rate (FDR) correction for multiple testing (Benjamini-Hochberg test). Data were normalized using the Controls Normalization Method.

The most significant CpG sites based on p<value threshold was further sub-selected based on the pre-set cutoff criteria of >2.0-fold methylation increase or >2.0-fold decrease. The CpG sites with the highest (hypermethylated) and lowest (hypomethylated) degree of methylation were further analyzed. The significantly differentially methylated CpG sites in TOF cases were subsequently used to generate a heatmap using the Complex Heatmap (v1.6.0) R package (v3.2.2).[28, 29] We used ward distance for the hierarchical clustering of samples. Subsequently, the ß-value thresholds of each CpG locus was used to calculate the area under the receiver operating characteristics curve (AUC) and 95% confidence intervals for TOF detection. All data cleaning and analysis were performed using R version 3.2.3.

### Principal Component Analysis (PCA)

To analyze larger number of variables to a smaller number of factors between TOF and controls, we performed Principal Component Analysis. We removed all probes which have missing ß-value, remaining beta value of CpG targets were used for PCA. We used R function “prcomp” to compute principal component (PC), and used PC1 and PC2 for the plot. The 3D plot was generated using R package “ggplot2”.

### Gene ontology analysis and functional enrichment

Literature data mining for co-occurrence of gene names and keywords of interest were performed using Chilibot (www.chilibot.net). Only genes for which Entrez identifiers are available were further analyzed. Genes found to be differentially methylated (at an FDR p-value threshold ≤0.01) were processed through QIAGEN’S Ingenuity Pathway Analysis (IPA) software (Ingenuity Systems, www.ingenuity.com) to identify biological functions or interacting regulatory networks. This provides information on the pathogenesis of the CHD. All CpGs without mapping IDs in IPA (HG19, release 2009) were excluded from analysis, which included gene ID conversion, bio-pathways analysis, and determination of the molecular functions of methylated and unmethylated regions. Over-represented canonical pathways, biological processes, and molecular processes were identified.

### Validation of methylation status by bisulfite sequencing

To further validate the array data, we used pyrosequencing to analyze the DNA methylation status of the most significant CpG sites. We performed pyrosequencing analysis of bisulphite-converted DNA with appropriate oligos using the PyroMark Q24 System and advanced CpG Reagents (Qiagen^®^). Briefly, 500 ng of genomic DNA from each sample was bisulfite-treated using the EZ methylation kit (Zymo Research) as per the manufacturer’s instructions and amplified by a bisulfite polymerase chain reaction, followed by Quantitative DNA methylation analysis of each CpG.

### Analysis of correlation of cytosine methylation with gene expression and chromatin conformation of CpG sites

A limitation of the study was that we were unable to perform corresponding gene expression analysis to determine how DNA methylation correlated with gene expression given the fact that only archived blood spots were available to us. We therefore looked at whether methylation status of the CpG loci of interest is known to correlate with gene expression levels in other tissues. The GDAC FIREHOSE series of databases have been developed by the Broad Institute using RNA-Seq data from over 20 types of malignant tissue (diffuse large B-cell lymphoma, prostate adenocarcinoma-primary tumor, pancreatic adenocarcinoma-primary tumor, acute myeloid leukemia, ovarian cancer, etc.) and include hypermethylation data at the nucleotide level correlated with the expression mean of the gene in which the CpG site is located (gdac.broadinstitute.org). Each of the identified differentially methylated CpG sites was examined for inclusion in GDAC Firehose and its coordinates (correlation coefficient, p-value, methylation mean, and expression mean) were noted. Each CpG site was also examined in the ENCODE databases using the UCSC genome browser for its location, type of location (e.g., promoter, intron, etc.), presence or lack of *H3K27AC* laying, and transcription factor occupancy (e.g., *PolR2a*, *PHF8* etc.) which indicate a potential role in gene expression.

## Results

### Identification of differentially methylated CpG sites

**Fig 1:**
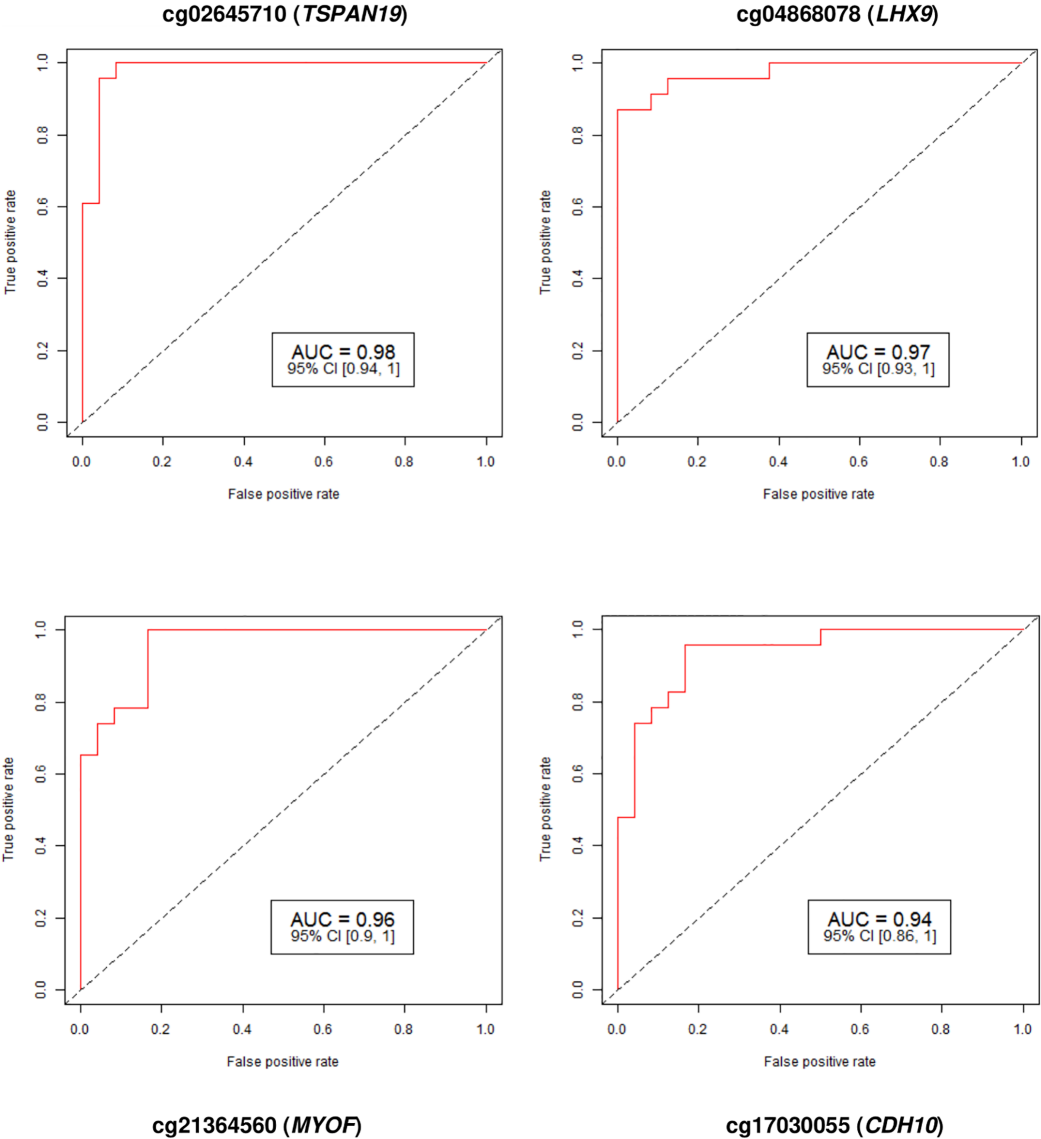
Receiver operating characteristic (ROC) curve analysis of methylation profiles for four specific markers associated with Tetralogy of Fallot. We identified 64 differentially-methylated CpG sites in 64 genes that have an area under the ROC curve ≥0.75 for TOF prediction. At each locus, the False Detection Rate p-value for the methylation difference between TOF subjects and controls was highly significantly different. Due to figure resolution concerns, we have included only four markers (chr 12; cg02645710) (chr 1; cg04868078) (chr 10; cg21364560) (chr 5; cg17030055). AUC: Area Under the Receiver Operating Characteristics Curve; 95% CI: 95% Confidence Interval. Lower and upper Confidence Intervals are given in parentheses.

Sixty-Four CpG sites, corresponding to 64 genes, were differentially methylated either in the coding and/or promoter regions. This included 9 hypomethylated and 55 hypermethylated CpG sites. The identified CpG sites, associated genes and chromosome locations are listed in Table 1. The methylation levels of cases and controls, fold change and FDR p-value of the difference in methylation levels are also provided. The AUC (95% CI) for predicting TOF based on methylation level at each of the cytosine loci is included. An AUC ≥0.75 generally indicates a screening marker that could potentially have clinical utility. High predictive accuracy for TOF detection was achieved at multiple CpG targets (Fig 1). In addition, the ROC curves for the genes *RUNX1* and *CREM* with low methylation difference also given (S1 Fig). The FDR p-values for the methylation difference between TOF subjects and controls were highly significant. Overall, a total of 25 CpG loci in 25 genes had excellent predictive accuracy (AUC ≥ 0.90) for the detection of TOF. Principal Component Analysis (PCA) results showed that there is a clear variance between two components. Majority of TOF components fall away from controls (S2 Fig). Based on PCA, a subset analysis was performed using 8 TOF cases and 24 controls with a clear separation (S1 Table). This subset analysis identified a total of 2390 targets including 57 CpGs targets initially identified using 24 cases and 24 controls. A boxplot with clear methylation differences over all the candidate CpGs is provided in S3 Fig.

**Table 1:** Differentially methylated CpG loci and genes. Target ID, Gene ID, chromosome location, % methylation change and FDR p-value for each gene methylated. CpG sites with significant False Detection Rate p-value indicating methylation status and area under the receiving operator characteristic curve ≥0.75.

Target ID	Gene	CHR	% Methylation		Fold Change	FDR p-Val	AUC	CI_upper	CI_lower
			Cases	Controls					
cg27120934	LAMA2	6	8.07	19.08	0.42	9.04E-26	0.98	1.00	0.95
cg02645710	TSPAN19	12	36.68	17.11	2.14	7.31E-45	0.98	1.00	0.94
cg04868078	LHX9	1	34.02	13.36	2.55	1.81E-45	0.97	1.00	0.93
cg23680282	LRRIQ1	12	17.31	6.17	2.81	1.12E-40	0.97	1.00	0.92
cg25697769	JOSD1	22	4.01	8.63	0.46	4.97E-09	0.97	1.00	0.93
cg21364560	MYOF	10	37.07	15.97	2.32	1.03E-45	0.96	1.00	0.90
cg24132989	C6orf162	6	22.32	45.01	0.50	1.17E-29	0.96	1.00	0.90
cg14905634	TRHDE	12	15.37	32.00	0.48	3.66E-31	0.95	1.00	0.88
cg08821669	COX6A1	12	3.80	8.49	0.45	1.97E-09	0.95	1.00	0.88
cg02062326	TMED10	14	24.46	12.16	2.01	3.35E-41	0.94	1.00	0.87
cg11641791	KRT222	17	27.02	12.77	2.12	4.02E-42	0.94	1.00	0.87
cg11792281	NLK	17	11.65	23.41	0.50	1.05E-23	0.94	1.00	0.87
cg12414181	SCAMP5	15	7.54	18.61	0.40	8.16E-27	0.94	1.00	0.87
cg17030055	CDH10	5	26.43	11.55	2.29	1.96E-42	0.94	1.00	0.86
cg17728974	LIN28B	6	17.38	7.24	2.40	3.15E-40	0.94	1.00	0.87
cg13114458	KRR1	12	12.43	5.54	2.25	7.47E-39	0.93	1.00	0.85
cg18803079	EFCAB7	1	11.34	24.22	0.47	1.12E-27	0.93	1.00	0.86
cg23274377	BPNT1	1	15.19	6.87	2.21	1.92E-39	0.93	1.00	0.85
cg01311718	IKZF2	2	14.54	6.83	2.13	3.45E-39	0.92	1.00	0.84
cg02609279	ITGA4	2	16.61	8.09	2.05	1.56E-39	0.92	1.00	0.83
cg02071276	FBXO28	1	20.60	9.15	2.25	8.18E-41	0.91	1.00	0.82
cg15946310	TTC1	5	16.42	6.48	2.53	3.82E-40	0.91	1.00	0.83
cg18295068	SCN3A	2	21.99	10.96	2.01	1.27E-40	0.91	1.00	0.83
cg09365677	CHRM3	1	24.05	9.68	2.49	3.49E-42	0.90	1.00	0.81
ch.11.319992F	USP47	11	19.88	9.22	2.16	1.85E-40	0.90	1.00	0.81
cg12092090	CACNA1A	19	18.09	7.95	2.28	3.12E-40	0.89	1.00	0.79
cg19533977	CLTC	17	15.05	7.47	2.01	3.89E-39	0.89	1.00	0.79
cg20101489	SCG2	2	23.03	10.35	2.23	2.22E-41	0.89	1.00	0.80
cg01400516	NETO2	16	7.05	3.03	2.33	1.65E-08	0.89	1.00	0.79
cg23134869	ZFHX4	8	12.44	6.19	2.01	1.60E-13	0.89	1.00	0.80
cg08264335	SELL	1	15.67	7.72	2.03	2.75E-39	0.88	1.00	0.77
cg25477497	ABCB1	7	14.42	6.98	2.07	4.46E-39	0.88	1.00	0.79
cg25947619	AKAP13	15	18.92	8.64	2.19	2.72E-40	0.88	1.00	0.78
cg14534336	JMJD1C	10	9.75	4.78	2.04	2.70E-10	0.88	1.00	0.78
cg07002382	MFAP1	15	13.01	5.98	2.17	6.61E-39	0.88	1.00	0.78
cg12273284	CAMK1D	10	13.19	4.99	2.65	2.12E-39	0.87	1.00	0.77
cg10225640	ANAPC5	12	10.02	4.55	2.20	2.41E-12	0.86	1.00	0.75
ch.1.3587792F	SMG7	1	25.97	12.81	2.03	1.32E-41	0.86	1.00	0.76
cg03814610	FAM13A	4	13.86	5.99	2.32	2.93E-39	0.84	1.00	0.72
cg10558887	SPG20	13	16.51	7.12	2.32	6.67E-40	0.84	1.00	0.73
cg18469624	PRKG1	10	19.24	9.52	2.02	4.75E-40	0.84	1.00	0.73
cg19579903	PPP1R10	6	14.04	5.79	2.42	2.05E-39	0.84	1.00	0.73
cg17616217	KY	3	3.29	7.05	0.47	5.42E-07	0.84	1.00	0.73
cg03547245	MSI2	17	10.26	4.40	2.33	5.14E-14	0.83	1.00	0.71
cg12129209	PPP2R5C	14	12.68	6.00	2.11	2.42E-15	0.83	1.00	0.71
cg23404012	MED6	14	23.20	11.40	2.03	5.68E-41	0.83	1.00	0.71
cg03846926	C10orf140	10	16.64	7.20	2.31	6.36E-40	0.82	0.90	0.70
cg04254487	TBPL1	6	23.75	11.35	2.09	3.01E-41	0.82	0.90	0.69
cg08757862	TLR1	4	16.97	7.39	2.30	5.51E-40	0.82	0.90	0.69
cg26800788	PDE4D	5	18.92	8.92	2.12	3.60E-40	0.82	0.90	0.69
cg10944144	ADAMTS6	5	13.66	5.77	2.37	2.90E-39	0.81	0.90	0.69
cg02981003	GPR123	10	15.51	7.72	2.01	3.19E-39	0.81	0.90	0.69
cg26401673	ANO10	3	16.56	7.78	2.13	1.22E-39	0.80	0.90	0.67
ch.2.800013F	BIRC6	2	11.69	5.53	2.12	6.69E-14	0.80	0.90	0.67
cg06237608	CHD2	15	7.92	3.49	2.27	1.53E-09	0.80	0.90	0.68
cg02558537	CWF19L2	11	10.03	4.97	2.02	2.08E-10	0.78	0.90	0.64
cg11378242	FAM36A	1	18.40	8.56	2.15	4.22E-40	0.78	0.90	0.65
cg17485454	MAPK10	4	16.60	8.24	2.01	1.84E-39	0.78	0.90	0.65
cg04838988	PRDM14	8	26.05	8.50	3.06	8.83E-44	0.77	0.90	0.63
cg19021985	PPP3CC	8	36.66	18.23	2.01	3.05E-44	0.76	0.90	0.62
ch.1.659794R	UBR4	1	13.09	6.08	2.15	2.15E-16	0.76	0.90	0.63
cg27509202	CREM	10	9.96	4.96	2.01	3.24E-10	0.76	0.90	0.62
cg00994804	RUNX1	21	13.65	6.17	2.21	4.28E-39	0.76	0.90	0.62
cg22664298	ADAMTS19	5	18.76	9.24	2.03	5.81E-40	0.75	0.90	0.61

### Cluster analysis of differentially methylated targets

**Fig 2:**
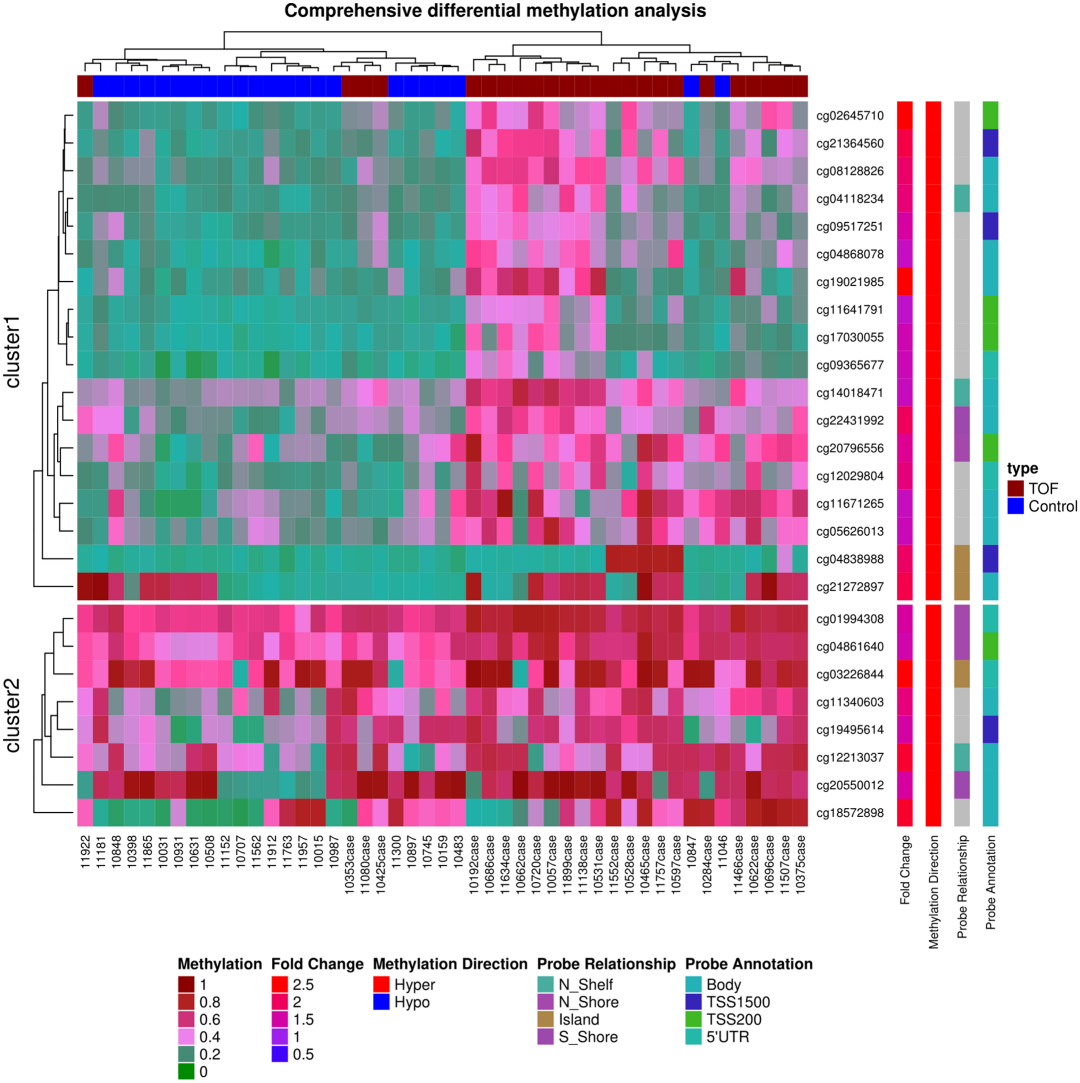
Heatmap of 26 highly differentially methylated loci. DNA methylation profiling based on unsupervised hierarchical clustering identified four unique clusters that have distinct methylation signatures. These 26 targets represent the most highly differentially methylated loci (False Detection Rate < 0.000001). Only CpG sites shown are those with either 2.0-fold hypomethylated or 2.0-fold hypermethylated in the disease TOF cases (red colored squares) compared to control subjects (blue colored squares). The figure also displays direction, fold change in disease, probe relationship and probe annotation of differentially methylated CpG sites. The red color in the heatmap indicates hyper DNA-methylation and blue hypo DNA-methylation values.

Of these 64 differentially methylated genes, unsupervised hierarchical clustering analysis demonstrated their distribution as 4 major clusters (Fig 2). The methylation level of each CpG cluster visually differentiated TOF versus controls. In cluster 1, we identified 8 significant novel CpG sites (and genes) [cg02645710 *(TSPAN19)*, cg04868078 *(LHX9)*, cg21364560 *(MYOF)*, cg11641791 *(KRT222)*, cg17030055 *(CDH10)*, ch.1.3587792F *(SMG7)*, cg19021985 *(PPP3CC)*, and cg04838988 *(PRDM14)*] associated with TOF. Most of the related CpG sites had ≥10% including (N = 51) methylation difference between TOF and controls indicating a higher biological likelihood for being biologically significant.

### Chromatin conformation and transcription factor occupation

**Fig 3:**
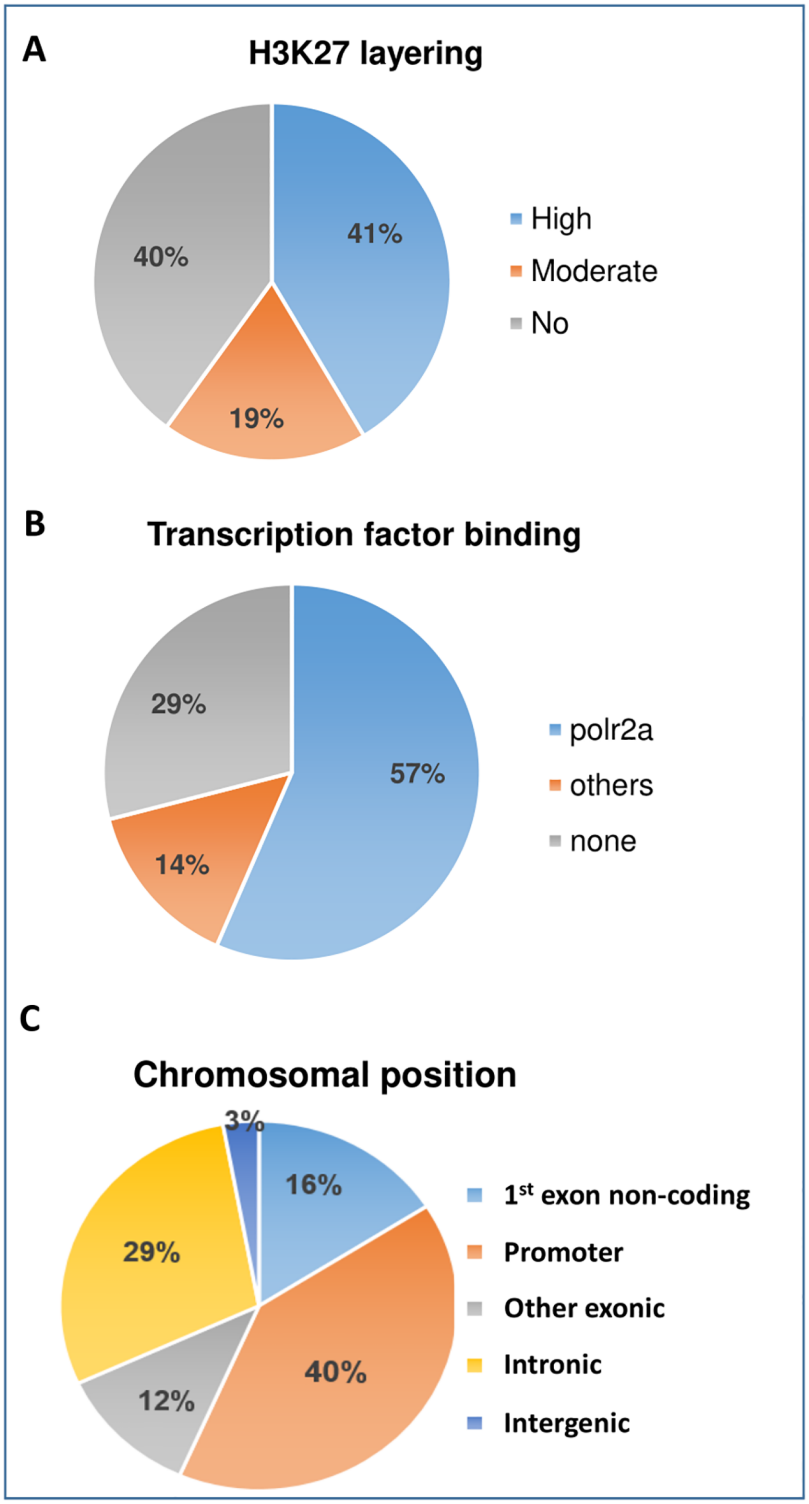
Open chromatin and transcription factor occupancy in functional CpG sites. Pie charts depicting H3K27Ac histone mark layering, location of CpG sites and transcription factor binding sites. A) The layering of H3K27Ac histone mark conferring an open chromatin status was seen in 87% of sites. B) 57% of the 64 sites were occupied by RNA polymerase II subunit RPB1. C) 57% of 64 differentially methylated functional CpG sites were in the promoter region of genes. See S2 Table for confirmation data per each gene.

Transcription initiation or regulatory sites often present an open chromatin conformation that permits binding of regulatory transcription factors. These sites may reside in the promoter of the gene, 1^st^ exon, and intron or even outside of the gene. We observed that 40% (n = 28) of the 39 CpG sites were in the promoter region of the gene, and 16% (n = 11) were in the noncoding area of the 1^st^ exon. A modest number of CpG sites were in an intron (29%, n = 20). A detailed analysis of each CpG site with the ENCODE databases suggested that 81% of 64 CpG sites (high 40% plus moderate 41%) were layered with H3K27Ac (Fig 3A), an open chromatin conformation that is accessible to transcription factors during transcription initiation. Further analysis showed that 57% (n = 39) of these CpG sites were occupied by RNA *PolR2a* (RNA polymerase), a transcription initiator. Other transcription factors (TFs), such as *PHF8*, *ERBB2*, *MYC*, *YY1*, *EGR2*, etc. were also present. We have identified 37 TFs overlap the candidate CpGs. Among these, 25 TFs are cardiac TFs (S2 Table, Fig 3B). A smaller number of methylated CpG sites were in coding exon and intergenic sequences (S2 Table) (Fig 3C).

In some hypermethylated CpG sites (n = 10, 14%), non-Pol2R2A-dependent transcription initiation or other accessory transcription mechanisms could possibly be involved, such as DNA looping. However, some CpG sites are not occupied by any transcription factors suggesting that either the ENCODE catalog of transcription factors may not be sufficiently comprehensive or these CpG sites may not have a major role in regulating transcription.

### Modulation of gene expression

To assess whether the observed methylation changes in each CpG in our study are likely to modulate gene expression, we reviewed the Broad Institute Firehose methylation-expression correlation databases, where CpG methylation changes in the genome have been correlated with gene expression using RNA sequencing data. This would provide evidence as to whether the observed variabilities in methylation changes in these CpGs indeed influence gene expression changes.

We found in all cases (S3 Table) that the correlation coefficient of mean methylation changes vs mean expression changes was negative, indicating that hypermethylation in these sites was associated with a reduction of expression, and hypomethylation with an increase in expression. Thirty-nine of 64 of our observed differentially methylated CpG sites are present in the database, with the extent of methylation changes correlated with the extent of expression changes of the corresponding genes. Among these, 28 (44%) of the 64 CpG sites have alterations in cytosine methylation located within the promoter of the gene. Additional CpG sites were found to reside in other regions besides the promoter, including noncoding regions or coding 1^st^ exons (n = 11, 17%), which are also assumed to regulate gene expression [30] and are also correlated with expression changes. Thus, evidence that reduction of methylation in these 39 CpG sites increases the gene expression in tissues analyzed with RNA sequencing in these databases, suggests that similar alterations may take place in the blood cells that we used in our study, which could have implications in the development of TOF phenotypes.

As there were no available RNA samples from the study subjects, we have used already available RNA-seq data from heart tissues of TOF patients and normal heart controls. The data published in Grunert et al., 2014 and 2016 [31, 32] was compared with our data. Interestingly we have identified *ADAMTS6* gene matched with their data and was found to be differentially expressed. Additionally, we have matched our differentially methylated genes with the Grunert et al., 2016 and identified 25 genes (S4 Table).

### Association with known cardiovascular disease pathways

**Fig 4:**
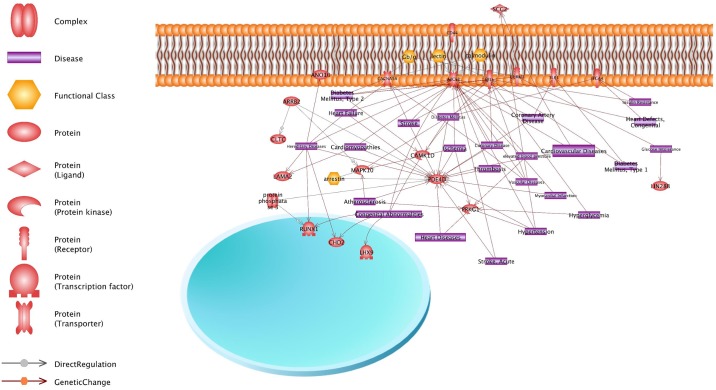
Pathways analysis of significant DNA methylation variations and network analysis. Ingenuity pathway analysis (IPA) results for gene sets that were most highly differentially methylated in association with TOF. IPA results indicated the gene network is relevant to immunological, toxicity, nociception, metabolic, receptor, cell signaling, and inflammation pathways.

Genes were further grouped per their Gene Ontology (GO)-characterized function. GO analysis identified biological processes and roles for these genes including immunological pathways, toxicity pathways, expression target pathways, nociception pathways, metabolic pathways, receptor signaling, cell signaling and inflammation pathways (Fig 4). The Ingenuity Pathway Analysis (IPA) identified important genes associated with these CpG sites that are currently known or suspected to be associated with cardiac disease either congenital or developing in postnatal life. The genes are associated with various postnatal cardiovascular disorders such as Type 1 and type 2 diabetes, stroke, atherosclerosis, congenital heart defects, ischemia, coronary artery diseases, high blood pressure, myocardial infarction, and vascular thrombosis.

## Discussion

In the present study, we identified significant differences in methylation levels of multiple CpG loci in TOF versus controls. We found 64 CpG sites in 64 genes that were significantly differentially methylated in TOF versus controls. Among 64 differentially methylated CpGs, 55 were hypermethylated and only 9 were found to be hypomethylated. We have used top 26 hypermethylated CpGs to generate heatmap (Fig 2). Many of these CpG loci are in genes that are already known or suspected to be involved in CHD development or postnatal cardiovascular disorders. Some of the genes we identified have not however been previously reported to be associated with TOF and CHD and require further evaluation. The difficulty of accurate prenatal and newborn diagnosis of CHD is well established in the literature [3,4,9]. Using DNA methylation, we identified many important CpGs that preliminarily demonstrate high diagnostic accuracy for TOF detection (Table 1). In the future, these CpGs could have clinical utility for TOF detection.

Leenen et al. [33] suggested that even relatively small differences in the methylation level, e.g. of <10%, could be associated with changes in gene expression and phenotype. In the present study, we have observed methylation variation between TOF and controls in 51 CpG targets with ≥10%.

We did not have access to fresh blood samples to perform DNA methylation at CpG sites and gene expression correlation from the same patient. Instead we interrogated the Broad Institute Firehose database to see whether methylation in CpG sites have been shown to correlate with expression. This database uses cancer tissue from various organs, e.g. ovary, breast and colon. We found that 39 of the 64(56.5%) CpG sites, methylation levels correlate with gene expression in these tissues. Also, using the ENCODE database, which was created using normal tissues/cell lines, we found that 81% of differentially methylated CpG sites reported conformed with H3K27AC occupancy at the same site. The H3K27AC binding reflects an open chromatin formation permitting the binding of transcription factors and gene transcription. The CpG sites with significant methylation changes in TOF are not only located within the CpG islands in the promoter region of genes, but were also distributed throughout the gene including transcription start site(s) (TSS), TSS200 (region from TSS to 200 bp upstream of TSS), TSS1500 (200–1,500 bp upstream of TSS), 1^st^ Exon, Body (coding), 5’ UTR, and 3’UTR regions. Moreover, when we compared our data with previously studied differentially expressed and methylated regions using the myocardial biopsies, 25 genes were found to be reproduced with differential methylation and one gene with expression variation in our study [32]. We conclude from the above results that altered expression of one or a combination of the 64 CpG sites in 64 genes is likely to be associated with altered expression of critical cardiac developmental genes and possibly linked with TOF development.

Although genome- wide DNA methylation tends to be highly tissue specific, significant correlation in DNA methylation profiles across tissues are now being found in a small but important minority of CpG loci e.g. between blood and brain [22, 34]. The cause of these so called ‘mirror’ sites could be the result of epigenetic reprogramming that affected germ lines and embryogenesis. In addition, blood leucocytes could be modified epigenetically as they circulate through an affected primary organ. Further, genetic polymorphisms that can alter methylation profile in more than one tissue and disease causing environmental factors that alter the epigenetic profile in several tissues simultaneously are all potential explanations for synchronized DNA methylation changes that have been observed when different tissues have been compared.[35]. Our prior studies suggest that in similar fashion, leucocyte DNA might reflect cardiac epigenetic changes in CHD including TOF [20, 21]. Our analysis suggests a novel, non-invasive and potentially highly impactful approach for the study of cardiac development and CHD.

### Genes associated with congenital heart defects and postnatal cardiovascular disorders

As noted, prior work by our group found highly significant alterations in CpG methylation in blood leucocyte DNA in multiple different types of CHD including TOF. Sheng et al. conducted a targeted promoter region methylation study using MassARRAY-based quantitative analysis in the myocardium of patients with TOF and reported DNA methylation changes. Both studies confirm that TOF is associated with significant DNA methylation changes.

In the current study, the genes identified are involved in DNA damage response, toxicity pathways, expression targets pathways, Notch signaling pathways, metabolic pathways, receptor signaling, cell signaling and inflammation pathways. Pathways over-representation analysis suggests that these genes could play a significant role in TOF development. All identified CpG methylation sites are within or in close vicinity to genes whose functions have been associated with cardiac development. These genes include *ANO10*, *ABCB1*, *ARRB2*, *CLTC*, *LAMA2*, *RUNX1*, *CHD2*, *LHX9*, *PRKG1*, *PDE4D*, *CAMK1D*, *SELL*, *SCG2*, *CHRM3*, *TLR1*, *LIN28B*, *MAPK10*, *ITGA4* and *CACNA1A*.

*ABCB1* (OMIM 171050) also known as P-glycoprotein 1 (*PGY1*) and Multidrug Resistance 1 (*MDR1)*, *is a* gene encoding for P-glycoprotein, a large transmembrane protein. Significant correlations between *ABCB1* gene polymorphism and increased risk of congenital heart defects,[36] hypertension,[37] stroke, and thrombosis [38] have been previously reported.

The *PPP2R5C* (OMIM 601645) gene on 14q32.31 is a phosphatase 2A regulatory subunit B-family gene involved in negative control of cell growth and cell adhesion in the endothelial lining, and cardiac local signaling.[39] The protein also plays a critical role in cardiomyocyte development thus a role in cardiac and TOF abnormalities is highly plausible.[40]

### Genes associated with diabetes

The *PPP2R5C* (OMIM 601645) gene on 14q32.31 also plays an important role in diabetes development.[41]. Two *TLR1* (OMIM 601194) or Toll-like receptor (*TLR*) on 4p14 and *SELL* (OMIM 153240) or L-Selectin on chromosome 1q24.2 are candidate genes for the development of type 1 diabetes. The *CAMK1D* (OMIM 607957) gene on 10p13 and *CACNA1A* (OMIM 601011) on 19p13.13 are also associated with diabetes. Maternal pregestational diabetes is a potent risk factor for the development of congenital heart defects in offspring.[42, 43]

The *SCN3A* (OMIM 18239) gene on 2q24.3 is a sodium-dependent channel protein that plays a major role in pulmonary artery smooth muscle and cardiomyocyte excitation.[44] Moreover, this protein acts as a coupler of protein-protein interactions in a centrosome-cilium formation that is distinctly related to congenital heart abnormalities.[45] The role of embryonic cilia in developing congenital heart malformation is well established, making a role in TOF development plausible.

The *CREM* (OMIM 123812) gene on chromosome 10p11.21 is a transcription factor that binds to a cAMP-responsive element in the promoter. Overexpression of cAMP-response element modulator causes abnormal growth and development of the atrial myocardium and leads to atrial fibrillation in mice.[46] and affects cardiac remodeling in mice[47] and the development of myocardial infarction in rats.[48] *CREM* is involved in the switching of ß-adrenergic receptor signaling in the decision for cell survival or death in cardiomyocytes. [49]

The *LIM* homeobox gene 9 (*LHX9*; OMIM 606066) is on 1q31.3. *Lhx9* is essential for the formation of the epicardium and heart development. Smagulova et al. demonstrated that the mouse *Lhx9* gene is the direct target of the *GATA4/FOG2* repressor complex important in the developing mouse heart.[50]. Further study will be needed to establish whether the differential expression this homeobox gene could play a major role in developing TOF.

The *ITGA4* gene (OMIM 192975) that maps to chromosome 2q31.3 is known to play a distinct role in epicardial and coronary vessel formation.[1] In *Itga4*-/- mouse, the epicardium detaches from the myocardium and degrades. *ITGA4* acts as fibronectin receptor and *ITGA4* influences epicardial Fn1 polymerization. *ITGA4* overexpression alters Fibronectin/ integrins interactions and disrupts fibronectin deposition leading to epicardial dysmorphology and coronary malformation.

Laminin Alpha-2 (*LAMA2*) (OMIM 156225 0) on chromosome 6q22.33 is a laminin protein that is conspicuously present in cardiomyocytes and plays a major role in cardiac development. A mutation in the laminin gene causes dilated cardiomyopathy and a spectrum of heart abnormalities.[51, 52]. Myoferlin *(MyoF)* is a ferlin family member protein located in the plasma and nuclear membrane that plays a major role in VEGFA secretion and causes cardiac muscle weakness in diabetes.[53]

The *RUNX1* gene (OMIM 151385) on chromosome 21q22.12 is a transcription factor that binds with promoters and enhancers in many genes. *Runx1* copy number variation is associated with congenital heart malformation[54] including TOF[55] and thus differential methylation leading to aberrant expression of this gene in TOF is significantly plausible.

TOF is well known to be linked to chromosome 22q11.2 deletions [56]. However, in the present analysis, we have not observed any gene variation(s) in this region except in the *JOSD1* gene. Given the relatively small number of cases used in this study, however, and the high threshold used for significance that was used, more subtle changes in other CpG loci of other genes could have escaped detection. Known or suspected cases with syndromic heart defects including 22q deletions, chromosomal abnormalities, or extra cardiac abnormalities—which increase the likelihood of genetic abnormalities were excluded from this study.

This manuscript does not posit that epigenetic changes are the only molecular changes that are occurring in these non-syndromic CHD cases. Indeed, recent evidence suggests that single nucleotide polymorphisms (SNP) induce and are associated with DNA methylation changes in neighboring cytosine nucleotides. In addition, copy number variations are known to be associated with alteration of DNA methylation profiles.[57] It is therefore to likely that there is significant overlap between DNA methylation and molecular pathologies. Thus, while a genetic basis has been established in about a third of CHD cases,[58] this could be an underestimation. Our cases excluded known or suspected genetic causes of TOF such as chromosomal anomaly. In addition, our study group consisted of sporadic cases of TOF reducing the influence of genetic causes. Given the very high frequency of epigenetic changes in our isolated CHD cases in comparison to controls it is possible that a significant percentage could have had only epigenomic changes inducing the CHD. Taken together these data would suggest a further layer of complexity where DNA methylation can cause, or result from, or occur in concert with other molecular pathologies. While sequencing and microarray analysis of the DNA would be desirable to measure the extent of overlap between epigenomic and sequence and CNV changes, this was not the objective of our study and we did not have IRB approval to perform such analyses.

Blood is heterogenous and consists of different (leucocyte) cell types which are likely to have different epigenetic profiles. Likewise, cardiac tissue has multiple cell types with different methylation profiles, thus the correlation between methylation status of different leucocyte and cardiac cell subtypes is likely to vary, adding another potential layer of complexity. It is reasonable however to assume that unless the presence of cardiac defect alters the composition of leucocytes in the blood (no evidence of this exists to our knowledge) that the observed differences in the average methylation status of leucocytes between TOF and controls as assessed in our study, was not in fact due to differences in methylation status of the different leucocyte sub-populations but rather due to or associated with the TOF itself.

A limitation of our study was that expression analysis could not be performed since we used archived blood spots. Analysis of Broad Institute Firehose and ENCODE databases suggest that the methylation levels of most of the important CpG sites identified in our study likely correlates with gene expression levels. We plan RNA-seq experiments in future studies to determine the correlation between DNA methylation and gene expression in CHD samples. Another limitation is that the relatively small sample size means that the results, involved genes, pathways and diagnostic accuracy for TOF detection are not definitive.

## Conclusions

The use of epigenetics to understand the mechanisms of heart defects is in its relative infancy and promises to help advance our understanding of these malformations. The identification of the causative mechanisms in CHD will not only improve understanding of disease mechanisms but could in the future contribute to the development of disease prophylaxis and therapy.

The present study provides new target genes and cellular pathways potentially involved in TOF development based on DNA methylation analysis based altered DNA methylation analysis. Although not definitive, our results highlight the potential importance of epigenetics in the pathogenesis of TOF. Finally, cardiac tissue is largely inaccessible in living fetuses and children so analysis using surrogate tissue such as blood could dramatically change our ability to detect and evaluate CHD.

## Supporting information

S1 Fig Receiver operating characteristic (ROC) curve analysis of methylation profiles for two specific markers (*RUNX1* and *CREM*) with low methylation difference associated with Tetralogy of Fallot. (TIF)

S2 Fig Three dimensional PCA (PCA 3D) TOF and control samples. (TIFF)

S3 Fig A boxplot with clear methylation differences over all the candidate CpGs. (TIFF)

S1 Table Subset analysis of 8 cases and 24 controls based on PCA clear differentiation. (PDF)

S2 Table Open chromatin conformation and transcription factor binding in differentially methylated CpG sites indicated their role in transcription initiation. ENCODE data showing the H3K27Ac layering on each CpG site presenting an open chromatin conformation. These CpG targets were also occupied with various transcription initiation factors, mostly *PolR2A*. The position of each CpG site was also noted in respect to the gene in which it resided. Some of the CpG sites that were differentially methylated resided in intronic or 1^st^ exonic regions, signifying their essential function in modulating transcription.
(PDF)

S3 Table Correlation of Methylation mean with the expression mean in various human tissues from GDAC data. 39 differentially methylated CpG targets were correlated with expression (RNA-seq) data. A bar chart was generated for each CpG target showing the proportion of methylation and mean of expression of the gene in which the CpG target resided.
(PDF)

S4 Table Differentially methylated CpG overlap with DMRs of Grunert et al., 2016. (PDF)
