# Supplementary figures and images for: Newborn blood DNA epigenetic variations and signaling pathway genes associated with Tetralogy of Fallot (TOF)
Source: PLoS One. 2018 Sep 13;13(9):e0203893. doi: 10.1371/journal.pone.0203893 (PMC6136787; doi:10.1371/journal.pone.0203893)

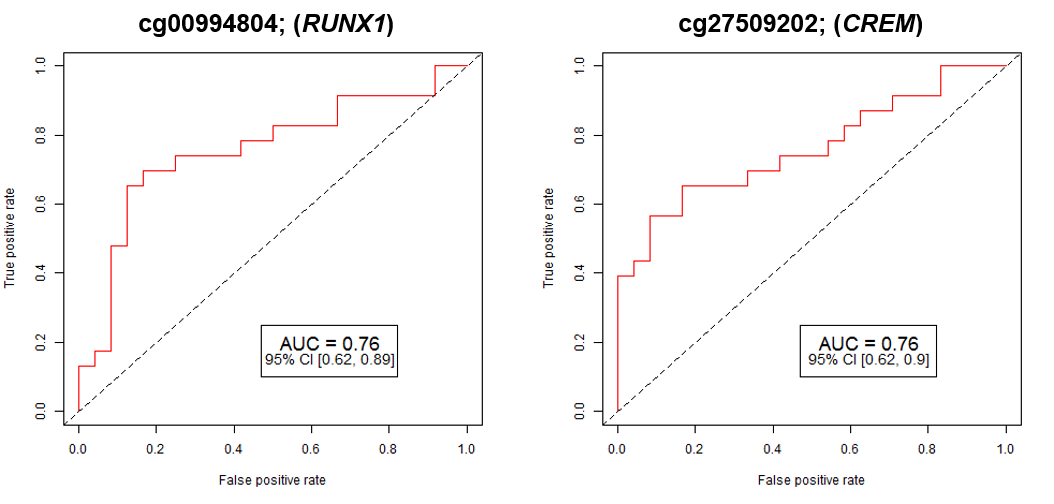

Supplement: S1 Fig — (TIF) [file pone.0203893.s001.tif]

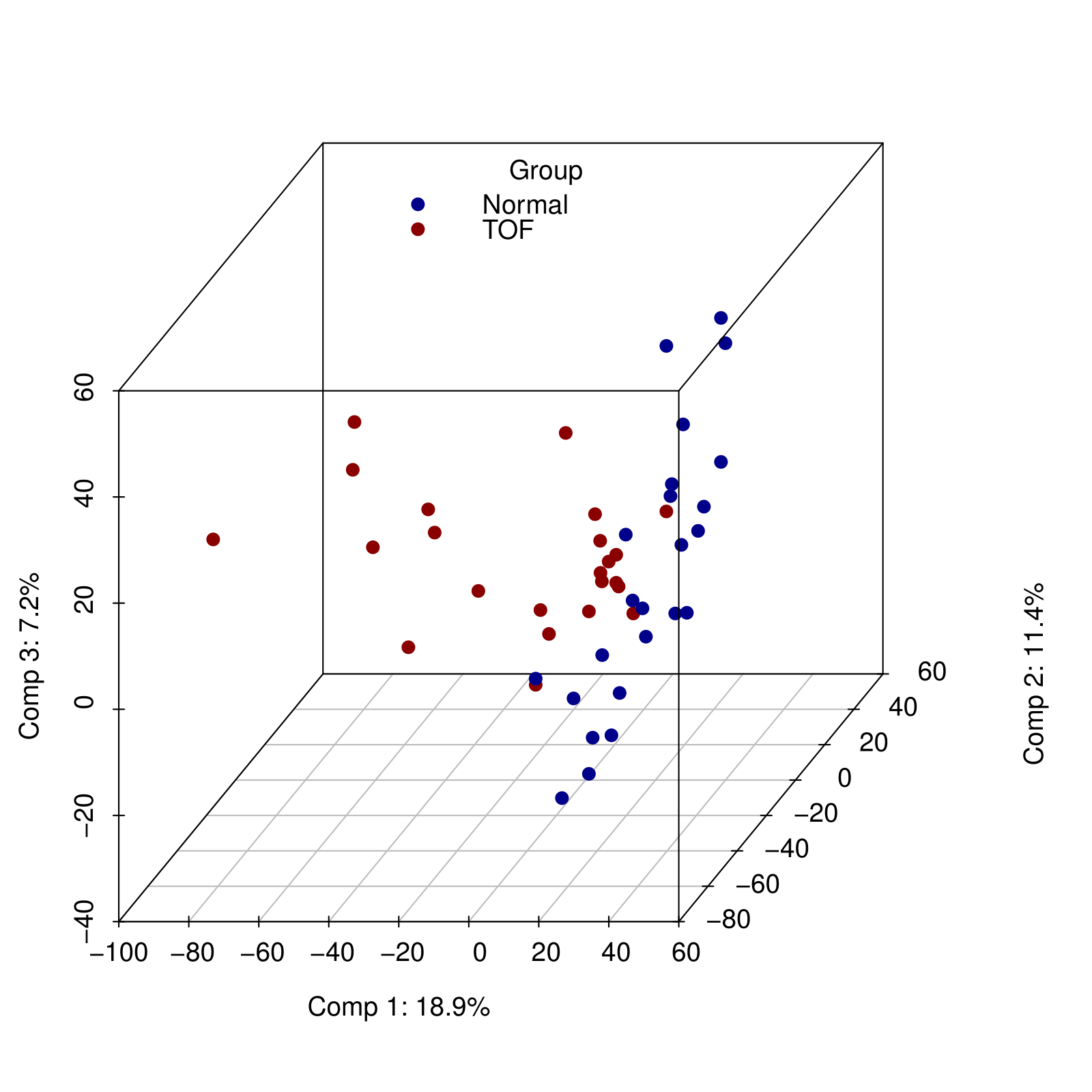

Supplement: S2 Fig — (TIFF) [file pone.0203893.s002.tiff]

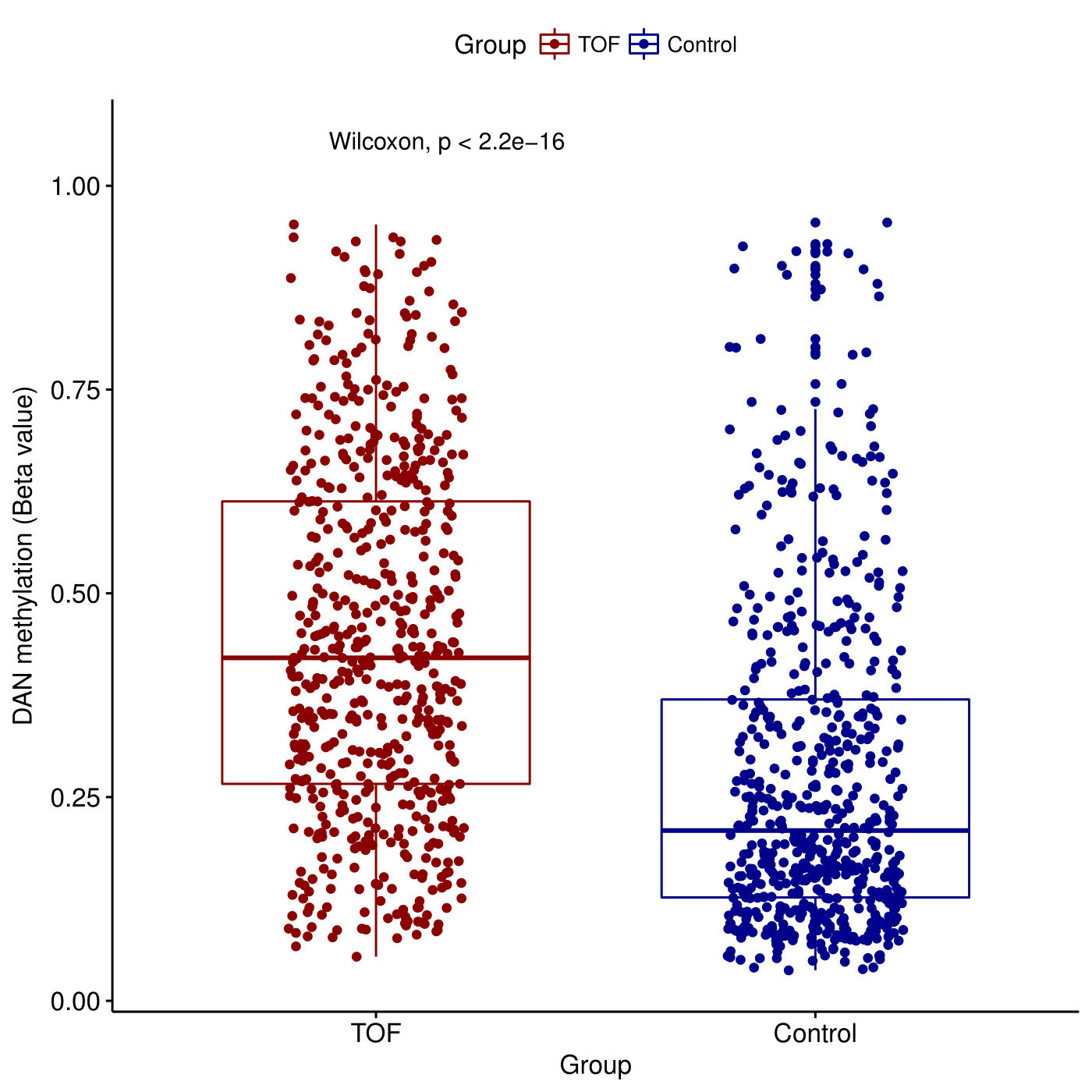

Supplement: S3 Fig — (TIFF) [file pone.0203893.s003.tiff]
