# Supplementary material for: Newborn blood DNA epigenetic variations and signaling pathway genes associated with Tetralogy of Fallot (TOF)
Source: PLoS One. 2018 Sep 13;13(9):e0203893. doi: 10.1371/journal.pone.0203893 (PMC6136787; doi:10.1371/journal.pone.0203893)
Supplement: S1 Table — (PDF) [file pone.0203893.s004.pdf]

TargetID	CHR	Gene	FDR p-Val	Fold change	% Methylation Cases	% Methylation Control	AUC	CI lower	CI upper
cg01311718	2	IKZF2	1.9E-42	2.67	22.71	8.49	0.86	0.74	0.99
cg01400516	16	NETO2	8.038E-05	3.43	5.57	1.62	0.82	0.67	0.97
cg02062326	14	TMED10	1.275E-11	2.19	17.65	8.04	0.87	0.75	1.00
cg02071276	1	FBXO28	1.722E-48	4.08	33.21	8.14	0.87	0.75	1.00
cg02558537	11	CWF19L2	3.31E-12	3.71	11.07	2.99	0.93	0.85	1.00
cg02609279	2	ITGA4	9.762E-44	3.16	24.55	7.76	0.82	0.67	0.97
cg02645710	12	TSPAN19	4.311E-45	2.61	31.31	11.98	0.87	0.74	0.99
cg02981003	10	GPR123	5.504E-12	2.04	20.34	9.97	0.90	0.79	1.00
cg03547245	17	MSI2	5.701E-16	4.80	11.96	2.49	0.84	0.70	0.98
cg03846926	10	C10orf140	1.331E-42	4.26	19.00	4.46	0.98	0.93	1.00
cg04254487	6	TBPL1	3.697E-56	3.62	48.98	13.54	1.00	1.00	1.00
cg04868078	1	LHX9	1.6E-45	3.59	27.86	7.75	0.75	0.57	0.93
cg07002382	15	MFAP1	1.838E-10	3.25	10.69	3.29	0.82	0.67	0.97
cg08264335	1	SELL	4.771E-47	3.56	31.60	8.87	1.00	1.00	1.00
cg08757862	4	TLR1	1.095E-46	3.97	29.59	7.45	0.75	0.57	0.93
cg08821669	12	COX6A1	1.73E-06	0.37	2.78	7.51	0.91	0.81	1.00
cg09365677	1	CHRM3	4.433E-43	3.69	21.27	5.77	0.86	0.73	0.99
cg10225640	12	ANAPC5	2.895E-41	3.33	16.75	5.04	0.84	0.70	0.98
cg10558887	13	SPG20	3.795E-43	4.45	20.16	4.53	0.88	0.75	1.00
cg10944144	5	ADAMTS6	6.784E-43	4.89	19.01	3.89	0.94	0.86	1.00
cg11378242	1	FAM36A	1.442E-41	3.10	18.25	5.88	0.82	0.67	0.97
cg11641791	17	KRT222	1.147E-44	3.51	25.95	7.40	0.78	0.61	0.95
cg11792281	17	NLK	2.16E-30	0.38	11.23	29.53	0.99	0.97	1.00
cg12092090	19	CACNA1A	2.353E-14	2.78	15.82	5.69	0.88	0.75	1.00
cg12273284	10	CAMK1D	5.518E-42	4.69	16.85	3.60	0.98	0.93	1.00
cg12414181	15	SCAMP5	2.993E-30	0.24	4.21	17.87	0.96	0.90	1.00
cg13114458	12	KRR1	2.037E-12	4.02	10.71	2.66	0.93	0.84	1.00
cg14534336	10	JMJD1C	1.58E-13	2.84	14.75	5.18	0.88	0.75	1.00
cg14905634	12	TRHDE	1.721E-28	0.26	10.18	39.08	1.00	1.00	1.00
cg17030055	5	CDH10	5.177E-45	4.06	25.46	6.27	0.88	0.75	1.00
cg17485454	4	MAPK10	1.533E-41	3.33	17.61	5.29	0.99	0.97	1.00
cg17728974	6	LIN28B	3.085E-45	3.33	28.02	8.42	0.88	0.75	1.00
cg18295068	2	SCN3A	1.057E-47	3.42	33.65	9.84	0.77	0.60	0.94
cg18469624	10	PRKG1	5.364E-50	3.50	38.32	10.96	0.94	0.86	1.00
cg18803079	1	EFCAB7	2.387E-30	0.33	10.06	30.88	0.99	0.97	1.00
cg19021985	8	PPP3CC	4.45E-69	4.09	63.07	15.43	1.00	1.00	1.00
cg19533977	17	CLTC	5.394E-43	3.43	21.65	6.32	0.81	0.65	0.96
cg19781109	15	CHD2	2.903E-42	3.08	20.49	6.65	1.00	1.00	1.00
cg20101489	2	SCG2	6.506E-44	4.02	22.79	5.66	0.84	0.69	0.98
cg21364560	10	MYOF	1.929E-57	4.98	46.18	9.28	1.00	1.00	1.00
cg22664298	5	ADAMTS19	7.26E-44	2.40	29.19	12.15	0.85	0.72	0.99
cg23134869	8	ZFHX4	1.735E-10	3.49	10.19	2.92	0.91	0.81	1.00
cg23274377	1	BPNT1	4.162E-14	3.49	12.93	3.70	0.80	0.64	0.96
cg23404012	14	MED6	1.783E-47	3.79	31.86	8.42	0.88	0.75	1.00
cg23680282	12	LRRIQ1	2.926E-43	5.02	19.80	3.94	0.97	0.91	1.00

cg24132989	6	C6orf162	1.066E-26	0.30	16.21	54.01	1.00	1.00	1.00
cg25477497	7	ABCB1	1.756E-45	3.66	27.56	7.53	1.00	1.00	1.00
cg25697769	22	JOSD1	3.312E-09	0.28	2.22	7.89	1.00	1.00	1.00
cg25947619	15	AKAP13	9.682E-43	3.65	20.40	5.58	0.79	0.63	0.96
cg26401673	3	ANO10	3.951E-44	4.43	22.65	5.11	0.88	0.76	1.00
cg26800788	5	PDE4D	1.777E-44	3.88	24.51	6.32	0.86	0.73	0.99
cg27120934	6	LAMA2	5.957E-28	0.27	4.86	18.29	1.00	1.00	1.00
cg27509202	10	CREM	1.62E-42	3.65	19.78	5.42	0.89	0.78	1.00
ch.1.3587792F	1	SMG7	4.202E-49	2.78	40.61	14.59	0.96	0.89	1.00
ch.1.659794R	1	UBR4	5.7E-46	4.88	26.29	5.39	0.90	0.79	1.00
ch.11.319992F	11	USP47	8.87E-49	3.91	34.28	8.76	0.76	0.58	0.94
ch.2.800013F	2	BIRC6	3.241E-42	3.52	19.20	5.46	0.77	0.59	0.94
cg00016718	6	MUT	1.264E-25	0.44	34.80	78.54	1.00	1.00	1.00
cg00027650	15	TCF12	8.525E-26	0.29	17.73	60.49	1.00	1.00	1.00
cg00036369	3	ATXN7	1.474E-25	0.34	22.61	66.74	1.00	1.00	1.00
cg00036599	1	C1orf54	1.453E-25	0.42	31.51	75.60	1.00	1.00	1.00
cg00046623	8	PXDNL	8.109E-26	0.48	39.17	81.80	1.00	1.00	1.00
cg00047079	13	RNF219	6.158E-29	0.48	24.57	51.44	1.00	1.00	1.00
cg00070529	13	ENOX1	9.299E-27	0.48	35.23	72.71	1.00	1.00	1.00
cg00096806	17	TANC2	2.214E-25	0.45	36.83	81.99	1.00	1.00	1.00
cg00134611	6	SNRNP48	1.536E-25	0.48	40.18	84.41	1.00	1.00	1.00
cg00178984	12	SLC16A7	7.744E-27	0.41	26.21	63.28	1.00	1.00	1.00
cg00210098	3	SNORA7A	6.038E-42	3.15	19.30	6.13	1.00	1.00	1.00
cg00218484	4	DDX60L	3.081E-26	0.48	37.40	77.68	1.00	1.00	1.00
cg00223863	3	CEP70	7.89E-25	0.37	28.35	76.83	1.00	1.00	1.00
cg00237586	2	LOC285045	1.034E-26	0.47	32.92	70.65	1.00	1.00	1.00
cg00239074	16	CHD9	5.727E-25	0.45	38.23	85.87	1.00	1.00	1.00
cg00288481	6	TMEM200A	5.591E-26	0.47	37.60	79.33	1.00	1.00	1.00
cg00321074	3	RAB5A	4.68E-24	0.31	23.56	76.97	1.00	1.00	1.00
cg00369142	3	PPP2R3A	4.193E-26	0.46	35.51	76.54	1.00	1.00	1.00
cg00388439	1	PDIA3P	5.129E-25	0.36	26.87	74.21	1.00	1.00	1.00
cg00426855	4	NMU	9.694E-25	0.31	22.13	71.17	1.00	1.00	1.00
cg00471768	20	FLRT3	1.498E-25	0.45	35.66	79.83	1.00	1.00	1.00
cg00530284	6	UTRN	1.149E-25	0.49	41.69	85.19	1.00	1.00	1.00
cg00542384	5	GTF2H2C	2.194E-25	0.45	37.39	82.53	1.00	1.00	1.00
cg00558718	3	HGD	6.364E-56	2.14	65.98	30.82	1.00	1.00	1.00
cg00560724	6	GCNT2	2.61E-26	0.46	33.86	73.75	1.00	1.00	1.00
cg00561338	6	FKSG83	6.478E-26	0.41	29.81	71.89	1.00	1.00	1.00
cg00571870	6	SESN1	1.404E-25	0.47	39.53	83.54	1.00	1.00	1.00
cg00592244	3	SLC25A26	5.799E-26	0.45	33.75	75.57	1.00	1.00	1.00
cg00607609	4	SYNPO2	1.1E-25	0.45	35.57	78.96	1.00	1.00	1.00
cg00611301	6	HDDC2	6.252E-25	0.40	31.49	79.35	1.00	1.00	1.00
cg00616705	2	PSMD1	4.243E-26	0.24	13.17	54.22	1.00	1.00	1.00
cg00655147	10	SLC16A12	1.339E-25	0.42	32.34	76.23	1.00	1.00	1.00
cg00681106	1	LHX8	8.726E-58	2.78	58.20	20.93	1.00	1.00	1.00
cg00707452	3	FOXP1	1.982E-10	2.19	16.16	7.38	1.00	1.00	1.00
cg00710736	3	ZNF148	7.764E-27	0.46	32.03	69.10	1.00	1.00	1.00
cg00735454	13	LOC121838	7.191E-27	0.45	30.56	67.46	1.00	1.00	1.00

cg00754027	1	KIF14	1.902E-48	2.87	38.38	13.37	1.00	1.00	1.00
cg00762705	2	CRIM1	3.377E-26	0.44	32.26	72.77	1.00	1.00	1.00
cg00765144	10	MLLT10	2.256E-26	0.49	37.62	77.16	1.00	1.00	1.00
cg00779294	3	POU1F1	5.381E-25	0.37	27.29	74.76	1.00	1.00	1.00
cg00789416	14	SNORD113-5	1.781E-26	0.48	36.17	75.16	1.00	1.00	1.00
cg00789793	3	RASA2	1.491E-26	0.44	29.80	68.37	1.00	1.00	1.00
cg00817501	4	PCDH18	6.701E-26	0.43	31.54	73.71	1.00	1.00	1.00
cg00844078	2	ASXL2	3.033E-25	0.35	25.29	71.26	1.00	1.00	1.00
cg00847250	15	GPR176	6.356E-27	0.48	33.95	70.57	1.00	1.00	1.00
cg00894559	18	IMPACT	1.137E-25	0.49	41.00	84.48	1.00	1.00	1.00
cg00916117	6	ZNF451	5.023E-25	0.41	33.25	80.53	1.00	1.00	1.00
cg00938877	1	OR2L2	4.025E-26	0.43	30.39	71.32	1.00	1.00	1.00
cg00973397	15	ATP8B4	3.908E-25	0.43	35.69	82.32	1.00	1.00	1.00
cg01013868	14	SNORD114-23	5.778E-25	0.40	32.28	79.93	1.00	1.00	1.00
cg01043759	7	CALD1	3.097E-26	0.49	38.92	79.22	1.00	1.00	1.00
cg01063615	17	TAF15	9.617E-27	0.46	32.61	70.18	1.00	1.00	1.00
cg01064286	1	ATF3	5.329E-27	0.48	33.39	69.61	1.00	1.00	1.00
cg01066189	4	LOC340017	2.859E-28	0.47	26.59	56.52	1.00	1.00	1.00
cg01201151	2	MARS2	2.614E-25	0.38	27.91	73.49	1.00	1.00	1.00
cg01239717	6	RTN4IP1	1.111E-08	0.32	2.62	8.23	1.00	1.00	1.00
cg01289318	3	TRPC1	7.372E-26	0.49	41.17	83.57	1.00	1.00	1.00
cg01293179	2	HOXD8	1.835E-49	2.50	44.25	17.69	1.00	1.00	1.00
cg01310768	5	LYSMD3	7.33E-28	0.49	31.03	62.92	1.00	1.00	1.00
cg01347798	6	MCM3	5.287E-27	0.48	33.27	69.47	1.00	1.00	1.00
cg01363648	1	PTPRU	3.209E-27	0.47	31.21	66.30	1.00	1.00	1.00
cg01364769	10	COMMD3	8.504E-27	0.48	33.93	71.21	1.00	1.00	1.00
cg01367751	7	KCND2	1.051E-22	0.19	14.86	77.70	1.00	1.00	1.00
cg01401015	5	PCDHB11	6.783E-46	2.31	36.65	15.88	1.00	1.00	1.00
cg01410314	9	C9orf41	2.588E-26	0.50	39.48	79.35	1.00	1.00	1.00
cg01417339	8	MIR30B	2.588E-25	0.44	35.74	81.30	1.00	1.00	1.00
cg01455446	4	ELF2	3.483E-26	0.46	33.91	74.49	1.00	1.00	1.00
cg01513516	2	TMEM163	3.356E-27	0.47	30.85	66.03	1.00	1.00	1.00
cg01554140	14	C14orf129	2.741E-25	0.37	27.10	72.81	1.00	1.00	1.00
cg01555907	9	NDUFB6	1.791E-25	0.44	35.43	80.05	1.00	1.00	1.00
cg01576496	12	PPP1R12A	1.341E-24	0.37	29.66	79.57	1.00	1.00	1.00
cg01592108	2	CHN1	4.129E-25	0.42	33.34	80.12	1.00	1.00	1.00
cg01614536	7	PON2	1.547E-26	0.41	27.34	66.00	1.00	1.00	1.00
cg01643441	14	MAX	8.889E-47	2.86	34.30	12.01	1.00	1.00	1.00
cg01645995	7	HEPACAM2	2.513E-25	0.41	31.76	77.25	1.00	1.00	1.00
cg01683756	6	C6orf163	2.474E-29	0.47	22.26	47.36	1.00	1.00	1.00
cg01693326	11	CHORDC1	4.82E-26	0.47	36.66	78.03	1.00	1.00	1.00
cg01698567	14	SFTA3	6.735E-26	0.44	33.71	75.89	1.00	1.00	1.00
cg01704666	8	ZFPM2	8.727E-26	0.47	37.42	80.24	1.00	1.00	1.00
cg01760846	14	PSMC1	6.733E-46	2.04	40.67	19.90	1.00	1.00	1.00
cg01774111	1	ANGEL2	4.96E-26	0.49	39.20	80.64	1.00	1.00	1.00
cg01827202	1	DNAJC6	8.69E-26	0.46	37.09	79.89	1.00	1.00	1.00
cg01868347	2	HNRPLL	8.974E-26	0.49	42.03	84.92	1.00	1.00	1.00
cg01895014	14	FAM164C	1.253E-25	0.47	38.86	82.58	1.00	1.00	1.00

cg01924320	4	SLC10A7	6.343E-26	0.44	32.55	74.58	1.00	1.00	1.00
cg02023912	6	FAM135A	1.125E-25	0.43	32.18	75.63	1.00	1.00	1.00
cg02042904	12	LRRK2	9.819E-24	0.32	25.79	81.34	1.00	1.00	1.00
cg02047170	5	TNPO1	1.455E-24	0.33	25.18	75.32	1.00	1.00	1.00
cg02065293	2	MYT1L	1.783E-26	0.49	37.32	76.31	1.00	1.00	1.00
cg02086174	14	C14orf143	8.143E-28	0.47	18.16	38.75	1.00	1.00	1.00
cg02135728	3	ARL6	1.537E-26	0.49	37.16	75.81	1.00	1.00	1.00
cg02246683	8	NCOA2	1.628E-27	0.41	23.40	56.99	1.00	1.00	1.00
cg02259429	14	KIAA1409	2.276E-25	0.44	35.17	80.41	1.00	1.00	1.00
cg02317746	3	SLC9A9	3.298E-26	0.47	35.84	76.29	1.00	1.00	1.00
cg02322135	4	ANK2	2.54E-24	0.37	29.90	81.58	1.00	1.00	1.00
cg02335863	17	CDC27	1.231E-26	0.49	36.29	74.42	1.00	1.00	1.00
cg02344001	7	SNX13	7.224E-27	0.39	23.67	60.57	1.00	1.00	1.00
cg02362505	1	HMCN1	3.131E-25	0.41	32.05	78.10	1.00	1.00	1.00
cg02362508	6	NRSN1	2.55E-25	0.39	29.56	75.08	1.00	1.00	1.00
cg02363960	7	MAGI2	3.108E-28	0.50	29.75	59.85	1.00	1.00	1.00
cg02393449	10	DDX21	2.689E-26	0.50	39.48	79.44	1.00	1.00	1.00
cg02435427	10	AKR1C4	2.521E-26	0.47	35.39	75.20	1.00	1.00	1.00
cg02447227	1	NFIA	1.724E-26	0.47	34.93	73.84	1.00	1.00	1.00
cg02546477	12	ERP27	1.657E-26	0.35	20.60	59.42	1.00	1.00	1.00
cg02570644	16	NFATC3	1.639E-26	0.48	35.72	74.52	1.00	1.00	1.00
cg02580987	5	NSD1	1.539E-24	0.38	30.58	80.88	1.00	1.00	1.00
cg02593601	10	STAM	3.135E-26	0.47	35.54	75.87	1.00	1.00	1.00
cg02602268	12	EP400	2.915E-24	0.35	27.84	79.90	1.00	1.00	1.00
cg02623718	1	FMN2	3.462E-26	0.45	33.78	74.35	1.00	1.00	1.00
cg02638909	8	PURG	1.317E-25	0.44	34.69	78.54	1.00	1.00	1.00
cg02655623	17	C17orf85	1.035E-25	0.41	30.56	73.80	1.00	1.00	1.00
cg02656851	11	SOX6	1.539E-26	0.45	31.52	70.16	1.00	1.00	1.00
cg02672232	1	XPR1	2.215E-27	0.49	33.28	67.55	1.00	1.00	1.00
cg02739437	4	SPOCK3	5.588E-28	0.47	27.30	58.63	1.00	1.00	1.00
cg02741305	5	CCDC152	4.924E-26	0.49	40.42	81.83	1.00	1.00	1.00
cg02743252	10	VCL	1.064E-25	0.48	40.45	83.76	1.00	1.00	1.00
cg02766539	17	TMEM49	1.12E-26	0.49	36.20	74.11	1.00	1.00	1.00
cg02833117	2	ERMN	1.439E-25	0.47	39.34	83.41	1.00	1.00	1.00
cg02848106	3	SKIL	1.004E-26	0.48	35.18	72.84	1.00	1.00	1.00
cg02857312	2	BRE	1.685E-27	0.49	32.98	66.66	1.00	1.00	1.00
cg02877657	2	MTIF2	2.597E-28	0.41	20.49	50.23	1.00	1.00	1.00
cg02977388	10	RNLS	1.568E-26	0.42	28.07	66.76	1.00	1.00	1.00
cg02990507	14	SNORD113-9	1.605E-25	0.45	36.43	80.77	1.00	1.00	1.00
cg03015358	10	PDZD8	2.373E-25	0.44	35.15	80.49	1.00	1.00	1.00
cg03022926	14	TC2N	2.883E-27	0.50	34.28	69.13	1.00	1.00	1.00
cg03038520	16	CRYM	1.365E-25	0.41	30.74	74.67	1.00	1.00	1.00
cg03045067	16	ZFHX3	4.009E-25	0.40	31.47	78.16	1.00	1.00	1.00
cg03064793	4	FAT1	9.478E-27	0.50	37.51	75.04	1.00	1.00	1.00
cg03077969	6	NFKBIL1	1.171E-25	0.45	35.50	79.05	1.00	1.00	1.00
cg03084808	16	NFAT5	2.801E-26	0.49	39.07	79.13	1.00	1.00	1.00
cg03099832	4	IL15	1.444E-26	0.46	32.53	71.03	1.00	1.00	1.00
cg03110165	13	GPC6	1.056E-25	0.39	28.12	71.42	1.00	1.00	1.00

cg03126616	5	RUFY1	5.347E-26	0.49	39.74	81.36	1.00	1.00	1.00
cg03145199	7	DNAJC2	1.041E-26	0.48	35.09	72.83	1.00	1.00	1.00
cg03177593	3	FNDC3B	1.131E-24	0.36	27.54	77.00	1.00	1.00	1.00
cg03181618	2	WDSUB1	4.56E-26	0.48	38.43	79.66	1.00	1.00	1.00
cg03261347	6	C6orf97	1.139E-25	0.45	34.89	78.37	1.00	1.00	1.00
cg03267502	4	NEK1	1.693E-43	2.34	28.53	12.21	1.00	1.00	1.00
cg03272195	17	HS3ST3B1	7.476E-26	0.50	41.71	84.15	1.00	1.00	1.00
cg03287579	10	NEBL	1.113E-26	0.48	35.51	73.40	1.00	1.00	1.00
cg03293350	18	TCF4	1.833E-25	0.44	34.47	79.15	1.00	1.00	1.00
cg03319695	18	CDH20	2.485E-27	0.49	32.96	67.48	1.00	1.00	1.00
cg03338527	7	EXOC4	7.894E-26	0.49	40.19	82.76	1.00	1.00	1.00
cg03407996	5	MIR103-1	3.958E-27	0.42	26.28	61.83	1.00	1.00	1.00
cg03421069	13	PAN3	1.925E-29	0.43	18.22	42.86	1.00	1.00	1.00
cg03451959	3	PROS1	2.801E-26	0.42	28.94	69.00	1.00	1.00	1.00
cg03459617	4	DSPP	5.066E-27	0.50	35.77	71.88	1.00	1.00	1.00
cg03460032	13	RB1	6.628E-24	0.30	22.90	77.31	1.00	1.00	1.00
cg03473016	3	MYLK	1.471E-25	0.45	36.27	80.39	1.00	1.00	1.00
cg03492327	14	OTX2	8.527E-48	2.37	41.44	17.47	1.00	1.00	1.00
cg03503288	13	ZMYM2	1.685E-25	0.44	35.00	79.47	1.00	1.00	1.00
cg03535648	12	PMCH	1.024E-24	0.41	33.53	82.71	1.00	1.00	1.00
cg03539313	3	MFSD1	3.153E-26	0.45	33.43	73.77	1.00	1.00	1.00
cg03546163	6	FKBP5	4.271E-25	0.32	21.73	68.59	1.00	1.00	1.00
cg03555067	5	RICTOR	7.504E-26	0.38	25.64	68.08	1.00	1.00	1.00
cg03563169	6	TBX18	1.186E-26	0.35	20.93	58.98	1.00	1.00	1.00
cg03596021	7	DUS4L	1.162E-24	0.31	22.33	71.86	1.00	1.00	1.00
cg03607891	1	LEPR	1.025E-26	0.49	35.70	73.41	1.00	1.00	1.00
cg03623568	16	A2BP1	1.408E-26	0.49	36.95	75.39	1.00	1.00	1.00
cg03649429	5	COL4A3BP	1.091E-26	0.48	34.83	72.68	1.00	1.00	1.00
cg03658604	13	DIAPH3	1.115E-25	0.49	41.02	84.45	1.00	1.00	1.00
cg03666597	7	CUX1	2.98E-27	0.40	23.62	58.54	1.00	1.00	1.00
cg03678722	2	AGFG1	8.659E-25	0.35	26.33	75.06	1.00	1.00	1.00
cg03689601	12	PUS7L	2.019E-26	0.47	34.41	73.70	1.00	1.00	1.00
cg03716388	7	MDFIC	1.432E-26	0.46	33.44	71.93	1.00	1.00	1.00
cg03729941	3	NLGN1	1.161E-51	2.16	55.40	25.67	1.00	1.00	1.00
cg03752430	20	MACROD2	8.399E-27	0.48	34.83	72.08	1.00	1.00	1.00
cg03759239	1	RGS13	7.641E-26	0.44	33.64	76.13	1.00	1.00	1.00
cg03797139	6	HECA	1.966E-25	0.46	38.71	83.57	1.00	1.00	1.00
cg03870411	4	C4orf32	3.007E-26	0.44	31.42	71.65	1.00	1.00	1.00
cg03893339	3	NPHP3	3.596E-25	0.41	32.51	78.92	1.00	1.00	1.00
cg03910557	6	HEY2	2.68E-26	0.47	35.20	75.16	1.00	1.00	1.00
cg03914237	7	THSD7A	1.842E-26	0.46	33.90	72.97	1.00	1.00	1.00
cg03914913	7	KLF14	1.75E-53	2.21	58.69	26.52	1.00	1.00	1.00
cg03915558	2	OSBPL6	2.111E-25	0.43	34.27	79.31	1.00	1.00	1.00
cg03951121	21	MRAP	1.216E-27	0.48	30.99	63.96	1.00	1.00	1.00
cg03951662	17	CPD	5.147E-25	0.38	29.34	76.69	1.00	1.00	1.00
cg03967533	1	KDM1A	6.416E-26	0.49	39.89	81.95	1.00	1.00	1.00
cg03971247	2	PIGF	1.636E-24	0.33	24.41	74.88	1.00	1.00	1.00
cg03982568	5	KCTD16	1.347E-25	0.40	29.72	73.62	1.00	1.00	1.00

cg04038724	7	SRI	1.797E-25	0.39	29.02	73.65	1.00	1.00	1.00
cg04104489	1	PGM1	8.308E-28	0.50	31.70	63.86	1.00	1.00	1.00
cg04112058	16	SHCBP1	1.711E-25	0.40	30.24	74.74	1.00	1.00	1.00
cg04118234	7	ZC3HAV1	4.724E-61	2.99	61.15	20.48	1.00	1.00	1.00
cg04133673	2	LRP1B	1.661E-26	0.45	31.39	70.22	1.00	1.00	1.00
cg04162118	8	TRAM1	1.735E-26	0.47	34.25	73.17	1.00	1.00	1.00
cg04175877	5	ERBB2IP	4.049E-26	0.49	39.57	80.52	1.00	1.00	1.00
cg04250181	12	SLCO1B3	5.567E-27	0.23	11.08	47.40	1.00	1.00	1.00
cg04297001	10	ATAD1	5.959E-25	0.38	29.52	77.25	1.00	1.00	1.00
cg04297867	15	USP3	8.552E-26	0.49	40.57	83.34	1.00	1.00	1.00
cg04312413	1	TPR	6.94E-25	0.40	31.84	79.98	1.00	1.00	1.00
cg04319742	17	KRTAP4-1	1.078E-25	0.44	33.96	77.30	1.00	1.00	1.00
cg04334979	4	BMP2K	4.337E-25	0.40	31.42	78.32	1.00	1.00	1.00
cg04343242	4	BDH2	3.456E-27	0.37	20.77	56.02	1.00	1.00	1.00
cg04364339	10	IFIT3	2.098E-44	2.45	30.52	12.46	1.00	1.00	1.00
cg04367614	6	SPACA1	2.515E-26	0.50	39.66	79.46	1.00	1.00	1.00
cg04395431	14	FANCM	1.586E-26	0.48	35.26	73.98	1.00	1.00	1.00
cg04407530	8	RIMS2	3.998E-25	0.31	21.20	67.89	1.00	1.00	1.00
cg04420991	4	NUP54	7.523E-26	0.47	37.56	80.01	1.00	1.00	1.00
cg04474257	4	SLC7A11	6.055E-24	0.29	22.54	76.68	1.00	1.00	1.00
cg04483101	3	TP63	8.89E-27	0.49	35.24	72.62	1.00	1.00	1.00
cg04483989	4	C4orf22	4.743E-27	0.47	32.23	68.19	1.00	1.00	1.00
cg04531010	6	PHF3	1.97E-25	0.39	28.15	73.02	1.00	1.00	1.00
cg04564333	1	PPPDE1	3.283E-26	0.50	40.31	80.75	1.00	1.00	1.00
cg04583467	12	CNOT2	3.229E-26	0.39	25.30	65.69	1.00	1.00	1.00
cg04623023	7	LOC100124692	1.439E-26	0.48	36.17	74.66	1.00	1.00	1.00
cg04657419	5	C5orf43	8.981E-27	0.48	34.78	72.19	1.00	1.00	1.00
cg04663692	12	RMST;MIR1251	9.224E-27	0.48	33.98	71.45	1.00	1.00	1.00
cg04686481	2	PDE11A	3.521E-26	0.45	33.21	73.82	1.00	1.00	1.00
cg04703974	17	SYNRG	9.736E-26	0.49	40.77	83.86	1.00	1.00	1.00
cg04732596	2	MEIS1	2.259E-26	0.44	30.47	70.02	1.00	1.00	1.00
cg04779597	3	SELT	6.612E-26	0.45	34.73	76.87	1.00	1.00	1.00
cg04813693	6	RIMS1	5.026E-27	0.48	32.74	68.82	1.00	1.00	1.00
cg04857881	16	ITFG1	8.699E-27	0.50	36.98	74.31	1.00	1.00	1.00
cg04901812	10	SH2D4B	1.785E-44	2.05	35.58	17.39	1.00	1.00	1.00
cg04928692	2	ATP5G3	3.009E-25	0.45	37.09	83.04	1.00	1.00	1.00
cg04935109	3	RTP4	4.013E-10	2.06	17.36	8.44	1.00	1.00	1.00
cg05016201	10	SHOC2	1.995E-26	0.47	35.35	74.61	1.00	1.00	1.00
cg05046717	3	OSBPL11	1.155E-25	0.48	39.53	83.05	1.00	1.00	1.00
cg05048730	11	OR5B12	1.078E-25	0.41	30.03	73.37	1.00	1.00	1.00
cg05118879	9	RFX3;RFX3	1.218E-50	3.21	41.10	12.80	1.00	1.00	1.00
cg05132905	2	ATL2	1.072E-25	0.49	40.99	84.32	1.00	1.00	1.00
cg05162900	17	GOSR1	2.308E-25	0.46	39.08	84.34	1.00	1.00	1.00
cg05225993	7	EZH2	2.806E-25	0.41	31.83	77.60	1.00	1.00	1.00
cg05266796	6	ATXN1	4.771E-24	0.37	31.17	84.63	1.00	1.00	1.00
cg05335893	2	THADA	5.527E-27	0.40	24.69	60.99	1.00	1.00	1.00
cg05338527	12	PDZRN4	5.989E-25	0.37	27.70	75.45	1.00	1.00	1.00
cg05368119	1	S100A10	9.548E-27	0.43	28.51	66.05	1.00	1.00	1.00

cg05385567	16	KCTD13	3.627E-16	2.29	21.90	9.57	1.00	1.00	1.00
cg05390563	1	MTF2	1.812E-47	3.45	32.99	9.55	1.00	1.00	1.00
cg05408873	6	COL10A1;NT5DC1	2.022E-26	0.46	33.75	73.04	1.00	1.00	1.00
cg05412784	8	CPA6	9.819E-26	0.46	36.49	79.60	1.00	1.00	1.00
cg05466223	11	PIK3C2A	4.595E-25	0.41	32.92	79.97	1.00	1.00	1.00
cg05553474	12	NDUFA9	4.987E-13	2.21	19.23	8.70	1.00	1.00	1.00
cg05558399	11	CASP4	5.116E-26	0.47	37.51	79.02	1.00	1.00	1.00
cg05572930	6	RNF217	6.169E-27	0.45	29.99	66.54	1.00	1.00	1.00
cg05575228	5	ST8SIA4	1.646E-23	0.31	25.29	82.38	1.00	1.00	1.00
cg05577848	12	USP15	3.264E-25	0.43	34.66	80.82	1.00	1.00	1.00
cg05584737	18	LMAN1	7.774E-26	0.43	32.24	74.78	1.00	1.00	1.00
cg05609408	2	ANAPC1	3.886E-52	2.08	58.45	28.06	1.00	1.00	1.00
cg05687370	4	AP1AR	5.707E-26	0.47	36.97	78.75	1.00	1.00	1.00
cg05740739	2	OR6B3	8.038E-27	0.46	31.62	68.77	1.00	1.00	1.00
cg05750323	6	SYNE1	7.902E-27	0.42	26.54	63.65	1.00	1.00	1.00
cg05762338	17	KRTAP1-5	4.729E-25	0.45	37.84	84.96	1.00	1.00	1.00
cg05789658	1	ATP6V1G3	5.602E-27	0.48	33.85	70.18	1.00	1.00	1.00
cg05858008	14	FRMD6	4.119E-26	0.39	26.01	66.99	1.00	1.00	1.00
cg05874309	15	CPEB1	1.186E-25	0.47	39.15	82.74	1.00	1.00	1.00
cg05877590	10	OR13A1	9.699E-26	0.42	31.66	74.74	1.00	1.00	1.00
cg05882781	10	ARID5B	5.659E-50	3.45	38.47	11.14	1.00	1.00	1.00
cg05889123	6	CD2AP	1.298E-25	0.45	35.73	79.54	1.00	1.00	1.00
cg05895410	7	C7orf10	6.793E-25	0.37	27.74	75.82	1.00	1.00	1.00
cg05917188	7	CD36	7.302E-26	0.37	24.85	67.23	1.00	1.00	1.00
cg05962710	7	LAMB4	4.508E-25	0.42	34.65	81.65	1.00	1.00	1.00
cg05998244	2	LONRF2	6.105E-23	0.24	19.01	80.12	1.00	1.00	1.00
cg06013872	4	SLC4A4	6.62E-27	0.41	25.52	62.23	1.00	1.00	1.00
cg06026425	5	CLINT1	3.156E-50	3.04	41.27	13.57	1.00	1.00	1.00
cg06056880	5	GABRB2	6.213E-28	0.44	24.67	56.22	1.00	1.00	1.00
cg06057666	1	CDC42BPA	2.913E-26	0.44	30.93	71.09	1.00	1.00	1.00
cg06081147	7	AASS	9.85E-25	0.33	23.94	73.02	1.00	1.00	1.00
cg06083252	10	RUFY2	6.077E-26	0.38	26.08	68.01	1.00	1.00	1.00
cg06293745	7	RUNDC3B;ABCB1	3.199E-23	0.29	23.86	82.96	1.00	1.00	1.00
cg06295535	4	PPP3CA	6.023E-25	0.44	37.54	85.31	1.00	1.00	1.00
cg06352809	10	PLEKHA1	3.769E-26	0.49	39.32	80.09	1.00	1.00	1.00
cg06370862	6	TFAP2D	1.429E-45	2.38	34.81	14.62	1.00	1.00	1.00
cg06375876	4	CNOT6L	2.141E-26	0.49	37.56	76.98	1.00	1.00	1.00
cg06379340	2	UNC80	5.839E-27	0.49	34.34	70.76	1.00	1.00	1.00
cg06382770	12	VEZT	1.166E-25	0.41	30.27	73.81	1.00	1.00	1.00
cg06415087	7	ARL4A	4.662E-15	0.42	7.25	17.43	1.00	1.00	1.00
cg06424110	10	BICC1	2.194E-24	0.32	24.56	75.84	1.00	1.00	1.00
cg06470626	16	CDR2	2.584E-43	2.21	29.18	13.22	1.00	1.00	1.00
cg06539434	6	ASCC3	1.093E-24	0.37	28.87	78.23	1.00	1.00	1.00
cg06579338	16	ERI2	7.37E-26	0.48	39.05	81.46	1.00	1.00	1.00
cg06627041	11	OPCML	6.501E-27	0.49	35.11	71.78	1.00	1.00	1.00
cg06665095	4	RXFP1	4.806E-25	0.43	35.26	82.43	1.00	1.00	1.00
cg06686857	8	C8orf39;RBM12B	2.658E-27	0.50	34.48	69.15	1.00	1.00	1.00
cg06712285	3	RBMS3	2.137E-24	0.38	31.07	82.27	1.00	1.00	1.00

cg06740311	18	CEP76	1.372E-23	0.27	20.39	76.93	1.00	1.00	1.00
cg06748934	4	KIAA1712	1.45E-25	0.49	42.23	86.31	1.00	1.00	1.00
cg06758980	2	MBD5	1.918E-23	0.28	22.83	80.37	1.00	1.00	1.00
cg06798189	3	SLC4A7	1.762E-26	0.48	36.22	75.19	1.00	1.00	1.00
cg06817312	3	EXOG	2.691E-26	0.43	30.12	70.09	1.00	1.00	1.00
cg06870124	11	OR52K1	8.852E-26	0.46	36.96	79.82	1.00	1.00	1.00
cg06912824	11	RPUSD4	7.313E-27	0.49	35.13	72.06	1.00	1.00	1.00
cg06995966	5	MYOT	5.971E-28	0.49	30.17	61.64	1.00	1.00	1.00
cg07008343	17	KRT28	1.293E-27	0.45	27.09	60.19	1.00	1.00	1.00
cg07012076	11	OR5AS1	2.337E-25	0.41	31.85	77.15	1.00	1.00	1.00
cg07015465	5	RARS	1.315E-27	0.47	28.92	62.05	1.00	1.00	1.00
cg07040517	7	THAP5	9.928E-26	0.48	40.15	83.29	1.00	1.00	1.00
cg07069087	4	GRID2	3.482E-25	0.42	33.09	79.41	1.00	1.00	1.00
cg07076091	4	SC4MOL	3.611E-25	0.41	32.63	79.05	1.00	1.00	1.00
cg07144666	7	KDELR2	7.771E-25	0.44	37.79	86.24	1.00	1.00	1.00
cg07152379	15	WDR76	2.848E-25	0.41	31.29	77.10	1.00	1.00	1.00
cg07179075	1	DPYD	6.259E-26	0.45	34.04	76.05	1.00	1.00	1.00
cg07192594	16	HYDIN	6.869E-28	0.44	25.30	57.06	1.00	1.00	1.00
cg07215003	5	PPP2CA	1.944E-25	0.43	33.31	78.14	1.00	1.00	1.00
cg07296661	2	SCN9A	1.351E-25	0.48	40.30	84.21	1.00	1.00	1.00
cg07309576	11	OR51I1	2.068E-26	0.47	35.30	74.64	1.00	1.00	1.00
cg07367144	7	MLL3	2.333E-25	0.46	39.30	84.60	1.00	1.00	1.00
cg07462863	17	EVI2A;NF1	1.394E-26	0.45	31.97	70.39	1.00	1.00	1.00
cg07585876	8	ZFHX4; LOC100192378	1.754E-45	2.07	38.75	18.72	1.00	1.00	1.00
cg07594613	4	TBC1D19	4.015E-26	0.46	34.61	75.53	1.00	1.00	1.00
cg07714266	7	JAZF1	8.003E-28	0.40	21.68	53.76	1.00	1.00	1.00
cg07759052	8	HMBOX1	1.041E-25	0.49	41.06	84.31	1.00	1.00	1.00
cg07797370	12	TMEM117	3.733E-26	0.45	33.12	73.87	1.00	1.00	1.00
cg07810677	4	COL25A1	3.253E-26	0.44	32.20	72.62	1.00	1.00	1.00
cg07817886	16	DNAJA2	4.585E-26	0.49	39.49	80.73	1.00	1.00	1.00
cg07824897	6	DST	2.426E-25	0.41	32.06	77.45	1.00	1.00	1.00
cg07829809	4	METAP1	4.415E-26	0.42	30.29	71.44	1.00	1.00	1.00
cg07901130	6	ELOVL2	2.411E-26	0.48	36.70	76.41	1.00	1.00	1.00
cg07919753	12	ZNF26	3.001E-26	0.47	36.05	76.28	1.00	1.00	1.00
cg07922204	17	EFCAB5	4.756E-28	0.49	30.20	61.18	1.00	1.00	1.00
cg07926074	7	TMEM168	1.582E-25	0.40	30.13	74.44	1.00	1.00	1.00
cg08059402	3	ATP13A3	1.355E-24	0.36	27.90	77.84	1.00	1.00	1.00
cg08069338	6	SNORA29;TCP1	9.164E-26	0.46	36.04	78.98	1.00	1.00	1.00
cg08096038	6	SFRS18	1.752E-25	0.37	26.10	70.66	1.00	1.00	1.00
cg08099115	3	ERC2	6.493E-29	0.47	24.35	51.31	1.00	1.00	1.00
cg08123413	7	LRRC17;FBXL13	1.459E-25	0.33	21.54	65.64	1.00	1.00	1.00
cg08125824	14	HECTD1	2.375E-24	0.37	30.53	82.03	1.00	1.00	1.00
cg08126158	2	DUSP19	2.4E-26	0.47	34.76	74.45	1.00	1.00	1.00
cg08135437	2	PSMD14	2.374E-25	0.39	28.88	74.22	1.00	1.00	1.00
cg08145262	12	FRS2	2.08E-25	0.42	32.42	77.42	1.00	1.00	1.00
cg08166758	2	ARHGAP25	1.207E-24	0.37	29.05	78.68	1.00	1.00	1.00
cg08190562	6	UBD	1.361E-26	0.48	35.77	74.14	1.00	1.00	1.00

cg08285589	11	RAG1	1.14E-26	0.47	33.73	71.68	1.00	1.00	1.00
cg08289189	22	COMT	1.721E-26	0.50	38.35	77.26	1.00	1.00	1.00
cg08345065	10	CPEB3	1.711E-26	0.49	37.48	76.38	1.00	1.00	1.00
cg08365638	16	C16orf72	2.599E-26	0.49	37.65	77.53	1.00	1.00	1.00
cg08375941	3	GPR15	4.399E-26	0.49	40.14	81.29	1.00	1.00	1.00
cg08385173	17	C17orf80;CPSF4L	3.041E-27	0.36	19.46	54.43	1.00	1.00	1.00
cg08422456	4	IBSP	6.295E-26	0.42	30.18	72.19	1.00	1.00	1.00
cg08530065	13	LHFP	5.356E-26	0.40	27.63	69.25	1.00	1.00	1.00
cg08610281	1	DTL	2.911E-25	0.41	32.17	78.04	1.00	1.00	1.00
cg08612570	7	TAX1BP1	5.277E-26	0.43	31.98	73.56	1.00	1.00	1.00
cg08776820	5	PPAP2A;SKIV2L2	4.805E-26	0.46	34.60	75.96	1.00	1.00	1.00
cg08851840	10	TCF7L2	2.089E-10	2.10	17.23	8.21	1.00	1.00	1.00
cg08858272	13	NALCN	1.3E-27	0.43	25.09	58.20	1.00	1.00	1.00
cg08885076	2	TSGA10	7.949E-28	0.50	31.80	63.86	1.00	1.00	1.00
cg08972357	5	C5orf37	1.418E-26	0.49	37.43	75.89	1.00	1.00	1.00
cg08996334	3	ZBTB20	1.909E-26	0.48	35.44	74.60	1.00	1.00	1.00
cg09049417	8	FZD3	1.458E-26	0.50	38.13	76.65	1.00	1.00	1.00
cg09096383	4	CSN1S1	8.865E-25	0.35	25.92	74.72	1.00	1.00	1.00
cg09132121	2	RBM45	5.939E-25	0.41	33.32	81.05	1.00	1.00	1.00
cg09238520	4	INPP4B	3.858E-26	0.46	35.47	76.30	1.00	1.00	1.00
cg09261289	12	KLRD1	4.539E-26	0.44	33.05	74.26	1.00	1.00	1.00
cg09287295	19	ZNF470	4.586E-26	0.45	33.81	75.05	1.00	1.00	1.00
cg09321620	14	JKAMP	1.234E-25	0.46	36.86	80.55	1.00	1.00	1.00
cg09379340	6	TIAM2	3.206E-45	2.56	32.11	12.55	1.00	1.00	1.00
cg09408366	1	TTLL7	1.866E-26	0.44	31.02	70.12	1.00	1.00	1.00
cg09526693	4	IL2	1.742E-24	0.34	26.08	76.71	1.00	1.00	1.00
cg09537568	4	WDFY3	3.622E-24	0.33	26.02	78.69	1.00	1.00	1.00
cg09538031	5	HEATR7B2	8.328E-25	0.35	26.44	75.07	1.00	1.00	1.00
cg09554701	1	ACBD6	4.063E-25	0.41	33.01	79.74	1.00	1.00	1.00
cg09600247	12	PRR4;PRH1; TAS2R50	7.483E-26	0.45	35.34	77.78	1.00	1.00	1.00
cg09659223	21	KRTAP20-4	2.303E-26	0.49	37.76	77.35	1.00	1.00	1.00
cg09681278	4	C4orf37	2.185E-25	0.44	34.96	80.09	1.00	1.00	1.00
cg09727944	6	FAM184A; MIR548B	1.34E-23	0.30	24.37	80.84	1.00	1.00	1.00
cg09757709	11	SNORA32	1.252E-25	0.32	20.14	63.86	1.00	1.00	1.00
cg09766710	1	OR2G3	3.793E-27	0.46	30.56	66.02	1.00	1.00	1.00
cg09841898	14	MAP4K5	1.73E-25	0.43	33.75	78.28	1.00	1.00	1.00
cg09939375	4	NSUN7	1.708E-24	0.33	25.47	76.05	1.00	1.00	1.00
cg10012711	6	SCAND3	1.803E-27	0.42	24.42	58.24	1.00	1.00	1.00
cg10039857	17	RHOT1	2.381E-25	0.45	37.24	82.58	1.00	1.00	1.00
cg10099105	7	INSIG1	6.204E-26	0.45	34.76	76.74	1.00	1.00	1.00
cg10112711	12	CAPS2	1.712E-25	0.40	30.14	74.65	1.00	1.00	1.00
cg10120616	7	SEMA3A	2.961E-27	0.49	33.06	67.97	1.00	1.00	1.00
cg10220637	2	ASNSD1	1.384E-25	0.47	39.44	83.41	1.00	1.00	1.00
cg10272779	16	RBBP6	4.076E-49	3.33	37.23	11.19	1.00	1.00	1.00
cg10291238	13	XPO4	2.421E-26	0.49	37.97	77.68	1.00	1.00	1.00
cg10312334	7	SGCE	1.147E-25	0.48	39.40	82.90	1.00	1.00	1.00

cg10312498	1	GLT25D2	2.53E-27	0.50	34.01	68.57	1.00	1.00	1.00
cg10357555	8	RALYL	9.132E-27	0.49	35.66	73.10	1.00	1.00	1.00
cg10376100	1	LYST;MIR1537	8.977E-27	0.47	33.63	71.03	1.00	1.00	1.00
cg10439742	16	HSBP1	2.473E-25	0.36	25.81	71.25	1.00	1.00	1.00
cg10451200	11	GRM5	1.014E-27	0.49	30.98	63.56	1.00	1.00	1.00
cg10483534	1	DISC2	2.451E-26	0.48	36.10	75.84	1.00	1.00	1.00
cg10501629	12	ATP2B1	3.256E-26	0.45	33.29	73.71	1.00	1.00	1.00
cg10620767	10	ZMYND11	5.976E-26	0.48	38.74	80.62	1.00	1.00	1.00
cg10633981	11	C11orf58	4.517E-28	0.24	9.69	40.57	1.00	1.00	1.00
cg10666761	15	PRTG	1.211E-25	0.40	28.59	72.22	1.00	1.00	1.00
cg10673484	3	PLCL2	1.669E-26	0.44	30.68	69.52	1.00	1.00	1.00
cg10685982	7	AGBL3	4.353E-26	0.49	39.47	80.59	1.00	1.00	1.00
cg10788939	5	EFNA5	2.213E-26	0.48	37.01	76.51	1.00	1.00	1.00
cg10813585	1	RSBN1	3.553E-26	0.44	31.41	72.04	1.00	1.00	1.00
cg10818896	6	C6orf10	1.1E-26	0.45	30.38	68.25	1.00	1.00	1.00
cg10874881	7	POU6F2	1.229E-25	0.39	28.16	71.83	1.00	1.00	1.00
cg10892127	3	ZNF445	1.104E-46	2.69	35.22	13.09	1.00	1.00	1.00
cg10909192	14	PRPF39;SNORD127	6.514E-26	0.46	35.68	77.78	1.00	1.00	1.00
cg10916646	6	OR12D2	2.604E-25	0.38	28.01	73.59	1.00	1.00	1.00
cg10931901	14	SNORD114-11; SNORD114-10	1.725E-24	0.37	29.68	80.29	1.00	1.00	1.00
cg10943931	15	USP8	9.549E-24	0.35	29.58	85.04	1.00	1.00	1.00
cg10963375	1	PM20D1	1.499E-26	0.49	36.95	75.54	1.00	1.00	1.00
cg10982500	5	FBXL17	3.45E-26	0.46	34.03	74.58	1.00	1.00	1.00
cg10994681	11	ELP4	5.133E-25	0.35	25.89	73.23	1.00	1.00	1.00
cg11050480	10	AS3MT	1.793E-25	0.44	35.74	80.37	1.00	1.00	1.00
cg11077161	14	SIPA1L1	1.559E-25	0.41	30.99	75.26	1.00	1.00	1.00
cg11138318	1	AIDA;C1orf58	1.54E-28	0.48	20.34	42.08	1.00	1.00	1.00
cg11210449	7	CNOT4	8.316E-26	0.48	38.73	81.43	1.00	1.00	1.00
cg11368438	3	SR140	5.29E-26	0.50	41.00	82.59	1.00	1.00	1.00
cg11389618	2	GTDC1	4.826E-26	0.44	32.42	73.79	1.00	1.00	1.00
cg11489638	3	LOC285205	7.91E-27	0.47	32.59	69.70	1.00	1.00	1.00
cg11498516	4	CCDC109B	4.951E-25	0.42	34.73	81.98	1.00	1.00	1.00
cg11531783	7	MLL5	2.466E-27	0.36	19.38	53.88	1.00	1.00	1.00
cg11554605	7	ASB4	1.876E-25	0.44	34.51	79.25	1.00	1.00	1.00
cg11598658	12	NAV3	7.244E-26	0.46	36.08	78.44	1.00	1.00	1.00
cg11620689	3	ALCAM	9.33E-25	0.36	26.99	75.92	1.00	1.00	1.00
cg11632652	3	NAALADL2	6.095E-26	0.47	37.38	79.31	1.00	1.00	1.00
cg11643733	12	PPFIA2	1.54E-26	0.47	34.67	73.32	1.00	1.00	1.00
cg11653675	2	RHOQ;PIGF	1.371E-25	0.44	34.49	78.43	1.00	1.00	1.00
cg11739512	1	NBPF3	5.876E-26	0.47	37.58	79.43	1.00	1.00	1.00
cg11739612	6	MRS2	1.983E-25	0.46	38.37	83.25	1.00	1.00	1.00
cg11832339	11	BBOX1	4.372E-27	0.49	34.96	70.74	1.00	1.00	1.00
cg11879776	20	CST8	2.068E-26	0.50	39.28	78.62	1.00	1.00	1.00
cg11976671	6	GRIK2	2.012E-26	0.49	37.82	77.09	1.00	1.00	1.00
cg11979846	1	ZNF124	2.773E-50	2.03	54.78	27.00	1.00	1.00	1.00
cg11990610	2	SPAG16	5.967E-26	0.49	40.39	82.28	1.00	1.00	1.00
cg12089229	17	NSF	3.86E-26	0.44	31.88	72.71	1.00	1.00	1.00

cg12105450	2	CASP10	4.765E-44	2.28	30.99	13.60	1.00	1.00	1.00
cg12120845	8	TOX	1.066E-25	0.46	36.93	80.24	1.00	1.00	1.00
cg12133103	8	CRISPLD1	2.971E-27	0.48	32.28	67.20	1.00	1.00	1.00
cg12155028	5	PRR16	1.528E-26	0.42	27.59	66.22	1.00	1.00	1.00
cg12185938	2	VWA3B	5.647E-26	0.45	34.34	76.09	1.00	1.00	1.00
cg12240515	8	EFR3A	1.739E-25	0.47	39.44	83.99	1.00	1.00	1.00
cg12250761	11	OR52K2	1.244E-25	0.48	40.96	84.66	1.00	1.00	1.00
cg12406363	1	NVL	1.155E-25	0.41	29.80	73.32	1.00	1.00	1.00
cg12485044	13	HMGB1	1.16E-25	0.40	28.68	72.20	1.00	1.00	1.00
cg12499092	12	NFYB	2.089E-25	0.45	37.37	82.38	1.00	1.00	1.00
cg12510717	13	GPC5	7.89E-26	0.44	33.52	76.08	1.00	1.00	1.00
cg12553181	4	UNC5C	2.375E-25	0.44	35.99	81.33	1.00	1.00	1.00
cg12556823	6	CDKAL1	1.433E-25	0.48	40.57	84.62	1.00	1.00	1.00
cg12561169	4	FRAS1	1.003E-26	0.49	35.82	73.48	1.00	1.00	1.00
cg12590005	6	MAPK14	1.753E-51	3.39	41.83	12.34	1.00	1.00	1.00
cg12602653	18	MEP1B	8.549E-27	0.50	37.06	74.35	1.00	1.00	1.00
cg12635662	11	PLAC1L	2.512E-25	0.40	30.16	75.64	1.00	1.00	1.00
cg12671750	18	MYO5B	2.997E-25	0.39	29.88	75.82	1.00	1.00	1.00
cg12686777	6	C6orf182	3.244E-26	0.44	31.52	71.93	1.00	1.00	1.00
cg12688788	11	FANCF	3.107E-26	0.47	35.56	75.87	1.00	1.00	1.00
cg12700074	6	AKAP7	2.844E-43	2.59	25.84	9.96	1.00	1.00	1.00
cg12718307	1	LYST	2.458E-25	0.46	38.03	83.46	1.00	1.00	1.00
cg12723191	1	PDC;MIR548F1	3.295E-26	0.45	32.50	72.95	1.00	1.00	1.00
cg12725520	14	SNORD114-16	1.934E-23	0.26	20.24	77.81	1.00	1.00	1.00
cg12732390	3	ROBO1	1.849E-26	0.47	35.00	74.08	1.00	1.00	1.00
cg12748491	4	PALLD	2.133E-25	0.43	33.55	78.61	1.00	1.00	1.00
cg12762303	6	CCNC;CCNC	1.013E-25	0.47	37.93	81.12	1.00	1.00	1.00
cg12768447	10	CTNNA3	3.043E-28	0.47	26.67	56.73	1.00	1.00	1.00
cg12827708	18	DTNA	7.761E-27	0.49	35.55	72.62	1.00	1.00	1.00
cg12874219	3	TM4SF4	4.209E-26	0.45	33.99	75.03	1.00	1.00	1.00
cg12879425	4	STAP1	5.538E-57	2.80	56.59	20.21	1.00	1.00	1.00
cg12921799	21	SFRS15	5.372E-26	0.44	32.86	74.49	1.00	1.00	1.00
cg12923233	15	UBE3A	5.268E-26	0.44	32.64	74.22	1.00	1.00	1.00
cg12955305	7	TMEM196	1.099E-25	0.43	33.02	76.42	1.00	1.00	1.00
cg12959121	14	KIAA0586;TIMM9	4.996E-27	0.48	32.71	68.78	1.00	1.00	1.00
cg12989745	6	TRIM27	6.531E-26	0.49	41.20	83.30	1.00	1.00	1.00
cg13077930	4	ALB	1.16E-25	0.40	28.49	72.02	1.00	1.00	1.00
cg13101088	2	PELI1	2.578E-25	0.43	33.70	79.25	1.00	1.00	1.00
cg13139697	14	RTN1	1.251E-26	0.38	23.34	61.51	1.00	1.00	1.00
cg13189979	12	PTPRQ	5.467E-24	0.31	24.56	78.41	1.00	1.00	1.00
cg13283932	6	EYA4	2.315E-25	0.40	29.91	75.18	1.00	1.00	1.00
cg13313226	11	C11orf61	5.99E-26	0.49	40.93	82.83	1.00	1.00	1.00
cg13370754	5	SSBP2	1.508E-25	0.43	33.78	77.97	1.00	1.00	1.00
cg13380669	1	C1orf114	1.63E-25	0.48	41.69	86.07	1.00	1.00	1.00
cg13434757	6	SLC22A1	6.374E-25	0.36	27.12	75.03	1.00	1.00	1.00
cg13442201	1	DUSP10	5.286E-26	0.46	35.62	77.21	1.00	1.00	1.00
cg13503148	3	TMEM212	1.647E-26	0.48	36.20	75.01	1.00	1.00	1.00
cg13512333	11	PDE3B	6.055E-26	0.45	34.86	76.78	1.00	1.00	1.00

cg13523654	18	CHST9	6.416E-27	0.50	36.22	72.86	1.00	1.00	1.00
cg13539591	17	ZNF286B	4.301E-26	0.47	35.98	77.07	1.00	1.00	1.00
cg13548607	12	EPYC	2.796E-25	0.37	26.80	72.56	1.00	1.00	1.00
cg13573178	1	ZNF326	8.353E-26	0.46	35.74	78.45	1.00	1.00	1.00
cg13623384	6	WTAP	2.912E-25	0.44	35.86	81.73	1.00	1.00	1.00
cg13636907	3	MITF	2.188E-27	0.50	33.90	68.15	1.00	1.00	1.00
cg13680118	14	NRXN3	1.825E-24	0.38	31.29	82.06	1.00	1.00	1.00
cg13734338	2	WDR35	1.353E-25	0.37	25.96	69.88	1.00	1.00	1.00
cg13748640	9	ERP44	1.185E-25	0.50	43.08	86.66	1.00	1.00	1.00
cg13759778	17	NF1;OMG	5.75E-26	0.50	41.28	83.07	1.00	1.00	1.00
cg13828600	7	KLHL7	2.116E-50	2.82	43.28	15.33	1.00	1.00	1.00
cg13866618	6	C6orf218	1.992E-47	3.51	32.68	9.32	1.00	1.00	1.00
cg13887068	6	MED23	8.201E-26	0.46	36.43	79.09	1.00	1.00	1.00
cg13906954	3	C3orf63	3.112E-25	0.34	23.38	69.42	1.00	1.00	1.00
cg14018471	9	C9orf156	8.347E-59	2.39	66.03	27.65	1.00	1.00	1.00
cg14064774	4	FRYL	6.333E-26	0.41	29.49	71.52	1.00	1.00	1.00
cg14102434	14	C14orf105	5.583E-26	0.49	39.85	81.57	1.00	1.00	1.00
cg14125733	14	SNORD114-5	6.011E-25	0.36	27.17	74.93	1.00	1.00	1.00
cg14146966	2	XPO1	2.164E-27	0.49	32.82	67.04	1.00	1.00	1.00
cg14150751	6	SENP6	3.855E-25	0.42	33.41	80.01	1.00	1.00	1.00
cg14157107	9	BNC2	2.231E-25	0.44	35.47	80.65	1.00	1.00	1.00
cg14258031	9	PTPRD	1.029E-25	0.43	32.01	75.24	1.00	1.00	1.00
cg14292424	9	SUGT1P1;NOL6	5.692E-26	0.47	37.05	78.82	1.00	1.00	1.00
cg14322040	4	AMBN	7.629E-26	0.40	28.22	70.71	1.00	1.00	1.00
cg14371343	7	CADPS2	5.319E-25	0.39	30.80	78.24	1.00	1.00	1.00
cg14376033	15	RAB8B	7.924E-26	0.46	35.61	78.19	1.00	1.00	1.00
cg14421309	2	ZEB2	8.795E-47	2.13	42.08	19.79	1.00	1.00	1.00
cg14466576	3	TOPBP1	9.593E-27	0.48	34.50	72.05	1.00	1.00	1.00
cg14479617	3	GSK3B	2.623E-27	0.50	34.13	68.78	1.00	1.00	1.00
cg14484681	9	SETX	5.3E-26	0.45	33.86	75.45	1.00	1.00	1.00
cg14500932	9	DENND1A	2.706E-26	0.45	32.29	72.27	1.00	1.00	1.00
cg14523881	1	VASH2	1.364E-25	0.47	39.50	83.43	1.00	1.00	1.00
cg14542367	9	TYRP1	5.988E-27	0.45	29.76	66.24	1.00	1.00	1.00
cg14566819	9	HEMGN	8.831E-27	0.44	29.57	66.93	1.00	1.00	1.00
cg14567260	5	NDUFAF2	9.451E-27	0.49	35.67	73.19	1.00	1.00	1.00
cg14617014	1	CLCA3P	5.123E-25	0.38	29.20	76.53	1.00	1.00	1.00
cg14620593	17	MYH8	1.8E-25	0.41	31.25	75.88	1.00	1.00	1.00
cg14630237	7	CYP51A1	3.964E-26	0.44	32.43	73.32	1.00	1.00	1.00
cg14638735	5	SNX2	7.331E-26	0.44	33.51	75.90	1.00	1.00	1.00
cg14694205	3	GMPS	3.829E-26	0.47	36.83	77.64	1.00	1.00	1.00
cg14706107	11	SFRS2B	7.486E-43	2.02	29.73	14.69	1.00	1.00	1.00
cg14712202	1	DISP1	8.129E-27	0.47	32.75	69.93	1.00	1.00	1.00
cg14821923	5	C5orf36	2.867E-27	0.49	32.98	67.81	1.00	1.00	1.00
cg14841453	12	OR6C68	2.458E-26	0.50	39.51	79.26	1.00	1.00	1.00
cg14849228	5	KIAA0947	6.198E-26	0.46	35.85	77.82	1.00	1.00	1.00
cg14878128	7	ABCB5	7.738E-27	0.43	27.84	64.90	1.00	1.00	1.00
cg14931071	6	PHACTR1	3.171E-25	0.40	31.19	77.28	1.00	1.00	1.00
cg14944166	7	CHRM2	2.197E-26	0.47	34.80	74.28	1.00	1.00	1.00

cg14975015	7	ATP6V0A4	2.319E-26	0.44	30.52	70.13	1.00	1.00	1.00
cg14985421	6	RNF182	2.681E-24	0.27	19.66	71.49	1.00	1.00	1.00
cg14995160	10	SLC18A2	5.392E-27	0.36	20.11	56.35	1.00	1.00	1.00
cg15018391	6	GPR63	4.571E-26	0.44	32.28	73.51	1.00	1.00	1.00
cg15026099	1	TCTEX1D1	1.328E-23	0.29	23.47	79.91	1.00	1.00	1.00
cg15073906	1	RALGPS2	2.548E-25	0.42	33.01	78.53	1.00	1.00	1.00
cg15089217	3	CCDC14	1.8E-27	0.46	29.16	62.98	1.00	1.00	1.00
cg15092420	4	LCORL	5.313E-26	0.45	34.49	76.09	1.00	1.00	1.00
cg15113916	3	C3orf57	9.826E-26	0.40	28.51	71.62	1.00	1.00	1.00
cg15149117	11	OR52H1	1.416E-29	0.48	22.20	46.26	1.00	1.00	1.00
cg15164860	2	PUM2	2.637E-25	0.46	38.88	84.49	1.00	1.00	1.00
cg15239914	2	SCN2A	2.284E-25	0.42	32.55	77.79	1.00	1.00	1.00
cg15269541	15	ADAL	8.302E-26	0.45	34.63	77.33	1.00	1.00	1.00
cg15353821	4	CPE	2.359E-26	0.49	38.29	77.94	1.00	1.00	1.00
cg15420906	14	FAM71D	3.048E-26	0.48	36.90	77.16	1.00	1.00	1.00
cg15459822	2	PARD3B	3.494E-27	0.32	16.94	52.21	1.00	1.00	1.00
cg15462736	1	RASAL2	7.936E-26	0.36	24.11	66.69	1.00	1.00	1.00
cg15510118	2	SP3	4.161E-26	0.46	35.52	76.53	1.00	1.00	1.00
cg15518400	2	CALM2	8.049E-52	2.39	51.52	21.56	1.00	1.00	1.00
cg15519065	1	EIF4G3	6.463E-47	2.47	37.86	15.35	1.00	1.00	1.00
cg15545021	2	LCLAT1;LCLAT1	1.451E-25	0.39	28.31	72.40	1.00	1.00	1.00
cg15553612	1	TBX15	2.755E-27	0.47	30.48	65.23	1.00	1.00	1.00
cg15554421	3	C3orf26;FILIP1L	5.455E-48	2.64	39.09	14.81	1.00	1.00	1.00
cg15564000	1	TRIM33	2.005E-26	0.42	27.87	67.14	1.00	1.00	1.00
cg15571380	6	ME1	2.113E-25	0.46	38.22	83.26	1.00	1.00	1.00
cg15573998	2	NRXN1	4.218E-26	0.50	40.25	81.29	1.00	1.00	1.00
cg15611556	4	SYT14L;TMPRSS11F	1.478E-29	0.50	23.99	48.12	1.00	1.00	1.00
cg15627442	8	ADAM5P	1.691E-25	0.41	30.42	74.89	1.00	1.00	1.00
cg15679981	2	SRBD1	8.023E-28	0.47	28.24	60.32	1.00	1.00	1.00
cg15681326	3	NCEH1	6.796E-26	0.49	40.43	82.63	1.00	1.00	1.00
cg15684210	5	MBLAC2	1.532E-25	0.35	23.34	67.57	1.00	1.00	1.00
cg15700487	1	GOLPH3L	7.901E-45	2.44	31.98	13.13	1.00	1.00	1.00
cg15700870	5	PPP2R2B	1.161E-25	0.47	39.03	82.56	1.00	1.00	1.00
cg15749858	7	MACC1	9.883E-47	3.34	31.70	9.50	1.00	1.00	1.00
cg15781504	11	OR51S1	2.643E-26	0.43	30.46	70.38	1.00	1.00	1.00
cg15819780	17	TOM1L1;COX11	1.221E-26	0.47	34.25	72.36	1.00	1.00	1.00
cg15826891	11	LOC399959	5.795E-25	0.41	33.76	81.42	1.00	1.00	1.00
cg15851696	2	PDE1A	5.774E-25	0.38	29.48	77.13	1.00	1.00	1.00
cg15878685	4	UGT2A3	1.01E-26	0.48	34.44	72.11	1.00	1.00	1.00
cg15911114	5	FST	2.119E-28	0.37	17.21	46.53	1.00	1.00	1.00
cg15922045	3	COL6A6	2.546E-26	0.43	29.63	69.47	1.00	1.00	1.00
cg15924285	11	SNORA8;SNORA1;<br>SNORA32;SNORD6	4.32E-26	0.44	31.95	73.05	1.00	1.00	1.00
cg15928016	10	MARCH5	3.79E-26	0.49	38.81	79.59	1.00	1.00	1.00
cg15943396	5	TTC1	4.958E-17	2.58	19.75	7.66	1.00	1.00	1.00
cg15985533	1	TYW3	9.034E-29	0.38	16.68	44.29	1.00	1.00	1.00
cg15990658	1	ASB17	2.174E-25	0.43	33.92	79.03	1.00	1.00	1.00
cg15993521	12	SLC35E3	4.271E-25	0.45	37.87	84.73	1.00	1.00	1.00

cg16019620	6	MEP1A	1.797E-25	0.48	40.50	85.13	1.00	1.00	1.00
cg16036046	18	RIT2	1.766E-26	0.47	35.14	74.11	1.00	1.00	1.00
cg16109012	1	ZBTB41	8.158E-24	0.24	17.45	72.46	1.00	1.00	1.00
cg16137285	6	CDC40	6.642E-26	0.47	37.30	79.45	1.00	1.00	1.00
cg16143105	5	HOMER1	2.67E-26	0.46	33.79	73.74	1.00	1.00	1.00
cg16189217	13	PCCA	3.237E-26	0.48	37.77	78.17	1.00	1.00	1.00
cg16225218	5	FBXW11	1.029E-25	0.36	23.80	67.03	1.00	1.00	1.00
cg16230553	5	MAST4	2.437E-25	0.45	37.23	82.64	1.00	1.00	1.00
cg16242080	2	FAM171B	8.249E-26	0.40	28.24	70.92	1.00	1.00	1.00
cg16274221	1	OR2T1	2.799E-27	0.49	34.09	68.88	1.00	1.00	1.00
cg16395911	14	EXOC5	3.488E-24	0.30	22.31	74.88	1.00	1.00	1.00
cg16430955	14	FAM177A1	1.657E-25	0.40	29.33	73.76	1.00	1.00	1.00
cg16439360	3	ZNF385D	2.597E-26	0.47	34.67	74.55	1.00	1.00	1.00
cg16456371	6	USP49	3.086E-25	0.44	36.22	82.23	1.00	1.00	1.00
cg16463745	5	MAT2B	9.638E-43	2.52	24.56	9.74	1.00	1.00	1.00
cg16465559	8	LRP12	1.747E-26	0.49	37.73	76.67	1.00	1.00	1.00
cg16491233	18	PTPN2	1.89E-25	0.40	29.50	74.26	1.00	1.00	1.00
cg16503628	3	FGF12	2.681E-27	0.45	28.74	63.43	1.00	1.00	1.00
cg16513933	8	ANKRD46	4.572E-25	0.41	32.43	79.47	1.00	1.00	1.00
cg16525066	16	CDH11	9.493E-47	3.55	30.94	8.71	1.00	1.00	1.00
cg16529799	5	MCTP1	7.463E-26	0.38	26.29	68.73	1.00	1.00	1.00
cg16540183	2	RAD51AP2	5.715E-25	0.37	27.57	75.20	1.00	1.00	1.00
cg16551220	14	GPR137C	1.269E-24	0.39	31.55	81.32	1.00	1.00	1.00
cg16659470	8	RBM12B	3.552E-29	0.28	9.97	35.77	1.00	1.00	1.00
cg16720242	1	IPP	5.52E-26	0.48	38.62	80.31	1.00	1.00	1.00
cg16740092	4	ANTXR2	2.876E-25	0.40	31.00	76.83	1.00	1.00	1.00
cg16798617	7	RBM33	6.923E-25	0.40	31.46	79.60	1.00	1.00	1.00
cg16873130	7	MIR490;CHRM2	8.583E-27	0.25	12.16	49.46	1.00	1.00	1.00
cg16970798	3	CLDN16	6.361E-27	0.45	29.69	66.31	1.00	1.00	1.00
cg17004212	13	DLEU1	1.281E-20	0.32	5.29	16.59	1.00	1.00	1.00
cg17056062	13	KATNAL1	1.122E-25	0.50	42.69	86.13	1.00	1.00	1.00
cg17145862	1	LPGAT1	6.661E-26	0.43	31.91	74.06	1.00	1.00	1.00
cg17146731	4	PTPN13	2.022E-25	0.37	26.38	71.31	1.00	1.00	1.00
cg17158101	7	CBLL1	4.329E-26	0.43	30.91	72.01	1.00	1.00	1.00
cg17174566	2	ACVR2A	4.88E-27	0.49	34.26	70.28	1.00	1.00	1.00
cg17282466	4	LOC100130017	1.217E-25	0.45	36.20	79.85	1.00	1.00	1.00
cg17374958	3	SCHIP1	1.989E-26	0.48	36.16	75.41	1.00	1.00	1.00
cg17383024	4	C4orf36	1.682E-22	0.34	6.68	19.45	1.00	1.00	1.00
cg17413252	6	ENPP1	5.966E-28	0.48	29.52	60.98	1.00	1.00	1.00
cg17469276	1	ATP1A1	6.616E-27	0.37	21.50	58.20	1.00	1.00	1.00
cg17480438	6	KIAA1244	3.727E-26	0.40	26.64	67.38	1.00	1.00	1.00
cg17480669	5	RPL37	1.911E-22	0.36	7.25	20.31	1.00	1.00	1.00
cg17516635	8	SNX16	6.033E-26	0.35	22.28	64.19	1.00	1.00	1.00
cg17582444	3	MFN1	3.85E-25	0.40	30.74	77.33	1.00	1.00	1.00
cg17643729	3	LOC344595	7.501E-27	0.49	35.55	72.54	1.00	1.00	1.00
cg17746031	17	KCNJ16	5.263E-25	0.35	25.55	72.96	1.00	1.00	1.00
cg17758905	1	SKINTL	2.035E-26	0.41	27.14	66.44	1.00	1.00	1.00
cg17771150	13	LCP1	3.307E-14	2.48	17.75	7.15	1.00	1.00	1.00

cg17807683	17	OR1D2	2.211E-25	0.42	33.18	78.33	1.00	1.00	1.00
cg17829914	5	FCHO2	4.501E-28	0.44	24.35	55.22	1.00	1.00	1.00
cg18011148	20	DEFB132	3.567E-24	0.37	30.54	83.17	1.00	1.00	1.00
cg18016759	6	POLH	2.197E-27	0.50	34.01	68.26	1.00	1.00	1.00
cg18048983	17	C17orf104	2.913E-26	0.46	34.25	74.41	1.00	1.00	1.00
cg18082082	6	DEFB113	1.73E-26	0.42	28.71	67.63	1.00	1.00	1.00
cg18090487	13	TNFSF11	1.32E-27	0.50	32.96	66.11	1.00	1.00	1.00
cg18182039	12	PLEKHG7	9.102E-27	0.43	28.54	65.97	1.00	1.00	1.00
cg18220666	4	EREG	2.336E-25	0.44	36.26	81.56	1.00	1.00	1.00
cg18259092	11	SESN3	1.088E-26	0.48	34.31	72.16	1.00	1.00	1.00
cg18295261	14	FBXO34	4.063E-26	0.40	27.38	68.33	1.00	1.00	1.00
cg18368637	6	LTV1	1.27E-27	0.49	31.75	64.81	1.00	1.00	1.00
cg18402991	4	ART3;CXCL11	1.426E-28	0.45	23.17	51.69	1.00	1.00	1.00
cg18403754	14	RAD51L1	2.63E-25	0.42	33.00	78.60	1.00	1.00	1.00
cg18458739	17	BCAS3	6.655E-25	0.39	30.17	78.20	1.00	1.00	1.00
cg18459342	8	TPD52	3.67E-28	0.37	9.89	26.58	1.00	1.00	1.00
cg18590130	11	LOC399959; MIRLET7A2	8.159E-27	0.43	27.82	65.00	1.00	1.00	1.00
cg18637238	12	KLRK1	9.424E-25	0.33	24.05	73.01	1.00	1.00	1.00
cg18705155	6	KCNK5	6.485E-44	2.45	28.97	11.84	1.00	1.00	1.00
cg18793322	5	IQGAP2	7.685E-25	0.31	21.29	69.70	1.00	1.00	1.00
cg18816605	1	PTPN14	1.485E-26	0.46	32.45	71.02	1.00	1.00	1.00
cg18841796	5	RAPGEF6	1.348E-27	0.43	25.37	58.56	1.00	1.00	1.00
cg18862260	2	MSTN	7.26E-26	0.48	38.74	81.10	1.00	1.00	1.00
cg18890249	10	LOC728407	3.347E-26	0.50	40.22	80.70	1.00	1.00	1.00
cg18907770	13	POMP	3.452E-25	0.43	34.34	80.64	1.00	1.00	1.00
cg18979762	1	EGLN1	4.546E-24	0.36	30.61	83.94	1.00	1.00	1.00
cg18982923	10	OAT	1.382E-26	0.48	36.05	74.45	1.00	1.00	1.00
cg18983986	2	C2orf86	5.371E-27	0.49	34.19	70.43	1.00	1.00	1.00
cg19000611	14	CATSPERB	3.543E-28	0.49	29.04	59.41	1.00	1.00	1.00
cg19018435	1	RGL1	8.798E-27	0.50	36.65	74.01	1.00	1.00	1.00
cg19048331	20	BTBD3	1.923E-27	0.34	17.48	51.44	1.00	1.00	1.00
cg19108435	5	LVRN	9.592E-27	0.47	33.21	70.76	1.00	1.00	1.00
cg19159222	15	C15orf33;DTWD1	4.131E-46	2.10	40.42	19.28	1.00	1.00	1.00
cg19168192	6	GPX5	2.009E-45	2.27	35.66	15.73	1.00	1.00	1.00
cg19224886	12	ZFC3H1	5.614E-25	0.38	29.09	76.67	1.00	1.00	1.00
cg19273756	17	SKA2;MIR301A	2.615E-25	0.45	37.24	82.83	1.00	1.00	1.00
cg19307180	13	DGKH	1.275E-25	0.45	35.77	79.53	1.00	1.00	1.00
cg19342530	4	GUCY1B3	1.946E-26	0.49	37.52	76.72	1.00	1.00	1.00
cg19374779	3	FAIM	3.353E-26	0.48	37.01	77.50	1.00	1.00	1.00
cg19391535	7	CUL1	1.456E-49	2.04	52.53	25.81	1.00	1.00	1.00
cg19427399	20	WFDC8	1.8E-26	0.48	35.37	74.38	1.00	1.00	1.00
cg19449969	12	PPHLN1	8.267E-26	0.42	30.39	73.07	1.00	1.00	1.00
cg19457823	5	NR3C1	3.256E-26	0.48	36.84	77.26	1.00	1.00	1.00
cg19521384	6	MIR548H3	3.399E-26	0.49	39.69	80.21	1.00	1.00	1.00
cg19585214	12	SLC38A1	3.124E-26	0.49	37.98	78.30	1.00	1.00	1.00
cg19647111	6	TNXB	9.566E-28	0.49	31.66	64.12	1.00	1.00	1.00
cg19672694	5	PTGER4	4.726E-47	2.35	39.59	16.84	1.00	1.00	1.00

cg19714332	13	NDFIP2	4.601E-27	0.49	34.36	70.25	1.00	1.00	1.00
cg19747990	5	EDIL3	1.248E-24	0.31	22.70	72.42	1.00	1.00	1.00
cg19756042	10	HNRNPH3	1.848E-26	0.46	33.75	72.83	1.00	1.00	1.00
cg19775071	5	DEPDC1B	1.746E-26	0.47	34.66	73.60	1.00	1.00	1.00
cg19781863	12	MON2	2.41E-27	0.44	26.54	60.99	1.00	1.00	1.00
cg19796955	12	TBK1	1.081E-24	0.43	37.59	86.93	1.00	1.00	1.00
cg19800407	7	ORC5L	8.316E-26	0.48	39.99	82.69	1.00	1.00	1.00
cg19847588	8	GTF2E2	8.674E-26	0.46	35.98	78.78	1.00	1.00	1.00
cg19929852	4	CPEB2	9.307E-25	0.34	25.59	74.51	1.00	1.00	1.00
cg19945957	20	PIGU	3.056E-13	0.47	8.87	18.95	1.00	1.00	1.00
cg19950767	13	TPTE2	1.817E-26	0.44	30.43	69.47	1.00	1.00	1.00
cg19955105	2	CLIP4	5.798E-25	0.40	31.52	79.18	1.00	1.00	1.00
cg19993440	10	PTPN20B;PTPN20A	2.838E-26	0.45	32.88	72.97	1.00	1.00	1.00
cg19999035	4	SHROOM3	2.52E-26	0.45	33.18	72.99	1.00	1.00	1.00
cg20011278	4	ALPK1	4.195E-26	0.42	30.01	71.04	1.00	1.00	1.00
cg20019217	15	RAB27A	5.071E-47	3.25	32.78	10.08	1.00	1.00	1.00
cg20022118	7	ICA1	2.688E-27	0.47	30.50	65.19	1.00	1.00	1.00
cg20043699	12	KERA	1.742E-26	0.48	36.47	75.41	1.00	1.00	1.00
cg20092728	11	SLC5A12	3.295E-26	0.44	31.39	71.84	1.00	1.00	1.00
cg20101015	11	CUGBP1	1.954E-26	0.47	34.55	73.76	1.00	1.00	1.00
cg20195272	11	MTMR2	4.618E-26	0.46	35.53	76.79	1.00	1.00	1.00
cg20253302	4	LRP2BP	2.026E-24	0.39	32.77	83.82	1.00	1.00	1.00
cg20281583	4	NEIL3	3.205E-26	0.43	30.87	71.26	1.00	1.00	1.00
cg20333067	17	ZNF207;MIR632	7.055E-18	0.49	13.71	27.94	1.00	1.00	1.00
cg20346229	2	MRPS9	3.092E-25	0.38	27.64	73.66	1.00	1.00	1.00
cg20353780	12	TAOK3	5.22E-26	0.33	20.63	62.19	1.00	1.00	1.00
cg20393620	6	SGK1	1.147E-49	2.09	51.44	24.57	1.00	1.00	1.00
cg20469666	20	TOP1	5.807E-26	0.49	39.81	81.63	1.00	1.00	1.00
cg20482341	10	LOC728190	1.198E-25	0.47	39.25	82.86	1.00	1.00	1.00
cg20498482	8	VPS13B	3.226E-26	0.46	35.08	75.47	1.00	1.00	1.00
cg20511453	1	MIA3	1.317E-26	0.50	37.72	76.00	1.00	1.00	1.00
cg20546098	2	PMS1	4.193E-27	0.50	35.24	70.92	1.00	1.00	1.00
cg20567355	8	PCMTD1	2.148E-26	0.42	28.47	67.90	1.00	1.00	1.00
cg20586900	3	KCNAB1	2.184E-24	0.33	25.05	76.31	1.00	1.00	1.00
cg20597409	14	SLC25A21; LOC100129794	7.295E-27	0.48	33.52	70.45	1.00	1.00	1.00
cg20667822	11	PTPRJ	1.716E-25	0.42	32.58	77.09	1.00	1.00	1.00
cg20701810	5	FAM172A	5.082E-25	0.44	37.18	84.49	1.00	1.00	1.00
cg20725907	3	C3orf58	5.221E-28	0.49	29.39	60.57	1.00	1.00	1.00
cg20745925	5	GPBP1	6.975E-26	0.46	35.77	78.04	1.00	1.00	1.00
cg20771595	7	SEMA3C	5.337E-25	0.39	30.84	78.29	1.00	1.00	1.00
cg20800199	13	TDRD3	5.736E-26	0.46	36.23	78.02	1.00	1.00	1.00
cg20824876	2	MORN2	1.14E-25	0.49	41.43	84.91	1.00	1.00	1.00
cg20906802	6	OPN5	5.245E-26	0.47	36.71	78.28	1.00	1.00	1.00
cg20934215	3	PDZRN3	7.72E-26	0.48	39.53	82.04	1.00	1.00	1.00
cg20988616	6	ENPP3	9.98E-26	0.50	42.30	85.45	1.00	1.00	1.00
cg20997268	7	CREB5	4.178E-27	0.43	27.35	63.02	1.00	1.00	1.00
cg21041956	12	NACA	6.152E-49	2.05	50.20	24.44	1.00	1.00	1.00

cg21053474	4	COX7B2	1.092E-28	0.48	25.45	53.44	1.00	1.00	1.00
cg21068736	6	RNF146	7.379E-26	0.47	37.78	80.19	1.00	1.00	1.00
cg21114429	14	C14orf138;SOS2	1.282E-25	0.45	35.58	79.36	1.00	1.00	1.00
cg21217221	14	FUT8	1.263E-25	0.33	21.17	64.91	1.00	1.00	1.00
cg21222681	9	ANXA1	2.707E-26	0.31	18.35	58.32	1.00	1.00	1.00
cg21350115	2	CALCRL	9.649E-47	2.53	36.72	14.49	1.00	1.00	1.00
cg21366602	1	EPS15	4.126E-26	0.47	36.01	77.00	1.00	1.00	1.00
cg21404851	2	ANKRD36	7.894E-25	0.38	30.21	78.70	1.00	1.00	1.00
cg21463140	4	NFXL1	2.375E-26	0.33	19.28	58.95	1.00	1.00	1.00
cg21536815	2	FBXO11	3.932E-26	0.47	36.95	77.82	1.00	1.00	1.00
cg21540399	3	PTPRG	2.306E-25	0.41	31.79	77.05	1.00	1.00	1.00
cg21566278	13	C13orf31	1.753E-25	0.46	37.66	82.23	1.00	1.00	1.00
cg21579239	15	TTBK2	1.972E-25	0.36	25.75	70.62	1.00	1.00	1.00
cg21645572	8	DEPDC6	4.349E-27	0.50	35.39	71.16	1.00	1.00	1.00
cg21750426	21	MIRLET7C;C21orf34; MIR99A	1.541E-24	0.35	27.33	77.63	1.00	1.00	1.00
cg21827384	6	KIAA1009	8.003E-27	0.49	36.20	73.34	1.00	1.00	1.00
cg21877473	15	CASC4	1.908E-28	0.46	24.55	53.66	1.00	1.00	1.00
cg21924447	2	THSD7B	6.065E-26	0.39	26.85	68.77	1.00	1.00	1.00
cg21928923	7	PPP1R9A	7.36E-25	0.37	28.31	76.61	1.00	1.00	1.00
cg21942815	1	CEPT1	8.061E-25	0.41	33.94	82.48	1.00	1.00	1.00
cg21949784	6	VNN3	3.33E-26	0.47	35.88	76.36	1.00	1.00	1.00
cg21956815	12	LRP6	1.343E-26	0.46	32.88	71.21	1.00	1.00	1.00
cg21960191	2	LOC100132215; EHBP1	6.004E-28	0.45	25.99	57.46	1.00	1.00	1.00
cg22007739	1	ASH1L	3.728E-25	0.37	27.36	73.87	1.00	1.00	1.00
cg22049038	7	CNTNAP2	3.68E-27	0.49	33.58	68.97	1.00	1.00	1.00
cg22103319	4	CNGA1	5.12E-23	0.25	20.54	81.09	1.00	1.00	1.00
cg22148297	13	GPR18;UBAC2	5.568E-11	2.12	17.77	8.39	1.00	1.00	1.00
cg22164531	1	RGS18	3.096E-25	0.41	31.34	77.37	1.00	1.00	1.00
cg22218543	17	ZNF652	2.683E-52	2.09	58.65	28.05	1.00	1.00	1.00
cg22223969	13	DZIP1	1.348E-25	0.46	37.92	81.82	1.00	1.00	1.00
cg22295435	7	VSTM2A	1.883E-27	0.40	22.35	56.26	1.00	1.00	1.00
cg22341533	12	DCP1B	1.003E-24	0.42	35.24	84.37	1.00	1.00	1.00
cg22356541	13	FGF9	1.675E-26	0.41	27.09	65.93	1.00	1.00	1.00
cg22373676	13	UBL3	7.725E-26	0.40	28.40	70.91	1.00	1.00	1.00
cg22375856	3	TBC1D5	2.27E-26	0.46	33.20	72.76	1.00	1.00	1.00
cg22386358	6	C6orf168	1.126E-25	0.39	28.34	71.80	1.00	1.00	1.00
cg22540431	6	TRERF1	7.551E-26	0.49	41.50	83.96	1.00	1.00	1.00
cg22558389	4	GPRIN3	6.521E-26	0.41	29.43	71.54	1.00	1.00	1.00
cg22594071	3	PLSCR5	7.903E-26	0.45	34.87	77.44	1.00	1.00	1.00
cg22608848	4	HSD17B11	6.008E-26	0.47	37.51	79.42	1.00	1.00	1.00
cg22616933	7	KIAA1324L	2.528E-26	0.48	37.32	77.13	1.00	1.00	1.00
cg22688969	11	SC5DL	1.813E-26	0.44	30.20	69.23	1.00	1.00	1.00
cg22730988	8	FAM49B	2.561E-25	0.44	35.22	80.75	1.00	1.00	1.00
cg22799325	7	ZNF117	9.053E-27	0.49	36.37	73.79	1.00	1.00	1.00
cg22799510	3	PROK2	2.84E-27	0.43	26.13	60.95	1.00	1.00	1.00
cg22807241	7	MIR548F3;CNTNAP2	5.154E-27	0.49	34.19	70.33	1.00	1.00	1.00

cg22821358	1	MIR181B1	4.443E-26	0.44	32.93	74.09	1.00	1.00	1.00
cg22872634	1	SLC35A3	3.362E-25	0.39	30.18	76.42	1.00	1.00	1.00
cg22872857	8	XPO7	4.668E-26	0.47	36.59	77.88	1.00	1.00	1.00
cg22907692	14	SNORD114-29; SNORD114-28	2.871E-28	0.48	28.00	57.94	1.00	1.00	1.00
cg23002008	8	MTUS1	1.253E-25	0.47	38.18	81.90	1.00	1.00	1.00
cg23049758	17	SPAG9	8.141E-27	0.47	32.93	70.11	1.00	1.00	1.00
cg23051372	8	ESRP1	8.756E-27	0.29	14.98	52.32	1.00	1.00	1.00
cg23052386	13	ANKRD10	3.606E-26	0.48	37.92	78.58	1.00	1.00	1.00
cg23086720	1	SAMD13	3.137E-48	3.15	36.11	11.45	1.00	1.00	1.00
cg23104549	7	AGK	7.237E-27	0.42	26.37	63.28	1.00	1.00	1.00
cg23113318	1	RNF11	3.645E-26	0.49	38.88	79.57	1.00	1.00	1.00
cg23136573	6	PAQR8	2.916E-27	0.48	32.21	67.08	1.00	1.00	1.00
cg23172892	6	RHAG	6.46E-27	0.43	27.39	64.04	1.00	1.00	1.00
cg23200873	3	SERPINI2	1.235E-26	0.49	36.63	74.77	1.00	1.00	1.00
cg23208678	7	HIBADH	1.827E-26	0.50	38.36	77.41	1.00	1.00	1.00
cg23283216	2	DUSP11	2.299E-25	0.42	33.14	78.40	1.00	1.00	1.00
cg23299469	12	IRAK4	2.006E-28	0.48	26.74	55.96	1.00	1.00	1.00
cg23325997	12	CLEC1B	2.457E-25	0.47	39.87	85.30	1.00	1.00	1.00
cg23331966	3	TMEM207	2.875E-25	0.40	30.84	76.68	1.00	1.00	1.00
cg23400451	17	MYH4	7.242E-27	0.50	36.46	73.38	1.00	1.00	1.00
cg23413494	8	CNOT7	5.207E-30	0.28	8.81	31.03	1.00	1.00	1.00
cg23434090	11	OR51L1	4.335E-26	0.42	29.79	70.90	1.00	1.00	1.00
cg23442187	14	G2E3	1.502E-24	0.37	29.72	79.95	1.00	1.00	1.00
cg23457506	2	LCLAT1	6.924E-26	0.45	34.12	76.37	1.00	1.00	1.00
cg23477257	7	PHTF2	1.063E-24	0.35	26.89	76.17	1.00	1.00	1.00
cg23481184	15	FAM189A1	5.011E-27	0.49	34.94	71.02	1.00	1.00	1.00
cg23540210	3	CCDC39	1.799E-24	0.34	26.53	77.25	1.00	1.00	1.00
cg23559636	8	RAD21	7.011E-26	0.49	40.86	83.14	1.00	1.00	1.00
cg23561752	13	ZNF828	1.422E-26	0.49	37.47	75.94	1.00	1.00	1.00
cg23598573	15	ZNF609	4.112E-25	0.44	36.89	83.65	1.00	1.00	1.00
cg23599860	14	ARID4A	3E-26	0.46	34.90	75.13	1.00	1.00	1.00
cg23610752	1	GORAB	2.401E-26	0.47	35.04	74.74	1.00	1.00	1.00
cg23698950	6	TRIM40	3.693E-24	0.23	16.14	68.87	1.00	1.00	1.00
cg23719367	8	LONRF1	6.547E-27	0.48	34.33	71.01	1.00	1.00	1.00
cg23725583	8	ENPP2	6.85E-27	0.48	33.96	70.75	1.00	1.00	1.00
cg23730277	4	BANK1	2.093E-26	0.49	38.12	77.49	1.00	1.00	1.00
cg23817637	10	CLRN3	1.839E-29	0.28	9.44	33.98	1.00	1.00	1.00
cg23963317	1	ESRRG	6.186E-43	2.10	29.08	13.87	1.00	1.00	1.00
cg23979520	11	MMP12	1.953E-26	0.46	33.52	72.73	1.00	1.00	1.00
cg23979876	4	SCOC	8.822E-24	0.27	20.30	75.54	1.00	1.00	1.00
cg24001985	15	RORA	6.618E-26	0.46	36.54	78.68	1.00	1.00	1.00
cg24030920	8	TMEM64	2.715E-25	0.43	33.78	79.47	1.00	1.00	1.00
cg24065957	5	ARL15	4.417E-26	0.49	38.89	80.04	1.00	1.00	1.00
cg24082983	21	KRTAP13-4	4.351E-27	0.36	20.03	55.79	1.00	1.00	1.00
cg24117017	1	FMO9P	2.101E-27	0.44	26.93	61.08	1.00	1.00	1.00
cg24310785	6	QKI	1.187E-25	0.44	34.94	78.53	1.00	1.00	1.00
cg24321113	3	BCHE	1.811E-25	0.31	19.63	64.28	1.00	1.00	1.00

cg24324572	11	HSD17B12	4.763E-26	0.48	38.11	79.44	1.00	1.00	1.00
cg24337173	11	STK33	2.804E-25	0.41	31.63	77.40	1.00	1.00	1.00
cg24340640	10	ADK	3.412E-26	0.46	34.77	75.30	1.00	1.00	1.00
cg24349668	18	SERPINB7	2.537E-27	0.48	31.56	66.13	1.00	1.00	1.00
cg24360745	7	PUS7	1.296E-25	0.46	37.09	80.89	1.00	1.00	1.00
cg24441954	6	ZSCAN23	1.857E-25	0.38	27.02	71.73	1.00	1.00	1.00
cg24509398	1	EYA3	1.423E-28	0.47	25.78	54.30	1.00	1.00	1.00
cg24613644	8	PSD3	1.221E-26	0.49	36.73	74.85	1.00	1.00	1.00
cg24655701	11	LOC399959;MIR100	7.341E-26	0.43	32.28	74.67	1.00	1.00	1.00
cg24657419	6	FAM184A	1.13E-25	0.47	38.25	81.71	1.00	1.00	1.00
cg24669741	1	MIR548F1;PRG4	4.063E-25	0.42	33.53	80.26	1.00	1.00	1.00
cg24674504	5	PCDHA2;PCDHA1; PCDHA1;PCDHA3; PCDHA4	2.648E-29	0.46	21.78	47.01	1.00	1.00	1.00
cg24718756	5	MARCH3	1.333E-44	2.25	33.20	14.77	1.00	1.00	1.00
cg24776427	15	MEIS2	1.329E-25	0.46	37.28	81.15	1.00	1.00	1.00
cg24792113	4	TMPRSS11E	2.667E-27	0.48	32.64	67.31	1.00	1.00	1.00
cg24817205	11	ALKBH3;LOC729799	1.863E-25	0.44	34.66	79.38	1.00	1.00	1.00
cg24826236	12	NUP37	2.427E-26	0.49	37.96	77.68	1.00	1.00	1.00
cg24867153	13	ARGLU1	2.158E-26	0.47	35.17	74.61	1.00	1.00	1.00
cg24878990	17	SMURF2	5.721E-26	0.45	33.84	75.62	1.00	1.00	1.00
cg24907762	10	ZNF22	1.801E-25	0.41	30.99	75.63	1.00	1.00	1.00
cg24918798	2	TMEM182	1.297E-25	0.42	31.86	75.67	1.00	1.00	1.00
cg24940096	10	REEP3	1.8E-26	0.43	29.02	68.03	1.00	1.00	1.00
cg24943736	17	MMD	4.044E-25	0.40	31.11	77.83	1.00	1.00	1.00
cg24946206	13	CDK8	1.207E-25	0.47	38.07	81.70	1.00	1.00	1.00
cg24947073	14	SNORD114-23; SNORD114-24	6.416E-25	0.43	36.25	84.18	1.00	1.00	1.00
cg24957532	5	TNFAIP8	2.741E-25	0.43	34.96	80.67	1.00	1.00	1.00
cg25013978	5	MIR580;LMBRD2	2.587E-28	0.49	28.59	58.32	1.00	1.00	1.00
cg25039830	8	UTP23	6.926E-27	0.49	35.81	72.62	1.00	1.00	1.00
cg25042465	8	SNTG1	2.527E-27	0.44	27.21	61.77	1.00	1.00	1.00
cg25075015	11	LRRC4C	2.963E-27	0.49	33.01	67.91	1.00	1.00	1.00
cg25114075	3	CEP63;ANAPC13	1.97E-08	2.01	15.39	7.65	1.00	1.00	1.00
cg25213141	4	SORBS2	4.274E-27	0.48	32.70	68.42	1.00	1.00	1.00
cg25235638	5	C1QTNF3	3.114E-26	0.48	37.78	78.09	1.00	1.00	1.00
cg25269222	2	HIBCH	2.585E-26	0.47	36.06	75.93	1.00	1.00	1.00
cg25290482	6	COL12A1	2.799E-26	0.49	38.76	78.82	1.00	1.00	1.00
cg25300167	2	ZAK;ZAK	1.67E-25	0.37	25.82	70.26	1.00	1.00	1.00
cg25314266	4	SCARB2	2.932E-26	0.48	36.66	76.83	1.00	1.00	1.00
cg25399162	18	BRUNOL4	7.78E-18	2.02	28.68	14.19	1.00	1.00	1.00
cg25405723	8	MTMR9	1.854E-25	0.48	41.06	85.77	1.00	1.00	1.00
cg25407540	5	C9	2.063E-27	0.49	32.14	66.26	1.00	1.00	1.00
cg25407736	1	GNG12	1.856E-26	0.48	36.49	75.58	1.00	1.00	1.00
cg25515317	7	CAV1	8.498E-25	0.39	31.31	79.99	1.00	1.00	1.00
cg25516881	2	ACADL	5.27E-25	0.40	32.27	79.68	1.00	1.00	1.00
cg25542251	5	SKIV2L2;DHX29	5.675E-46	3.15	30.64	9.74	1.00	1.00	1.00
cg25582978	6	PLEKHG1	1.623E-28	0.49	27.35	56.14	1.00	1.00	1.00
cg25619717	5	FEM1C	4.694E-26	0.46	35.85	77.15	1.00	1.00	1.00

cg25666995	7	ELMO1	5.453E-25	0.39	30.86	78.37	1.00	1.00	1.00
cg25738497	6	ANKRD6	1.824E-26	0.49	37.43	76.48	1.00	1.00	1.00
cg25744017	15	MYO5A	2.338E-30	0.39	13.44	34.23	1.00	1.00	1.00
cg25757697	12	C3AR1	5.466E-26	0.50	41.01	82.68	1.00	1.00	1.00
cg25782041	2	RPE;C2orf67	6.51E-24	0.29	22.04	76.40	1.00	1.00	1.00
cg25844216	1	ACBD3	9.871E-27	0.49	36.72	74.34	1.00	1.00	1.00
cg25878830	10	FAM190B	3.019E-23	0.27	22.16	81.08	1.00	1.00	1.00
cg25885914	12	SLCO1C1	8.932E-26	0.43	32.89	75.77	1.00	1.00	1.00
cg25911901	18	ADCYAP1	2.068E-46	2.32	38.05	16.39	1.00	1.00	1.00
cg25955565	14	SNORD56B	1.197E-25	0.47	38.66	82.26	1.00	1.00	1.00
cg25969318	7	SPAM1	2.384E-26	0.44	31.67	71.35	1.00	1.00	1.00
cg26009944	1	OSBPL9	5.122E-26	0.33	20.17	61.68	1.00	1.00	1.00
cg26076204	3	DNAH12	5.616E-26	0.50	41.59	83.32	1.00	1.00	1.00
cg26085762	2	GMCL1	8.43E-25	0.42	34.89	83.56	1.00	1.00	1.00
cg26125503	14	CTAGE5	4.394E-27	0.48	33.54	69.33	1.00	1.00	1.00
cg26166177	2	FN1	4.056E-26	0.38	25.14	66.09	1.00	1.00	1.00
cg26204042	11	RDX	3.417E-26	0.50	40.00	80.54	1.00	1.00	1.00
cg26229092	10	ZEB11	3.291E-25	0.37	27.24	73.43	1.00	1.00	1.00
cg26241746	8	ANXA13	2.152E-26	0.50	39.04	78.47	1.00	1.00	1.00
cg26269882	4	LRBA	1.39E-25	0.47	38.98	82.96	1.00	1.00	1.00
cg26284638	5	MARCH6	9.064E-47	3.81	30.20	7.93	1.00	1.00	1.00
cg26308992	4	CSN2	6.625E-25	0.38	29.04	77.06	1.00	1.00	1.00
cg26361671	1	MAN1A2	2.2E-26	0.48	37.00	76.49	1.00	1.00	1.00
cg26376025	3	KBTBD8	1.612E-26	0.39	24.92	63.68	1.00	1.00	1.00
cg26381457	12	USP44	3.467E-26	0.45	33.22	73.79	1.00	1.00	1.00
cg26417152	4	ASB5	3.903E-26	0.49	39.14	79.99	1.00	1.00	1.00
cg26464535	7	SGCE;PEG10	2.947E-42	3.24	19.99	6.16	1.00	1.00	1.00
cg26574395	19	SULT2A1	2.689E-26	0.49	38.38	78.34	1.00	1.00	1.00
cg26600826	11	OR5M1;OR8U8	8.304E-26	0.40	28.57	71.27	1.00	1.00	1.00
cg26631437	8	TEX15	1.959E-25	0.42	32.50	77.35	1.00	1.00	1.00
cg26660316	5	SLC25A46	4.046E-25	0.44	36.84	83.56	1.00	1.00	1.00
cg26687497	8	SLC30A8	1.961E-26	0.49	38.10	77.31	1.00	1.00	1.00
cg26731187	8	UBE2W	8.409E-24	0.24	17.78	72.88	1.00	1.00	1.00
cg26731897	17	C17orf90; CCDC137	1.708E-09	0.23	1.61	7.15	1.00	1.00	1.00
cg26749833	7	MIR489;CALCR; MIR653	2.928E-25	0.39	29.58	75.46	1.00	1.00	1.00
cg26828767	2	PPM1B	8.768E-26	0.48	38.78	81.61	1.00	1.00	1.00
cg26882168	4	PLK4	3.412E-26	0.44	31.51	72.04	1.00	1.00	1.00
cg26946880	5	TRIO	1.971E-43	2.72	25.62	9.42	1.00	1.00	1.00
cg26992150	4	STOX2	6.568E-25	0.38	29.16	77.15	1.00	1.00	1.00
cg27016565	15	ASB7	2.2E-47	3.01	34.90	11.61	1.00	1.00	1.00
cg27033919	11	SNORD30;SNORD29; SLC3A2;SNORD31; SNORD28;SNHG1	1.96E-44	2.74	28.52	10.40	1.00	1.00	1.00
cg27104413	3	SENP7;SENP7	3.263E-25	0.41	31.98	78.14	1.00	1.00	1.00
cg27143049	11	PDE3B;PSMA1	8.309E-17	0.30	3.87	13.05	1.00	1.00	1.00
cg27158011	15	FMN1	1.758E-26	0.47	33.90	72.86	1.00	1.00	1.00

cg27171564	12	SYT1	9.722E-27	0.43	28.48	66.07	1.00	1.00	1.00
cg27172916	1	ENAH	4.149E-26	0.46	35.30	76.30	1.00	1.00	1.00
cg27189824	16	TAT	3.692E-27	0.41	24.57	59.97	1.00	1.00	1.00
cg27200466	3	STX19;ARL13B	3.318E-25	0.41	31.63	77.83	1.00	1.00	1.00
cg27221554	8	TMEM66	1.294E-26	0.48	35.65	73.89	1.00	1.00	1.00
cg27229764	7	FAM126A	1.28E-26	0.49	36.72	74.94	1.00	1.00	1.00
cg27247225	6	C6orf41	6.507E-27	0.31	16.86	53.53	1.00	1.00	1.00
cg27280279	2	FAM98A	1.782E-27	0.49	32.65	66.45	1.00	1.00	1.00
cg27294837	13	POU4F1	1.664E-44	2.79	28.47	10.22	1.00	1.00	1.00
cg27339188	3	KAT2B	5.404E-26	0.49	39.38	81.03	1.00	1.00	1.00
cg27385729	11	DDX6	4.08E-26	0.47	36.10	77.06	1.00	1.00	1.00
cg27388035	1	BLZF1	6.579E-26	0.48	38.69	80.81	1.00	1.00	1.00
cg27413396	11	CD3G	3.566E-44	2.22	32.06	14.43	1.00	1.00	1.00
cg27431781	2	PRKRA;MIR548N	6.465E-26	0.50	41.31	83.39	1.00	1.00	1.00
cg27462988	10	DCLRE1A	3.017E-25	0.46	38.71	84.67	1.00	1.00	1.00
cg27469925	12	EEA1	2.935E-25	0.45	36.79	82.68	1.00	1.00	1.00
cg27470827	7	CACNA2D1	4.725E-26	0.41	28.72	70.04	1.00	1.00	1.00
cg27518977	7	TXNDC3	4.074E-28	0.48	28.14	58.81	1.00	1.00	1.00
cg27536084	12	LARP4	4.293E-28	0.46	25.83	56.60	1.00	1.00	1.00
cg27548142	7	CAPZA2	3.992E-26	0.42	29.49	70.40	1.00	1.00	1.00
cg27569829	18	BCL2	2.243E-28	0.39	18.56	48.00	1.00	1.00	1.00
cg27584546	10	LCOR	2.58E-26	0.46	34.48	74.34	1.00	1.00	1.00
cg27595100	20	RALGAPB	1.961E-26	0.50	38.87	78.09	1.00	1.00	1.00
cg27609489	17	KRTAP4-3	3.255E-26	0.48	37.44	77.85	1.00	1.00	1.00
cg27645544	12	SLC5A8	2.367E-27	0.44	27.03	61.44	1.00	1.00	1.00
cg27657169	5	ZSWIM6	3.067E-25	0.44	35.94	81.94	1.00	1.00	1.00
ch.1.1158789F	1	EIF2C3	3.024E-49	2.35	45.74	19.51	1.00	1.00	1.00
ch.11.962980R	11	KIAA0652	9.737E-44	2.12	31.82	15.03	1.00	1.00	1.00
ch.15.403279R	15	CASC5	2.398E-45	2.88	30.31	10.51	1.00	1.00	1.00
ch.20.396516F	20	DTD1	6.775E-48	2.27	43.12	18.99	1.00	1.00	1.00
ch.5.2533981R	5	FAM13B	5.061E-53	4.55	40.46	8.89	1.00	1.00	1.00
ch.5.2607995R	5	HARS	1.935E-17	2.55	20.49	8.02	1.00	1.00	1.00
cg00338852	3	ARL13B	2.782E-27	0.44	27.32	62.09	0.99	0.97	1.00
cg00377332	3	SACM1L	9.544E-26	0.46	37.22	80.26	0.99	0.97	1.00
cg00397714	2	TMEFF2	1.125E-25	0.42	31.45	74.90	0.99	0.97	1.00
cg00487533	2	C2orf3	5.751E-26	0.45	33.93	75.73	0.99	0.97	1.00
cg00599328	7	PEX1	6.398E-27	0.45	30.36	66.99	0.99	0.97	1.00
cg00614011	8	NOV	2.578E-25	0.41	31.12	76.67	0.99	0.97	1.00
cg00750399	17	HELZ	5.419E-26	0.45	33.93	75.58	0.99	0.97	1.00
cg00879541	14	C14orf166	4.757E-27	0.48	32.77	68.74	0.99	0.97	1.00
cg01074676	14	C14orf135	1.133E-29	0.44	18.71	42.35	0.99	0.97	1.00
cg01293346	12	TAS2R20;PRR4;<br>PRH1	2.27E-28	0.45	24.05	53.51	0.99	0.97	1.00
cg01298374	12	PPFIBP1	1.766E-26	0.45	32.07	71.04	0.99	0.97	1.00
cg01364129	1	MAGI3	1.832E-26	0.50	38.52	77.58	0.99	0.97	1.00
cg01436723	11	NOX4	1.889E-26	0.46	33.79	72.92	0.99	0.97	1.00
cg01581024	8	PTK2	7.26E-26	0.46	36.77	79.14	0.99	0.97	1.00
cg01775802	14	RGS6	1.338E-31	0.20	4.00	19.92	0.99	0.97	1.00

cg01835854	4	C4orf41	1.083E-26	0.50	37.57	75.40	0.99	0.97	1.00
cg01893571	2	FAM82A1	3.478E-26	0.43	30.67	71.25	0.99	0.97	1.00
cg02395581	7	ASB4;ASB4	3.499E-26	0.41	28.21	68.80	0.99	0.97	1.00
cg02583841	19	ZNF714	1.178E-27	0.44	25.63	58.53	0.99	0.97	1.00
cg02673114	1	ALG14	1.637E-43	2.81	25.37	9.01	0.99	0.97	1.00
cg02675047	14	SNORD113-2	4.297E-26	0.49	38.80	79.89	0.99	0.97	1.00
cg02976359	3	CHL1	2.208E-26	0.48	35.95	75.45	0.99	0.97	1.00
cg03028506	15	SPRED1	9.13E-26	0.47	37.75	80.68	0.99	0.97	1.00
cg03108373	7	ZNF804B	7.096E-27	0.43	27.44	64.31	0.99	0.97	1.00
cg03128219	10	C10orf128	2.385E-27	0.45	27.98	62.41	0.99	0.97	1.00
cg03305454	3	HIGD1A	1.188E-25	0.47	39.14	82.72	0.99	0.97	1.00
cg03349373	15	TARSL2	1.231E-25	0.47	38.80	82.48	0.99	0.97	1.00
cg03526614	3	ACTL6A	4.964E-29	0.49	25.33	51.77	0.99	0.97	1.00
cg03605164	4	KCTD8	7.985E-25	0.33	23.70	72.22	0.99	0.97	1.00
cg03699633	11	PGM2L1	1.176E-24	0.43	36.70	86.26	0.99	0.97	1.00
cg03836638	4	MARCH1	1.955E-26	0.49	38.11	77.32	0.99	0.97	1.00
cg04471701	6	LCA5	5.169E-25	0.45	39.31	86.68	0.99	0.97	1.00
cg04477962	12	METTL7A	2.247E-26	0.41	27.25	66.78	0.99	0.97	1.00
cg04588051	11	SBF2	2.752E-26	0.49	39.07	79.09	0.99	0.97	1.00
cg04799778	3	EIF5A2	3.943E-27	0.42	25.49	61.03	0.99	0.97	1.00
cg04989278	1	PDE4B	1.414E-45	2.25	36.40	16.20	0.99	0.97	1.00
cg05262335	6	TAGAP	7.52E-46	2.11	39.26	18.57	0.99	0.97	1.00
cg05479657	12	TMEM132D	2.526E-25	0.44	35.19	80.69	0.99	0.97	1.00
cg05648614	3	ARL8B	3.372E-26	0.42	29.59	70.10	0.99	0.97	1.00
cg05702774	3	SUCNR1	4.073E-30	0.44	17.22	38.99	0.99	0.97	1.00
cg05838627	10	RPS24	1.8E-26	0.49	37.78	76.79	0.99	0.97	1.00
cg05897853	3	CLDND1	2.861E-26	0.45	32.84	72.96	0.99	0.97	1.00
cg05982274	12	AMDHD1	1.788E-25	0.43	33.60	78.21	0.99	0.97	1.00
cg06022439	6	HCG18	2.6E-25	0.41	31.76	77.33	0.99	0.97	1.00
cg06065089	4	BMPR1B	1.527E-25	0.44	34.33	78.54	0.99	0.97	1.00
cg06250693	11	JAM3	2.13E-15	0.49	11.46	23.59	0.99	0.97	1.00
cg06291266	16	NUDT21	1.191E-26	0.46	32.66	70.72	0.99	0.97	1.00
cg06492821	16	AKTIP	1.993E-12	0.40	5.56	13.90	0.99	0.97	1.00
cg06573795	5	AGGF1	4.218E-26	0.46	35.17	76.22	0.99	0.97	1.00
cg06669598	6	ECHDC1	4.982E-27	0.46	30.31	66.37	0.99	0.97	1.00
cg06736434	19	ZNF90	7.618E-25	0.37	28.94	77.33	0.99	0.97	1.00
cg07062338	10	FGFR2	5.352E-26	0.45	34.39	76.01	0.99	0.97	1.00
cg07347276	11	PDHX	1.287E-26	0.49	36.57	74.81	0.99	0.97	1.00
cg07491828	16	MKL2	2.615E-26	0.48	36.88	76.78	0.99	0.97	1.00
cg07592830	1	NEK7	3.496E-26	0.46	34.48	75.07	0.99	0.97	1.00
cg07739398	12	SOX5;MIR920	8.359E-26	0.42	30.95	73.66	0.99	0.97	1.00
cg07755760	3	DLG1	8.716E-25	0.39	31.48	80.23	0.99	0.97	1.00
cg08099418	13	INTS6	2.108E-26	0.49	38.07	77.46	0.99	0.97	1.00
cg08348900	6	VIP	4.927E-43	2.03	30.38	14.98	0.99	0.97	1.00
cg08377570	13	LPAR6;RB1	1.085E-27	0.45	27.02	59.75	0.99	0.97	1.00
cg08383526	8	ADAM7	9.381E-27	0.47	33.10	70.61	0.99	0.97	1.00
cg08534269	3	PLOD2	5.216E-27	0.49	35.05	71.22	0.99	0.97	1.00
cg08544041	1	GLRX2	7.793E-31	0.37	10.90	29.77	0.99	0.97	1.00

cg08681919	6	FUT9	9.496E-27	0.49	36.59	74.13	0.99	0.97	1.00
cg09202227	16	SMPD3	6.23E-15	0.38	5.80	15.26	0.99	0.97	1.00
cg09295792	14	L2HGDH	9.493E-26	0.46	36.83	79.86	0.99	0.97	1.00
cg09646593	4	ANKRD50	2.16E-25	0.48	42.11	87.21	0.99	0.97	1.00
cg09805271	11	MMP26	1.877E-27	0.48	31.87	65.78	0.99	0.97	1.00
cg09898988	5	FAM173B	2.016E-24	0.35	27.96	79.00	0.99	0.97	1.00
cg10451262	8	ZHX1	5.151E-24	0.37	30.89	84.57	0.99	0.97	1.00
cg10469659	15	SPPL2A	1.734E-22	0.44	12.76	28.67	0.99	0.97	1.00
cg10519882	5	RNF130	4.106E-26	0.48	37.81	78.78	0.99	0.97	1.00
cg10672201	12	ASCL1	5.449E-49	2.13	48.65	22.80	0.99	0.97	1.00
cg10819004	8	NKAIN3	1.492E-27	0.50	33.19	66.61	0.99	0.97	1.00
cg11182257	10	KIAA1598	1.02E-45	3.00	30.70	10.25	0.99	0.97	1.00
cg11322936	10	MARCH8;	1.591E-26	0.50	38.11	76.83	0.99	0.97	1.00
cg11329058	17	RPTOR	2.593E-48	2.47	41.66	16.87	0.99	0.97	1.00
cg11806762	11	BDNF	3.679E-26	0.44	31.44	72.15	0.99	0.97	1.00
cg11911128	13	MYCBP2	1.737E-27	0.48	31.71	65.45	0.99	0.97	1.00
cg12170649	12	APPL2	5.813E-28	0.48	28.88	60.28	0.99	0.97	1.00
cg12308110	7	STEAP4	1.532E-26	0.47	34.20	72.84	0.99	0.97	1.00
cg12456379	5	HISPPD1	8.822E-26	0.43	32.18	75.03	0.99	0.97	1.00
cg12522722	11	ZW10	1.081E-25	0.48	40.34	83.69	0.99	0.97	1.00
cg12565580	13	ATP8A2	7.193E-26	0.37	25.18	67.52	0.99	0.97	1.00
cg12657739	1	HIPK1	1.461E-25	0.47	39.40	83.51	0.99	0.97	1.00
cg13042575	12	RASSF3	1.124E-42	2.03	28.91	14.23	0.99	0.97	1.00
cg13043598	11	SNORA32;SNORA25	1.571E-25	0.35	23.70	67.99	0.99	0.97	1.00
cg13177421	12	EPS8	2.229E-27	0.50	33.76	68.05	0.99	0.97	1.00
cg13237740	11	OR10A6	9.783E-28	0.46	27.71	60.21	0.99	0.97	1.00
cg13362491	6	PKHD1	6.426E-26	0.47	37.33	79.39	0.99	0.97	1.00
cg13726218	13	DACH1	2.753E-45	2.34	34.41	14.72	0.99	0.97	1.00
cg13800569	6	UBE2CBP	6.367E-26	0.45	35.09	77.14	0.99	0.97	1.00
cg13827458	14	ERO1L	5.482E-26	0.47	36.69	78.37	0.99	0.97	1.00
cg14010852	4	NPFFR2	1.133E-27	0.49	31.80	64.62	0.99	0.97	1.00
cg14420613	2	NBEAL1	4.939E-25	0.34	24.86	72.10	0.99	0.97	1.00
cg14430499	15	TBC1D21	1.681E-48	2.14	47.16	22.07	0.99	0.97	1.00
cg14869517	15	GALK2	9.824E-27	0.50	37.38	74.99	0.99	0.97	1.00
cg14881187	7	DMTF1	1.124E-26	0.45	31.56	69.48	0.99	0.97	1.00
cg15057359	2	LRRTM4	5.914E-26	0.39	26.41	68.28	0.99	0.97	1.00
cg15160274	5	SGCD	8.176E-28	0.45	25.89	58.01	0.99	0.97	1.00
cg15255329	19	LAIR2	8.498E-27	0.43	28.50	65.78	0.99	0.97	1.00
cg15361251	4	WWC2	1.252E-27	0.49	31.28	64.31	0.99	0.97	1.00
cg15496430	1	FOXJ3	2.546E-25	0.45	37.30	82.81	0.99	0.97	1.00
cg15626828	1	PTPRC	1.632E-49	2.59	43.37	16.73	0.99	0.97	1.00
cg15695478	3	SYN2	4.057E-26	0.45	33.04	73.99	0.99	0.97	1.00
cg15785147	3	UBE2E2	5.379E-26	0.49	39.76	81.39	0.99	0.97	1.00
cg16095464	12	SOX5	5.44E-26	0.38	25.96	67.61	0.99	0.97	1.00
cg16420520	6	NT5DC1	8.307E-26	0.46	35.86	78.56	0.99	0.97	1.00
cg16454107	17	TMEM100	1.349E-27	0.50	32.64	65.84	0.99	0.97	1.00
cg16741709	14	MPP5	9.126E-44	2.46	28.40	11.55	0.99	0.97	1.00
cg17213048	8	ATAD2	6.816E-26	0.43	31.50	73.70	0.99	0.97	1.00

cg17525418	2	ACTR2	1.213E-25	0.49	42.32	85.96	0.99	0.97	1.00
cg17736116	15	ZNF280D	1.812E-24	0.36	29.13	79.87	0.99	0.97	1.00
cg17869167	2	IL18R1	5.242E-27	0.40	23.90	60.08	0.99	0.97	1.00
cg17878273	12	NELL2	9.8E-26	0.44	33.73	76.83	0.99	0.97	1.00
cg18069705	4	HSPA4L	8.145E-27	0.47	33.23	70.41	0.99	0.97	1.00
cg18143869	3	MED12L;P2RY12	6.27E-26	0.43	31.36	73.36	0.99	0.97	1.00
cg18159684	8	AZIN1	2.186E-26	0.42	28.35	67.83	0.99	0.97	1.00
cg18220168	6	DSE	8.375E-27	0.48	33.87	71.11	0.99	0.97	1.00
cg18286127	10	C10orf84;C10orf84	8.261E-25	0.41	34.06	82.67	0.99	0.97	1.00
cg18876189	4	FGB	1.641E-25	0.42	32.69	77.08	0.99	0.97	1.00
cg18941085	14	DDX24;IFI27L1	8.391E-12	0.42	6.09	14.39	0.99	0.97	1.00
cg18954925	4	UBE2D3	3.516E-26	0.48	37.24	77.85	0.99	0.97	1.00
cg19136783	4	LDB2	2.23E-26	0.47	34.97	74.49	0.99	0.97	1.00
cg19393323	2	FIGN	1.268E-25	0.43	33.62	77.36	0.99	0.97	1.00
cg19475870	5	CDH9	5.033E-28	0.48	28.40	59.51	0.99	0.97	1.00
cg19820921	3	ATP11B	6.133E-26	0.39	27.15	69.10	0.99	0.97	1.00
cg19892525	11	ATM	3.764E-29	0.46	22.10	48.01	0.99	0.97	1.00
cg20062978	6	NUP153	1.085E-26	0.48	34.38	72.21	0.99	0.97	1.00
cg20106465	10	GPAM	9.083E-28	0.47	28.64	60.99	0.99	0.97	1.00
cg20915333	7	AHCYL2	1.928E-26	0.48	36.29	75.47	0.99	0.97	1.00
cg21232615	9	C9orf11	1.196E-25	0.47	39.02	82.63	0.99	0.97	1.00
cg21411962	1	KIAA0562	8.743E-27	0.50	36.88	74.22	0.99	0.97	1.00
cg21649520	8	PMP2	1.646E-25	0.40	29.43	73.84	0.99	0.97	1.00
cg21870038	17	RFFL	5.923E-14	2.36	18.58	7.86	0.99	0.97	1.00
cg22074858	1	GBP3	4.267E-49	2.75	40.90	14.90	0.99	0.97	1.00
cg22103831	13	PCDH17	7.142E-26	0.33	21.29	63.62	0.99	0.97	1.00
cg22332577	8	WRN	5.932E-26	0.45	33.97	75.84	0.99	0.97	1.00
cg22410743	15	IQCH	8.611E-27	0.34	18.87	56.18	0.99	0.97	1.00
cg22457860	12	PTPRR	1.219E-25	0.40	28.91	72.56	0.99	0.97	1.00
cg22466716	5	DNAH5	1.758E-25	0.45	36.75	81.32	0.99	0.97	1.00
cg22693266	12	CCDC91	1.246E-26	0.50	38.10	76.26	0.99	0.97	1.00
cg22803222	6	IMPG1	7.305E-27	0.47	33.34	70.28	0.99	0.97	1.00
cg22925553	4	ELMOD2	4.372E-26	0.49	39.63	80.76	0.99	0.97	1.00
cg23013489	10	C10orf79	1.452E-26	0.49	36.93	75.44	0.99	0.97	1.00
cg23068345	6	PPP1R10	8.419E-44	2.34	29.52	12.61	0.99	0.97	1.00
cg23270582	4	UGT2A1	2.416E-26	0.50	39.09	78.80	0.99	0.97	1.00
cg23521294	13	ARHGEF7	1.043E-26	0.48	34.45	72.19	0.99	0.97	1.00
cg23834427	11	DNHD1	2.127E-26	0.49	37.49	76.90	0.99	0.97	1.00
cg23970740	3	TNIK	4.316E-25	0.43	36.01	82.90	0.99	0.97	1.00
cg24458328	7	OR2A1	1.356E-25	0.46	37.37	81.29	0.99	0.97	1.00
cg24495234	15	CTXN2	3.238E-45	2.21	35.78	16.22	0.99	0.97	1.00
cg25107282	12	SLC41A2	6.898E-27	0.49	35.20	72.00	0.99	0.97	1.00
cg25246876	13	KLHL1	3.44E-27	0.49	33.37	68.61	0.99	0.97	1.00
cg25287153	1	NEXN	1.805E-25	0.39	28.37	73.01	0.99	0.97	1.00
cg26023902	6	LOC285735	3.254E-26	0.45	32.56	72.97	0.99	0.97	1.00
cg26026748	1	PBX1	8.731E-14	2.03	23.00	11.32	0.99	0.97	1.00
cg26064247	11	FBXO3	3.641E-26	0.43	31.29	71.98	0.99	0.97	1.00
cg26186249	2	MOGAT1	4.208E-25	0.40	30.98	77.81	0.99	0.97	1.00

cg26357596	5	GZMA	4.207E-42	2.34	23.60	10.10	0.99	0.97	1.00
cg26403975	4	OSTC	3.931E-14	2.59	16.85	6.52	0.99	0.97	1.00
cg26430059	14	FOXN3	2.072E-14	2.71	16.30	6.02	0.99	0.97	1.00
cg26961824	2	SGOL2	7.754E-28	0.44	25.09	57.10	0.99	0.97	1.00
cg27284627	11	NTM	3.342E-28	0.48	28.39	58.64	0.99	0.97	1.00
cg27386033	7	ABCA13	1.091E-25	0.47	37.71	81.08	0.99	0.97	1.00
cg27563778	4	C4orf17	3.446E-27	0.49	34.37	69.61	0.99	0.97	1.00
ch.11.1834293R	11	PICALM	1.133E-12	2.53	15.76	6.22	0.99	0.97	1.00
ch.22.441164F	22	DEPDC5	1.647E-47	3.83	31.79	8.29	0.99	0.97	1.00
cg00495811	16	LPCAT2	8.602E-26	0.45	34.86	77.64	0.99	0.96	1.00
cg00771217	4	TMPRSS11D	3.025E-26	0.44	31.01	71.25	0.99	0.96	1.00
cg00973737	4	ADH6	1.034E-26	0.49	36.11	73.83	0.99	0.96	1.00
cg01260541	6	MAN1A1	3.463E-27	0.47	31.41	66.66	0.99	0.96	1.00
cg01664065	13	ITGBL1	8.162E-26	0.45	34.40	77.06	0.99	0.96	1.00
cg01864699	6	SERINC1	3.661E-26	0.42	29.58	70.28	0.99	0.96	1.00
cg02051562	1	APOBEC4;RGL1	3.4E-26	0.46	34.72	75.24	0.99	0.96	1.00
cg02130329	8	ANGPT1	1.004E-25	0.43	32.55	75.72	0.99	0.96	1.00
cg02769668	1	RPL5;SNORD21	4.729E-26	0.47	36.30	77.61	0.99	0.96	1.00
cg03193505	8	DPYS	1.233E-25	0.43	32.63	76.31	0.99	0.96	1.00
cg03322752	7	SEC61G	5.029E-26	0.47	37.48	78.95	0.99	0.96	1.00
cg03462556	10	SLC16A9	1.125E-25	0.50	43.04	86.49	0.99	0.96	1.00
cg03981213	2	BCL11A	1.719E-26	0.49	36.95	75.86	0.99	0.96	1.00
cg04250926	1	CRYZ;TYW3	5.817E-46	2.33	36.62	15.74	0.99	0.96	1.00
cg04840732	8	MTMR7	3.834E-26	0.45	33.50	74.31	0.99	0.96	1.00
cg04866357	15	SNORD18C;RPL4;<br>SNORD18B	5.387E-27	0.49	34.29	70.53	0.99	0.96	1.00
cg04940312	11	TRIM44	7.57E-27	0.46	31.84	68.86	0.99	0.96	1.00
cg04968197	6	GNL1	1.144E-16	2.54	19.67	7.73	0.99	0.96	1.00
cg05194545	10	ARHGAP12	5.63E-26	0.45	33.70	75.44	0.99	0.96	1.00
cg05514531	3	GBE1	1.311E-26	0.44	30.36	68.63	0.99	0.96	1.00
cg05593615	8	KCTD9	1.665E-27	0.39	21.54	55.19	0.99	0.96	1.00
cg05874478	14	RNF31;PSME2	6.43E-25	0.45	14.99	32.97	0.99	0.96	1.00
cg05945984	8	ADAM9	2.114E-26	0.49	37.68	77.08	0.99	0.96	1.00
cg06393354	1	OR2M3	6.705E-28	0.49	31.03	62.73	0.99	0.96	1.00
cg06483441	5	GIN1	1.174E-25	0.37	25.33	68.89	0.99	0.96	1.00
cg06546571	10	PARD3	5.436E-27	0.50	36.10	72.36	0.99	0.96	1.00
cg06749213	14	OR4K5	8.984E-27	0.49	36.60	74.00	0.99	0.96	1.00
cg06764152	6	JARID2	1.632E-26	0.48	35.55	74.34	0.99	0.96	1.00
cg07120254	6	TNFRSF21	7.467E-28	0.49	31.26	63.19	0.99	0.96	1.00
cg07180674	11	DCUN1D5	1.082E-25	0.45	35.81	79.17	0.99	0.96	1.00
cg07249860	13	MIR548F5;DCLK1	4.979E-27	0.45	28.92	64.99	0.99	0.96	1.00
cg07522639	4	DHX15	4.905E-27	0.46	31.16	67.19	0.99	0.96	1.00
cg08363193	10	PTEN	5.133E-27	0.45	29.62	65.76	0.99	0.96	1.00
cg08751994	7	DYNC1I1	2.604E-26	0.44	31.35	71.23	0.99	0.96	1.00
cg08761490	7	MEST;MIR335	8.512E-26	0.47	37.89	80.64	0.99	0.96	1.00
cg08860443	6	PTPRK	7.849E-27	0.48	34.50	71.59	0.99	0.96	1.00
cg09171882	12	GLIPR1	1.569E-46	3.27	31.50	9.63	0.99	0.96	1.00
cg09236780	13	MYO16	1.209E-24	0.46	42.70	92.34	0.99	0.96	1.00

cg09453983	4	OTUD4	1.579E-25	0.45	36.98	81.28	0.99	0.96	1.00
cg09476325	10	ITGB1	1.422E-26	0.50	38.30	76.77	0.99	0.96	1.00
cg09636727	1	ST6GALNAC5	1.826E-27	0.49	32.59	66.43	0.99	0.96	1.00
cg09747829	6	BAI3	2.159E-46	2.49	36.10	14.47	0.99	0.96	1.00
cg09926562	15	FANCI	2.151E-26	0.46	33.93	73.36	0.99	0.96	1.00
cg09928300	14	COCH	5.376E-27	0.49	35.12	71.36	0.99	0.96	1.00
cg10211454	13	USPL1	3.886E-27	0.46	29.94	65.45	0.99	0.96	1.00
cg10369203	6	HIVEP2	2.001E-25	0.34	23.37	68.27	0.99	0.96	1.00
cg10379992	13	RNF17	2.908E-25	0.37	26.66	72.52	0.99	0.96	1.00
cg10806639	13	STARD13	2.637E-50	2.87	42.70	14.89	0.99	0.96	1.00
cg10827090	21	USP25	2.08E-27	0.48	31.17	65.31	0.99	0.96	1.00
cg10853431	10	USP6NL	1.285E-26	0.47	34.39	72.62	0.99	0.96	1.00
cg10938899	13	GTF2F2	6.498E-25	0.34	25.17	73.13	0.99	0.96	1.00
cg11155374	13	FREM2	7.663E-27	0.49	35.41	72.45	0.99	0.96	1.00
cg11367404	4	SH3RF1	1.004E-25	0.42	31.85	75.02	0.99	0.96	1.00
cg11661265	4	ARAP2	7.149E-26	0.47	36.94	79.26	0.99	0.96	1.00
cg11690826	1	ZCCHC11	1.427E-26	0.49	36.41	74.88	0.99	0.96	1.00
cg11822088	1	CEP170	1.623E-26	0.48	36.35	75.12	0.99	0.96	1.00
cg11963436	10	TNKS2	2.417E-14	0.44	8.21	18.52	0.99	0.96	1.00
cg12171875	3	PDE12	8.113E-27	0.46	32.10	69.27	0.99	0.96	1.00
cg12331980	2	CCDC141	6.531E-26	0.46	36.43	78.54	0.99	0.96	1.00
cg12367789	2	STK39	1.943E-26	0.50	39.11	78.30	0.99	0.96	1.00
cg12486814	1	C1orf192	1.614E-45	2.28	35.75	15.65	0.99	0.96	1.00
cg12659933	1	ST7L	7.358E-26	0.41	29.44	71.84	0.99	0.96	1.00
cg12781778	6	ARID1B	5.445E-43	2.34	26.73	11.41	0.99	0.96	1.00
cg13096721	1	FAF1	3.127E-25	0.40	30.85	76.90	0.99	0.96	1.00
cg13137458	11	PAK1;PAK1	6.026E-46	2.77	32.61	11.75	0.99	0.96	1.00
cg13802433	2	PNO1	3.044E-27	0.49	34.01	68.98	0.99	0.96	1.00
cg14152613	8	FABP4	1.956E-25	0.43	33.94	78.79	0.99	0.96	1.00
cg14340103	4	IL21	3.659E-27	0.48	32.55	67.93	0.99	0.96	1.00
cg14963812	3	CAPN7	1.81E-26	0.49	37.15	76.18	0.99	0.96	1.00
cg14995416	3	IL12A	1.798E-26	0.41	27.46	66.48	0.99	0.96	1.00
cg15236063	3	SELK	1.361E-27	0.49	32.19	65.40	0.99	0.96	1.00
cg15444076	6	NDUFAF4	1.574E-44	2.51	30.40	12.11	0.99	0.96	1.00
cg15447825	13	CUL4A	8.252E-29	0.49	26.89	54.33	0.99	0.96	1.00
cg15566921	13	PCDH9	2.033E-24	0.35	27.52	78.58	0.99	0.96	1.00
cg15858483	20	CTCFL	3.929E-44	2.53	29.01	11.46	0.99	0.96	1.00
cg15936990	1	WASF2	1.199E-42	2.51	24.33	9.70	0.99	0.96	1.00
cg16679068	8	CDH17	6.549E-44	3.06	25.45	8.33	0.99	0.96	1.00
cg17148580	8	TTC35	1.172E-25	0.42	31.52	75.07	0.99	0.96	1.00
cg17479576	4	FAM160A1	5.885E-27	0.45	29.30	65.74	0.99	0.96	1.00
cg17664443	3	IL1RAP	8.319E-27	0.45	31.00	68.23	0.99	0.96	1.00
cg17707057	7	STK17A	1.459E-47	2.49	39.42	15.83	0.99	0.96	1.00
cg17784027	8	ATP6V0D2	8.372E-27	0.49	36.17	73.41	0.99	0.96	1.00
cg17836145	6	VNN2	5.388E-47	2.47	38.01	15.36	0.99	0.96	1.00
cg17930169	14	SNORD113-7	3.558E-26	0.45	33.33	73.96	0.99	0.96	1.00
cg17951408	7	IQUB	1.015E-24	0.37	29.43	78.59	0.99	0.96	1.00
cg19268947	7	PTPN12	2.179E-26	0.31	18.11	57.57	0.99	0.96	1.00

cg19309752	1	VTCN1	5.644E-27	0.47	32.32	68.67	0.99	0.96	1.00
cg19592431	15	CEP152	2.605E-25	0.45	37.60	83.18	0.99	0.96	1.00
cg19623877	6	MYB	5.297E-13	2.23	18.98	8.52	0.99	0.96	1.00
cg19761245	4	LIMCH1	5.437E-28	0.24	9.99	41.26	0.99	0.96	1.00
cg19857379	1	CLIC4	8.726E-28	0.46	27.22	59.48	0.99	0.96	1.00
cg19981475	12	ACSS3	1.332E-26	0.50	37.76	76.08	0.99	0.96	1.00
cg20080079	12	TSPAN19;LRRIQ1	1.101E-43	2.96	25.21	8.52	0.99	0.96	1.00
cg20308663	13	CLYBL	4.017E-26	0.49	39.86	80.79	0.99	0.96	1.00
cg20697188	5	TMEM167A; SCARNA18	1.102E-25	0.46	36.86	80.26	0.99	0.96	1.00
cg20956906	12	BICD1	4.027E-26	0.44	32.31	73.24	0.99	0.96	1.00
cg21188454	9	TBC1D13	8.973E-14	0.27	2.84	10.45	0.99	0.96	1.00
cg21204600	6	HULC	1.659E-26	0.43	29.85	68.68	0.99	0.96	1.00
cg21245981	5	SLC1A3	1.283E-16	2.24	23.01	10.27	0.99	0.96	1.00
cg21404906	2	XRCC5	1.26E-14	2.38	19.18	8.07	0.99	0.96	1.00
cg21549437	15	PIAS1	5.643E-27	0.50	35.94	72.29	0.99	0.96	1.00
cg21775854	2	C2orf69	1.126E-25	0.44	33.78	77.23	0.99	0.96	1.00
cg22010963	6	C6orf165	5.023E-26	0.48	38.40	79.86	0.99	0.96	1.00
cg22129614	6	ZFAND3	1.03E-26	0.48	35.43	73.15	0.99	0.96	1.00
cg22166248	2	NXPH2	3.641E-27	0.50	35.22	70.59	0.99	0.96	1.00
cg22539390	13	TGDS	1.22E-24	0.37	28.80	78.46	0.99	0.96	1.00
cg22549853	15	COPS2	6.887E-26	0.44	32.60	74.84	0.99	0.96	1.00
cg22559630	7	CTTNBP2	1.015E-25	0.44	33.69	76.89	0.99	0.96	1.00
cg22692868	5	PCDHGA4; PCDHGA9	8.733E-27	0.50	37.14	74.48	0.99	0.96	1.00
cg23159678	14	NOVA1	1.686E-25	0.41	31.01	75.48	0.99	0.96	1.00
cg23194644	6	CYB5R4	2.763E-25	0.40	30.79	76.52	0.99	0.96	1.00
cg23258033	12	THAP2	1.178E-25	0.41	30.74	74.31	0.99	0.96	1.00
cg23530064	21	BACH1	4.187E-27	0.48	32.85	68.53	0.99	0.96	1.00
cg24192786	5	CSNK1G3	6.87E-28	0.47	28.41	60.17	0.99	0.96	1.00
cg24636611	6	REPS1	4.505E-26	0.43	30.57	71.77	0.99	0.96	1.00
cg24948782	17	UTP18	2.407E-27	0.36	19.67	54.13	0.99	0.96	1.00
cg25009327	7	ZNF425	1.362E-13	0.43	7.01	16.46	0.99	0.96	1.00
cg25461513	11	CADM1	4.971E-27	0.45	29.69	65.76	0.99	0.96	1.00
cg25890048	11	OR5I1	3.751E-27	0.49	34.14	69.57	0.99	0.96	1.00
cg26286098	13	B3GALTL	3.09E-26	0.47	35.20	75.50	0.99	0.96	1.00
cg26457248	18	MBP	1.424E-45	2.41	34.51	14.31	0.99	0.96	1.00
cg26860848	1	ZRANB2	2.659E-26	0.49	38.95	78.89	0.99	0.96	1.00
cg27099277	6	BRP44L	9.57E-27	0.49	35.70	73.25	0.99	0.96	1.00
cg27124774	5	PCDHB10	9.472E-52	2.40	51.14	21.28	0.99	0.96	1.00
cg27519599	8	PKHD1L1	5.022E-26	0.42	30.32	71.78	0.99	0.96	1.00
ch.16.406779R	16	CLEC16A	2.108E-42	2.75	22.21	8.08	0.99	0.96	1.00
ch.20.577701F	20	HM13	5.017E-44	2.55	28.51	11.16	0.99	0.96	1.00
ch.4.255198F	4	TBC1D14	2.536E-46	2.32	37.86	16.35	0.99	0.96	1.00
cg00222125	13	SUGT1	5.278E-47	2.51	37.63	14.96	0.98	0.95	1.00
cg00869533	2	VRK2;FANCL	8.634E-28	0.44	25.37	57.61	0.98	0.95	1.00
cg01613401	2	CTNNA2	8.128E-26	0.37	25.09	67.73	0.98	0.95	1.00
cg01824625	3	EPHA3	4.381E-26	0.45	33.95	75.08	0.98	0.95	1.00

cg02182794	4	RAB28	9.087E-28	0.49	30.56	62.91	0.98	0.95	1.00
cg02632362	1	EDARADD	2.731E-51	2.35	50.79	21.57	0.98	0.95	1.00
cg02633036	15	SLCO3A1	1.302E-25	0.45	35.24	79.06	0.98	0.95	1.00
cg02835561	1	CNN3	1.493E-26	0.48	36.14	74.72	0.98	0.95	1.00
cg03129732	4	KLHL8	5.647E-27	0.48	33.40	69.75	0.98	0.95	1.00
cg03240800	5	PCDHA6;PCDHA2; PCDHA1;PCDHA7; PCDHA8;PCDHA5; PCDHA3;PCDHA4	6.672E-26	0.38	26.38	68.54	0.98	0.95	1.00
cg03375703	2	BAZ2B	6.755E-27	0.45	30.15	66.91	0.98	0.95	1.00
cg03656483	22	EP300	5.725E-44	3.23	24.96	7.73	0.98	0.95	1.00
cg03861633	3	SLC7A14	2.696E-26	0.43	30.08	70.05	0.98	0.95	1.00
cg04231905	15	MCTP2	1.52E-16	2.35	21.48	9.14	0.98	0.95	1.00
cg04270835	11	SLC17A6	6.572E-46	2.58	33.95	13.16	0.98	0.95	1.00
cg04416247	3	CADM2	4.789E-26	0.42	29.39	70.74	0.98	0.95	1.00
cg04492287	6	HIST1H2BD	8.481E-28	0.48	30.00	62.20	0.98	0.95	1.00
cg04688351	2	PAX3	1.186E-46	3.55	30.72	8.65	0.98	0.95	1.00
cg05737526	18	TGIF1	3.999E-42	2.53	22.42	8.87	0.98	0.95	1.00
cg06089463	4	C4orf31	2.776E-26	0.41	27.80	67.84	0.98	0.95	1.00
cg06576021	8	SNAI2	3.255E-26	0.41	28.13	68.54	0.98	0.95	1.00
cg07406888	6	KLHL31	1.723E-25	0.45	35.88	80.40	0.98	0.95	1.00
cg07547798	8	NSMCE2	3.93E-26	0.45	33.27	74.14	0.98	0.95	1.00
cg07864632	2	TRIP12	7.143E-15	2.20	21.61	9.83	0.98	0.95	1.00
cg08002765	12	LEMD3	7.972E-27	0.47	33.17	70.30	0.98	0.95	1.00
cg09581197	16	CFDP1	1.12E-26	0.49	35.86	73.77	0.98	0.95	1.00
cg10514237	2	TANK	1.92E-25	0.42	32.76	77.55	0.98	0.95	1.00
cg10523193	11	SNORA25	9.553E-28	0.34	16.61	49.06	0.98	0.95	1.00
cg10884539	6	MYLK4	2.069E-44	2.92	27.46	9.39	0.98	0.95	1.00
cg11253592	11	MAML2	2.406E-27	0.48	32.26	66.71	0.98	0.95	1.00
cg11356706	20	SCRT2	3.447E-07	0.41	3.64	8.97	0.98	0.95	1.00
cg11448270	6	FRK;LOC728402	2.111E-26	0.47	34.97	74.36	0.98	0.95	1.00
cg11571304	6	TRIM38	1.799E-42	2.51	23.74	9.47	0.98	0.95	1.00
cg13289463	2	RBMS1	3.564E-27	0.48	32.60	67.92	0.98	0.95	1.00
cg13328485	2	UBR3	9.583E-26	0.39	27.51	70.56	0.98	0.95	1.00
cg13488020	4	SEC24D	1.514E-25	0.45	36.30	80.49	0.98	0.95	1.00
cg13858975	15	LYSMD2	1.407E-26	0.48	35.87	74.31	0.98	0.95	1.00
cg14286594	4	NAA15	6.275E-28	0.46	26.46	58.02	0.98	0.95	1.00
cg14564351	6	AHI1	1.155E-49	2.41	45.90	19.03	0.98	0.95	1.00
cg15294851	2	DYSF	7.506E-43	2.51	25.01	9.97	0.98	0.95	1.00
cg15336091	6	HIST1H4B	2.041E-26	0.45	32.47	71.78	0.98	0.95	1.00
cg15506863	14	C14orf23	8.03E-28	0.45	26.18	58.26	0.98	0.95	1.00
cg16290431	5	CPEB4	3.761E-26	0.48	38.03	78.79	0.98	0.95	1.00
cg16657479	2	LOC150786	3.61E-24	0.28	20.67	73.34	0.98	0.95	1.00
cg17297362	2	GIGYF2	3.106E-26	0.45	32.70	73.00	0.98	0.95	1.00
cg17494438	10	DRGX	1.624E-29	0.45	19.88	44.19	0.98	0.95	1.00
cg17584576	5	NDUFS4	1.497E-14	2.01	24.36	12.09	0.98	0.95	1.00
cg18349405	3	VEPH1;PTX3	6.208E-26	0.47	36.62	78.60	0.98	0.95	1.00
cg18582010	8	ASAP1	1.63E-26	0.48	36.16	74.94	0.98	0.95	1.00
cg18989536	7	NAMPT	1.779E-26	0.47	34.91	73.90	0.98	0.95	1.00

cg19138214	4	SMR3B	1.998E-26	0.46	33.93	73.19	0.98	0.95	1.00
cg19486756	5	ZNF300	2.744E-26	0.46	34.60	74.62	0.98	0.95	1.00
cg20298425	8	INTS8	1.138E-25	0.42	32.03	75.51	0.98	0.95	1.00
cg21230547	12	WNK1	4.628E-27	0.49	34.85	70.75	0.98	0.95	1.00
cg21321768	18	ESCO1	9.053E-28	0.36	17.80	50.14	0.98	0.95	1.00
cg21574349	3	MAP3K13	1.105E-46	3.60	30.63	8.51	0.98	0.95	1.00
cg21956434	19	C19orf62	2.433E-42	2.16	26.03	12.03	0.98	0.95	1.00
cg22226592	1	GPSM2	4.546E-29	0.45	21.79	48.06	0.98	0.95	1.00
cg22289694	15	ETFA	1.717E-26	0.40	26.27	65.18	0.98	0.95	1.00
cg22431992	8	RBPMS	7.965E-51	2.46	48.09	19.53	0.98	0.95	1.00
cg22488158	1	WDR63	4.028E-16	2.09	24.81	11.84	0.98	0.95	1.00
cg22539294	3	ZBBX	1.981E-25	0.40	30.01	74.89	0.98	0.95	1.00
cg22887484	5	GPRIN1	7.637E-46	3.91	27.77	7.09	0.98	0.95	1.00
cg22988651	3	ANKRD28	6.081E-26	0.40	27.46	69.39	0.98	0.95	1.00
cg23261372	19	QTRT1	2.101E-46	2.91	32.99	11.34	0.98	0.95	1.00
cg23574427	4	BBS7;CCNA2	5.815E-27	0.46	30.80	67.21	0.98	0.95	1.00
cg23717593	1	COL24A1	2.863E-25	0.35	24.85	70.67	0.98	0.95	1.00
cg25274185	13	FRY	2.823E-29	0.46	21.79	47.15	0.98	0.95	1.00
cg25322008	11	OR51E2	2.723E-27	0.49	33.90	68.62	0.98	0.95	1.00
cg25496104	12	DERA	1.177E-25	0.39	28.24	71.80	0.98	0.95	1.00
cg25663524	4	NFKB1	1.612E-26	0.48	36.34	75.10	0.98	0.95	1.00
cg25954194	5	IL5	2.182E-28	0.48	27.66	57.05	0.98	0.95	1.00
cg25960769	6	TREML2	3.741E-45	2.14	36.49	17.05	0.98	0.95	1.00
cg26309261	3	DBR1	6.827E-26	0.49	40.88	83.09	0.98	0.95	1.00
cg26680517	2	GEMIN6	7.696E-46	2.20	37.95	17.28	0.98	0.95	1.00
cg27128322	18	KIAA1632	3.121E-05	0.42	3.08	7.29	0.98	0.95	1.00
cg27326661	8	MRPS28	1.641E-26	0.50	38.06	76.86	0.98	0.95	1.00
ch.4.1080479F	4	DCUN1D4	5.755E-44	2.34	30.07	12.84	0.98	0.95	1.00
ch.8.885117R	8	PROSC	2.424E-42	2.22	25.50	11.50	0.98	0.95	1.00
cg00232952	18	FAM59A	1.708E-26	0.36	21.43	60.33	0.98	0.93	1.00
cg02226934	4	MTNR1A	3.811E-27	0.46	29.99	65.45	0.98	0.93	1.00
cg02502358	17	C17orf108	1.527E-43	2.18	30.32	13.91	0.98	0.93	1.00
cg02572463	7	SEMA3E	8.355E-43	2.28	26.65	11.71	0.98	0.93	1.00
cg04088433	12	DCN;DCN	1.519E-26	0.47	33.70	72.32	0.98	0.93	1.00
cg04279164	11	GUCY1A2	6.607E-30	0.46	19.66	42.31	0.98	0.93	1.00
cg04322162	2	GULP1	1.968E-26	0.46	33.05	72.28	0.98	0.93	1.00
cg04600792	7	CHN2	2.648E-28	0.38	18.31	48.09	0.98	0.93	1.00
cg04788575	1	TIPRL	3.11E-26	0.47	36.20	76.51	0.98	0.93	1.00
cg04969808	3	WNT7A	4.484E-09	2.01	16.40	8.15	0.98	0.93	1.00
cg05978010	8	MRPL13	9.113E-26	0.48	38.90	81.83	0.98	0.93	1.00
cg06517794	1	USP33	1.783E-25	0.43	34.03	78.64	0.98	0.93	1.00
cg06695691	4	SPATA5	1.785E-26	0.45	31.51	70.51	0.98	0.93	1.00
cg06758255	16	TOX3	6.832E-42	3.98	17.44	4.38	0.98	0.93	1.00
cg06817539	4	KLHL2	6.728E-26	0.47	37.18	79.35	0.98	0.93	1.00
cg06817669	15	C15orf5;SGK269	2.372E-26	0.49	37.49	77.15	0.98	0.93	1.00
cg06947913	12	FAIM2	0.0428045	0.47	1.77	3.76	0.98	0.93	1.00
cg07202461	18	MIB1;MIR133A1	4.996E-26	0.44	32.35	73.81	0.98	0.93	1.00
cg07628631	12	KIAA1033	1.461E-26	0.47	34.16	72.68	0.98	0.93	1.00

cg08125539	17	IGF2BP1	2.44E-25	0.31	20.38	65.79	0.98	0.93	1.00
cg08185798	12	ALX1	2.255E-26	0.46	33.34	72.88	0.98	0.93	1.00
cg08762306	16	PALB2;DCTN5	6.105E-18	0.42	8.70	20.77	0.98	0.93	1.00
cg08900101	12	TBX5	8.91E-45	3.29	26.94	8.18	0.98	0.93	1.00
cg09231673	11	DEPDC7	1.555E-26	0.49	37.07	75.75	0.98	0.93	1.00
cg09460983	6	BTN3A3	4.285E-42	2.32	23.73	10.24	0.98	0.93	1.00
cg09517251	1	FRRS1	5.694E-46	2.78	32.66	11.76	0.98	0.93	1.00
cg10194791	16	KIFC3	0.0019036	0.45	2.52	5.59	0.98	0.93	1.00
cg10362113	14	PAPOLA	7.155E-27	0.47	33.32	70.21	0.98	0.93	1.00
cg11216823	8	WWP1	1.066E-26	0.35	20.53	58.33	0.98	0.93	1.00
cg11398081	7	NPVF	1.381E-26	0.44	29.64	68.03	0.98	0.93	1.00
cg11653179	1	RAVER2	2.023E-26	0.50	38.63	77.92	0.98	0.93	1.00
cg12772418	6	HDAC2	5.311E-16	2.38	20.62	8.67	0.98	0.93	1.00
cg13634966	8	ESCO2	1.167E-26	0.46	32.69	70.70	0.98	0.93	1.00
cg13912027	11	FCHSD2	9.628E-55	3.06	50.09	16.35	0.98	0.93	1.00
cg14137286	10	ZNF438	7.441E-27	0.48	34.51	71.49	0.98	0.93	1.00
cg14639562	9	CENPP;ASPN	6.698E-26	0.48	38.15	80.32	0.98	0.93	1.00
cg15279766	6	BTN3A1	1.06E-44	3.25	26.88	8.27	0.98	0.93	1.00
cg15350036	7	CROT;TP53TG1	2.902E-27	0.48	31.71	66.57	0.98	0.93	1.00
cg15959270	13	DNAJC3	2.987E-27	0.44	27.14	62.07	0.98	0.93	1.00
cg17235179	5	CNOT8	1.575E-44	2.07	35.46	17.16	0.98	0.93	1.00
cg17554896	3	SMC4;MIR16-2;<br>MIR15B	3.862E-26	0.42	29.65	70.48	0.98	0.93	1.00
cg17788761	1	FAM73A	3.272E-26	0.43	30.93	71.36	0.98	0.93	1.00
cg17820060	1	COL11A1	6.536E-27	0.50	36.63	73.31	0.98	0.93	1.00
cg18094221	3	RHOA;TCTA	7.19E-43	2.38	25.97	10.90	0.98	0.93	1.00
cg18974903	8	TNKS	1.287E-26	0.49	36.64	74.87	0.98	0.93	1.00
cg19115941	12	IQSEC3	4.969E-10	0.44	5.79	13.18	0.98	0.93	1.00
cg19200496	11	C11orf65	1.533E-11	2.11	18.59	8.79	0.98	0.93	1.00
cg19298498	11	YAP1	3.795E-25	0.41	32.60	79.15	0.98	0.93	1.00
cg20000937	8	FGL1	6.261E-47	2.80	35.08	12.54	0.98	0.93	1.00
cg20735720	4	FGG	5.225E-27	0.41	25.31	61.48	0.98	0.93	1.00
cg20838429	2	FAP	1.072E-28	0.37	16.54	44.50	0.98	0.93	1.00
cg21295467	11	VWA5A	1.51E-43	2.28	29.30	12.88	0.98	0.93	1.00
cg21884374	7	NRCAM	3.54E-26	0.38	24.90	65.52	0.98	0.93	1.00
cg22367981	1	DISC1;TSNAX	5.117E-26	0.46	35.87	77.38	0.98	0.93	1.00
cg22501608	15	IDH3A	1.135E-26	0.48	35.71	73.66	0.98	0.93	1.00
cg23151860	13	RNF6	1.036E-26	0.50	37.66	75.39	0.98	0.93	1.00
cg23259713	10	CSGALNACT2	4.504E-26	0.45	33.31	74.51	0.98	0.93	1.00
cg23722778	6	ENPP4	1.019E-26	0.48	35.08	72.77	0.98	0.93	1.00
cg23894205	12	C12orf69;C12orf60	3.903E-26	0.41	27.88	68.73	0.98	0.93	1.00
cg24337235	3	EPHA6	1.356E-26	0.50	38.29	76.64	0.98	0.93	1.00
cg24524451	7	ADCK2	2.065E-11	0.42	5.82	13.84	0.98	0.93	1.00
cg25220460	8	ZHX2	1.699E-06	0.39	3.14	7.99	0.98	0.93	1.00
cg25247351	6	HLA-DMB	9.651E-14	2.09	21.92	10.50	0.98	0.93	1.00
cg26422329	14	SAV1	7.558E-27	0.49	35.07	72.08	0.98	0.93	1.00
cg26505663	15	GRINL1A	3.377E-13	0.40	5.78	14.53	0.98	0.93	1.00
cg26810925	12	NAP1L1	3.432E-26	0.45	32.60	73.15	0.98	0.93	1.00

ch.11.2619180F	11	ACRV1	1.426E-43	3.25	23.80	7.33	0.98	0.93	1.00
ch.13.1085822R	13	KLF12	1.219E-49	2.65	43.05	16.21	0.98	0.93	1.00
cg00459447	7	THAP5;DNAJB9	1.387E-08	0.45	5.41	11.99	0.97	0.92	1.00
cg01083380	3	MECOM	1.061E-44	2.84	28.71	10.10	0.97	0.92	1.00
cg02100604	1	CEP350	3.222E-26	0.46	35.07	75.47	0.97	0.92	1.00
cg02716635	12	RMST	1.791E-45	2.08	38.60	18.58	0.97	0.92	1.00
cg02836965	8	SGCZ	7.333E-27	0.48	33.64	70.58	0.97	0.92	1.00
cg03320492	1	C1orf228	1.16E-48	2.07	48.99	23.65	0.97	0.92	1.00
cg04316673	1	BTF3L4	1.248E-26	0.44	29.95	68.11	0.97	0.92	1.00
cg04322202	4	EPHA5	1.716E-53	2.61	52.16	19.99	0.97	0.92	1.00
cg04383352	3	ZNF654	4.066E-27	0.48	32.31	67.92	0.97	0.92	1.00
cg04387396	10	SGPL1	1.409E-17	2.32	23.21	10.02	0.97	0.92	1.00
cg04835652	12	CCDC41	4.259E-26	0.48	37.74	78.80	0.97	0.92	1.00
cg05229965	10	ADAM12	8.766E-47	2.57	36.52	14.23	0.97	0.92	1.00
cg05270007	6	C6orf204;PLN	3.681E-26	0.45	33.16	73.88	0.97	0.92	1.00
cg05592581	3	SIDT1	9.656E-27	0.45	31.05	68.62	0.97	0.92	1.00
cg06888547	2	CPS1	2.771E-26	0.46	34.23	74.26	0.97	0.92	1.00
cg06931815	7	GPR85	2.12E-13	2.61	16.00	6.14	0.97	0.92	1.00
cg09252677	11	ZBED5	2.669E-26	0.45	32.76	72.71	0.97	0.92	1.00
cg09517751	1	IRF2BP2	1.529E-45	3.36	28.69	8.55	0.97	0.92	1.00
cg10312081	13	PSPC1	2.892E-29	0.45	20.49	45.89	0.97	0.92	1.00
cg10632328	17	FLJ35220; LOC100294362	2.278E-14	0.47	9.99	21.05	0.97	0.92	1.00
cg11520439	9	LOC286367	3.235E-27	0.46	29.51	64.61	0.97	0.92	1.00
cg11532054	1	TNFSF18	1.035E-27	0.45	27.06	59.68	0.97	0.92	1.00
cg12044531	14	NIN	1.027E-26	0.46	31.74	69.45	0.97	0.92	1.00
cg12099990	12	MED13L	9.711E-26	0.42	31.01	74.10	0.97	0.92	1.00
cg12965421	7	ACN9	6.095E-27	0.50	36.15	72.68	0.97	0.92	1.00
cg13024275	3	NFKBIZ	2.944E-26	0.40	26.61	66.79	0.97	0.92	1.00
cg13530711	8	KHDRBS3	1.521E-48	2.38	43.33	18.17	0.97	0.92	1.00
cg13883904	4	FIP1L1	2.876E-27	0.47	30.78	65.63	0.97	0.92	1.00
cg13885155	2	WIPF1	1.986E-44	2.17	33.52	15.41	0.97	0.92	1.00
cg14526459	6	C6orf145	3.751E-09	2.49	12.23	4.92	0.97	0.92	1.00
cg15331578	7	BMPER	2.96E-26	0.41	27.56	67.75	0.97	0.92	1.00
cg16371229	1	PCNXL2	3.546E-46	2.55	34.97	13.71	0.97	0.92	1.00
cg17126573	1	C1orf101	3.413E-26	0.49	38.80	79.33	0.97	0.92	1.00
cg17153138	6	ARHGAP18	2.151E-43	2.78	25.16	9.04	0.97	0.92	1.00
cg17282428	6	C6orf103	4.151E-26	0.46	35.05	76.05	0.97	0.92	1.00
cg17326855	1	GDAP2	3.969E-27	0.50	35.16	70.72	0.97	0.92	1.00
cg17449026	1	C1orf173	2.736E-26	0.48	36.88	76.89	0.97	0.92	1.00
cg17771031	20	CDH22	4.239E-42	2.58	22.02	8.52	0.97	0.92	1.00
cg18559571	12	LIMA1	5.395E-27	0.49	34.40	70.65	0.97	0.92	1.00
cg19873842	12	RASSF8	2.497E-27	0.49	32.90	67.43	0.97	0.92	1.00
cg20083989	14	DAD1	1.433E-15	2.13	23.53	11.05	0.97	0.92	1.00
cg20320936	1	STMN1	2.074E-44	2.22	32.87	14.80	0.97	0.92	1.00
cg20597486	1	IFI16	6.436E-45	2.24	34.32	15.30	0.97	0.92	1.00
cg21051568	5	SEMA5A	2.044E-26	0.45	32.74	72.06	0.97	0.92	1.00
cg22189125	19	ZNF302	3.904E-27	0.50	34.91	70.43	0.97	0.92	1.00

cg22936253	10	FAS	3.131E-27	0.47	30.93	65.96	0.97	0.92	1.00
cg23138119	2	GCA	9.231E-26	0.45	35.76	78.72	0.97	0.92	1.00
cg23146699	3	MAGI1	7.852E-27	0.45	29.76	66.86	0.97	0.92	1.00
cg23258678	1	NR5A2	1.729E-43	2.56	26.76	10.45	0.97	0.92	1.00
cg25113081	3	TRIM59	3.844E-27	0.49	33.88	69.36	0.97	0.92	1.00
cg25609301	14	KCNH5	6.092E-27	0.49	34.76	71.28	0.97	0.92	1.00
cg25629773	6	RFX6	1.575E-45	2.39	34.58	14.46	0.97	0.92	1.00
cg25718604	20	TUBB1	1.868E-27	0.46	28.31	62.21	0.97	0.92	1.00
cg25726699	6	C6orf217	5.274E-27	0.49	34.23	70.42	0.97	0.92	1.00
cg26323655	8	RAD54B	3.499E-27	0.41	24.74	60.02	0.97	0.92	1.00
cg26427896	2	ALK	1.843E-26	0.44	30.91	69.98	0.97	0.92	1.00
cg26853855	2	CSRNP3	7.896E-27	0.48	34.91	72.02	0.97	0.92	1.00
cg27405946	12	CHPT1	1.806E-26	0.48	36.21	75.24	0.97	0.92	1.00
cg27539720	17	MED13	1.312E-26	0.42	27.20	65.48	0.97	0.92	1.00
ch.11.1421098F	11	SAPS3	2.212E-48	3.24	36.02	11.12	0.97	0.92	1.00
ch.2.3935902R	2	SF3B1	8.479E-43	2.16	27.79	12.86	0.97	0.92	1.00
ch.7.592198R	7	IGF2BP3	1.676E-44	2.22	33.20	14.95	0.97	0.92	1.00
cg00320354	4	TSPAN5	1.011E-26	0.49	35.64	73.32	0.97	0.91	1.00
cg00640053	7	CNPY1	5.848E-27	0.48	33.32	69.75	0.97	0.91	1.00
cg01739304	6	NHSL1	2.659E-26	0.42	28.46	68.40	0.97	0.91	1.00
cg01754290	7	FOXP2	1.208E-25	0.43	33.18	76.81	0.97	0.91	1.00
cg04780629	17	RNF43	1.922E-14	2.22	20.82	9.38	0.97	0.91	1.00
cg05050884	3	ADIPOQ	2.447E-45	2.82	30.62	10.84	0.97	0.91	1.00
cg05491695	11	MALAT1	7.576E-43	2.86	23.11	8.08	0.97	0.91	1.00
cg05582979	4	CLNK	1.387E-46	2.75	34.50	12.55	0.97	0.91	1.00
cg06963192	1	C1orf9	2.754E-27	0.48	32.64	67.39	0.97	0.91	1.00
cg08233331	18	C18orf2	9.808E-26	0.43	32.04	75.15	0.97	0.91	1.00
cg08239325	14	GMFB	2.905E-26	0.48	36.60	76.75	0.97	0.91	1.00
cg08687449	15	SV2B	4.696E-46	2.92	32.00	10.96	0.97	0.91	1.00
cg08746986	7	RELN	1.189E-27	0.48	30.70	63.62	0.97	0.91	1.00
cg09371456	7	FAM131B	3.422E-05	0.39	2.64	6.70	0.97	0.91	1.00
cg09827440	7	LOC401397	7.178E-08	3.42	8.22	2.40	0.97	0.91	1.00
cg09863786	1	PSMB2	1.443E-48	3.41	35.65	10.46	0.97	0.91	1.00
cg10796679	12	ERP29;TMEM116	0.0006025	0.49	3.47	7.05	0.97	0.91	1.00
cg11701583	12	NDUFA4L2	2.511E-17	2.82	18.40	6.52	0.97	0.91	1.00
cg11885965	12	FAM19A2	2.397E-25	0.36	25.95	71.32	0.97	0.91	1.00
cg12286861	10	SHOC2;RPL13AP6	4.083E-26	0.46	35.31	76.27	0.97	0.91	1.00
cg12616941	10	BMI1	6.867E-27	0.48	33.36	70.16	0.97	0.91	1.00
cg13437084	2	HADHB	9.277E-07	0.37	2.80	7.67	0.97	0.91	1.00
cg13848990	11	OR5W2	1.762E-27	0.50	33.48	67.25	0.97	0.91	1.00
cg13985868	9	WDR31	0.0009628	0.48	3.15	6.53	0.97	0.91	1.00
cg14437446	9	KIAA0020	9.885E-27	0.48	34.16	71.78	0.97	0.91	1.00
cg15001633	8	UBR5	2.49E-28	0.48	27.65	57.30	0.97	0.91	1.00
cg15192986	13	CPB2	1.211E-26	0.50	37.65	75.74	0.97	0.91	1.00
cg15929181	6	FILIP1	4.901E-26	0.45	33.93	75.34	0.97	0.91	1.00
cg17167832	1	SELP	1.251E-26	0.48	35.71	73.88	0.97	0.91	1.00
cg17480803	6	HCG27	2.796E-47	2.06	44.99	21.86	0.97	0.91	1.00
cg17948986	13	KCNRG;DLEU2	1.103E-26	0.47	33.42	71.30	0.97	0.91	1.00

cg17974460	2	MPP4	2.958E-27	0.47	30.40	65.31	0.97	0.91	1.00
cg18611813	5	PDE4D;PART1	4.818E-26	0.42	29.50	70.87	0.97	0.91	1.00
cg19069882	11	MPZL3	8.14E-16	2.13	23.84	11.20	0.97	0.91	1.00
cg19611602	3	CNTN3	2.009E-26	0.42	27.93	67.21	0.97	0.91	1.00
cg21579472	1	PIK3R3	4.335E-27	0.50	35.33	71.08	0.97	0.91	1.00
cg22082665	1	NCRNA00201	1.093E-26	0.49	35.98	73.84	0.97	0.91	1.00
cg22240515	13	PRHOXNB	1.241E-45	2.12	38.43	18.13	0.97	0.91	1.00
cg22468751	6	MAP7	2.28E-50	2.19	51.36	23.45	0.97	0.91	1.00
cg22613769	1	SMYD2	1.083E-27	0.41	22.57	55.29	0.97	0.91	1.00
cg23680411	12	SLC2A13	2.43E-26	0.48	36.21	75.94	0.97	0.91	1.00
cg25191332	7	IFRD1	1.356E-45	2.60	32.89	12.65	0.97	0.91	1.00
cg26477387	6	ZNF292	4.655E-26	0.43	31.18	72.46	0.97	0.91	1.00
cg26609386	8	MRPL13;MTBP	3.138E-42	2.62	22.26	8.49	0.97	0.91	1.00
ch.11.1652751R	11	C11orf30	1.245E-47	2.90	36.15	12.46	0.97	0.91	1.00
ch.3.1113149F	3	VPRBP	1.074E-42	2.24	26.65	11.92	0.97	0.91	1.00
cg00350932	2	PTCD3	2.463E-27	0.49	20.97	42.40	0.96	0.90	1.00
cg00957886	8	PDE7A	1.458E-27	0.48	31.08	64.44	0.96	0.90	1.00
cg01022974	4	TRIM2	0.0001312	2.07	8.60	4.16	0.96	0.90	1.00
cg04770127	13	EPSTI1	4.771E-26	0.40	27.16	68.50	0.96	0.90	1.00
cg05109049	17	NF1;EVI2B	2.066E-48	3.21	36.24	11.30	0.96	0.90	1.00
cg06721860	12	SYT10	2.133E-27	0.50	33.51	67.70	0.96	0.90	1.00
cg07442476	3	TBL1XR1	6.505E-28	0.44	25.29	56.93	0.96	0.90	1.00
cg08329070	12	FGFR1OP2	9.432E-28	0.48	29.60	62.03	0.96	0.90	1.00
cg08404328	4	SRD5A3	8.207E-43	2.08	28.86	13.89	0.96	0.90	1.00
cg09070142	7	PTPRZ1	2.256E-27	0.48	31.40	65.71	0.96	0.90	1.00
cg09092344	5	SRFBP1	6.023E-28	0.49	29.96	61.44	0.96	0.90	1.00
cg09754269	2	RASGRP3	2.612E-15	2.38	19.89	8.36	0.96	0.90	1.00
cg10153353	11	METT5D1	4.722E-26	0.45	33.44	74.75	0.96	0.90	1.00
cg10289305	3	PEX5L	3.111E-26	0.49	39.38	79.69	0.96	0.90	1.00
cg10440342	7	NUPL2	2.986E-26	0.49	38.81	79.02	0.96	0.90	1.00
cg10838627	1	MGC27382	2.146E-26	0.45	31.69	71.12	0.96	0.90	1.00
cg11083276	6	FAM65B	4.032E-08	0.44	4.82	10.97	0.96	0.90	1.00
cg11144775	1	SFRS11	1.027E-25	0.47	37.58	80.80	0.96	0.90	1.00
cg11276388	6	HIVEP1	1.792E-27	0.48	19.46	40.40	0.96	0.90	1.00
cg11668188	1	PER3	5.008E-26	0.47	37.30	78.75	0.96	0.90	1.00
cg12201332	14	RALGAPA1	3.176E-26	0.49	39.39	79.75	0.96	0.90	1.00
cg12786620	7	SYPL1	3.693E-26	0.41	28.20	68.92	0.96	0.90	1.00
cg13437307	8	CLVS1	7.453E-27	0.48	34.52	71.50	0.96	0.90	1.00
cg13827209	9	TGFBR1	1.419E-43	2.14	30.92	14.44	0.96	0.90	1.00
cg14305943	10	SNORA19;EIF3A	1.109E-27	0.45	27.28	60.05	0.96	0.90	1.00
cg14456143	2	GEN1	6.011E-26	0.46	35.54	77.44	0.96	0.90	1.00
cg15937191	10	LIPF	2.506E-26	0.48	36.77	76.56	0.96	0.90	1.00
cg16119667	2	ZNF514	6.592E-08	0.41	4.10	9.92	0.96	0.90	1.00
cg16801374	12	IGF1	5.126E-10	2.17	15.83	7.30	0.96	0.90	1.00
cg18026227	8	LOC100192378; ZFHX4	8.432E-44	2.59	27.55	10.64	0.96	0.90	1.00
cg19084281	5	PCSK1	7.264E-25	0.39	31.50	79.77	0.96	0.90	1.00
cg20702295	22	DGCR6L	0.0001887	0.48	3.68	7.61	0.96	0.90	1.00

cg22160911	5	ZFYVE16	9.475E-26	0.44	34.28	77.30	0.96	0.90	1.00
cg22901919	4	CLGN	1.387E-26	0.50	37.88	76.29	0.96	0.90	1.00
cg23127434	2	NCKAP1	3.995E-26	0.37	23.73	64.64	0.96	0.90	1.00
cg23629032	21	RNF160	4.154E-26	0.40	27.08	68.08	0.96	0.90	1.00
cg24336674	2	CD28	2.692E-27	0.46	30.09	64.79	0.96	0.90	1.00
cg25506747	3	SHOX2	7.582E-49	2.90	39.12	13.49	0.96	0.90	1.00
cg27478224	10	IFIT2	4.836E-46	2.50	35.01	13.99	0.96	0.90	1.00
ch.7.2714749F	7	FLJ43663	9.09E-43	2.07	28.82	13.95	0.96	0.90	1.00
cg00426193	2	RIF1	2.152E-42	2.19	25.98	11.87	0.96	0.89	1.00
cg02146383	16	C16orf52	9.831E-27	0.48	35.39	73.00	0.96	0.89	1.00
cg02820309	1	OPN3	7.098E-48	2.63	38.84	14.75	0.96	0.89	1.00
cg02927202	2	SP3;SP3	1.51E-28	0.47	25.08	53.72	0.96	0.89	1.00
cg03535830	1	SLC16A4	5.744E-28	0.48	29.37	60.75	0.96	0.89	1.00
cg03541057	1	ANP32E	2.535E-48	2.08	47.85	23.05	0.96	0.89	1.00
cg04380955	17	ABCA6	1.009E-26	0.49	36.10	73.77	0.96	0.89	1.00
cg04733681	6	IGF2R	1.705E-26	0.42	27.76	66.64	0.96	0.89	1.00
cg06583605	4	CEP135	3.833E-26	0.35	22.05	62.86	0.96	0.89	1.00
cg06686746	6	TMEM170B	2.599E-12	2.09	20.02	9.59	0.96	0.89	1.00
cg07884735	4	SDAD1	7.902E-44	2.57	27.74	10.78	0.96	0.89	1.00
cg09066112	12	SOX5;SOX5	7.284E-26	0.39	27.01	69.38	0.96	0.89	1.00
cg09744448	5	ADAM19	2.87E-45	2.50	32.76	13.11	0.96	0.89	1.00
cg09754341	11	ARHGEF12	4.032E-26	0.47	35.67	76.61	0.96	0.89	1.00
cg10157954	11	ZNF215	3.806E-26	0.47	35.95	76.74	0.96	0.89	1.00
cg11271345	5	ACTBL2	4.389E-27	0.49	34.44	70.22	0.96	0.89	1.00
cg12051027	1	SPATA6	1.385E-27	0.49	32.32	65.57	0.96	0.89	1.00
cg12163448	2	FASTKD1	8.611E-12	2.36	16.16	6.85	0.96	0.89	1.00
cg13362637	5	CD74	1.992E-48	2.23	45.20	20.23	0.96	0.89	1.00
cg14194344	8	PPP2R2A	1.204E-25	0.48	39.81	83.43	0.96	0.89	1.00
cg15336243	10	FAM171A1	1.831E-49	2.91	40.48	13.92	0.96	0.89	1.00
cg18237739	10	TMEM26	5.377E-26	0.46	35.55	77.18	0.96	0.89	1.00
cg18512262	11	OR56A1	1.373E-26	0.49	36.69	75.07	0.96	0.89	1.00
cg22143698	5	ANKRD33B	2.453E-17	2.10	26.19	12.49	0.96	0.89	1.00
cg23542449	12	KIAA0528	1.533E-26	0.49	36.96	75.59	0.96	0.89	1.00
cg23807894	20	TPX2	1.582E-42	2.08	27.73	13.35	0.96	0.89	1.00
cg25821783	17	CCDC46	3.439E-43	2.61	25.46	9.74	0.96	0.89	1.00
cg27080171	4	GABRA2	6.049E-28	0.44	15.56	35.09	0.96	0.89	1.00
cg00347643	7	YWHAG	1.619E-44	2.83	28.26	9.99	0.95	0.88	1.00
cg00474242	3	WDR5B	3.895E-43	3.06	23.20	7.59	0.95	0.88	1.00
cg01418536	17	USP32	2.212E-27	0.46	29.24	63.51	0.95	0.88	1.00
cg03307717	21	U2AF1	1.973E-48	2.91	38.04	13.06	0.95	0.88	1.00
cg04540039	22	SYN3	7.013E-44	2.36	29.65	12.58	0.95	0.88	1.00
cg04651658	5	AFF4	9.43E-18	2.10	26.83	12.75	0.95	0.88	1.00
cg05388821	10	FAM160B1	6.314E-44	2.70	27.23	10.07	0.95	0.88	1.00
cg08313420	7	DAGLB	2.204E-16	2.04	26.07	12.75	0.95	0.88	1.00
cg08414021	3	VPS8	9.97E-27	0.50	37.48	75.12	0.95	0.88	1.00
cg09682986	7	POT1	4.448E-26	0.44	31.84	73.01	0.95	0.88	1.00
cg11680857	1	LCE2D	4.68E-28	0.50	30.44	61.39	0.95	0.88	1.00
cg12068908	11	SPON1	9.467E-45	2.34	32.64	13.94	0.95	0.88	1.00

cg13325919	3	SOX2;SOX2OT	8.597E-28	0.47	28.71	60.94	0.95	0.88	1.00
cg13360230	1	ZNF238	1.107E-42	2.14	27.55	12.85	0.95	0.88	1.00
cg13441107	9	EXOSC3	7.607E-28	0.50	31.58	63.55	0.95	0.88	1.00
cg14649914	8	CSMD3	5.827E-48	2.39	41.69	17.46	0.95	0.88	1.00
cg15323936	3	ACAP2	5.73E-27	0.50	35.78	72.16	0.95	0.88	1.00
cg15344854	4	SMARCAD1	3.177E-43	2.62	25.53	9.74	0.95	0.88	1.00
cg16068495	15	MEF2A	2.721E-25	0.45	37.33	83.02	0.95	0.88	1.00
cg16556111	5	NR2F1	3.311E-43	2.95	23.84	8.09	0.95	0.88	1.00
cg19368016	10	CUGBP2	1.672E-42	2.80	22.27	7.94	0.95	0.88	1.00
cg20992361	7	DGKB	8.305E-28	0.49	30.71	62.87	0.95	0.88	1.00
cg21158075	12	PRR4;PRH1	4.472E-27	0.46	30.87	66.69	0.95	0.88	1.00
cg21216600	7	DGKI	2.278E-45	2.33	34.77	14.94	0.95	0.88	1.00
cg21447227	7	WDR60	3.61E-11	2.43	14.91	6.14	0.95	0.88	1.00
cg22304522	8	ADCY8	2.483E-27	0.47	30.83	65.35	0.95	0.88	1.00
cg22380472	19	ZNF57	3.041E-06	0.46	4.40	9.50	0.95	0.88	1.00
cg22879408	11	ELMOD1	6.753E-44	2.41	29.24	12.14	0.95	0.88	1.00
cg25212814	6	C6orf164	1.1E-26	0.46	31.90	69.76	0.95	0.88	1.00
cg25769852	12	CD69	1.734E-47	2.81	36.40	12.94	0.95	0.88	1.00
ch.18.1114073F	18	ZNF532	2.208E-44	2.58	29.44	11.42	0.95	0.88	1.00
ch.18.925473R	18	ACAA2	3.077E-46	2.71	33.85	12.49	0.95	0.88	1.00
ch.5.2450351R	5	SKP1	3.873E-46	3.55	29.51	8.32	0.95	0.88	1.00
ch.6.839713R	6	RPS10	3.75E-42	2.56	22.31	8.70	0.95	0.88	1.00
cg00517849	4	ODZ3	3.089E-27	0.49	34.30	69.31	0.95	0.87	1.00
cg03262242	1	DNAJC8	1.096E-08	0.50	7.12	14.34	0.95	0.87	1.00
cg04773529	3	LPP	3.14E-44	3.22	25.71	7.97	0.95	0.87	1.00
cg05300946	14	C14orf1	1.253E-41	3.69	17.15	4.65	0.95	0.87	1.00
cg05671385	11	CASP1;CARD16	3.47E-45	2.27	34.83	15.32	0.95	0.87	1.00
cg06654604	7	SAMD9L	1.748E-44	2.44	30.84	12.63	0.95	0.87	1.00
cg08186384	6	RPS6KA2	3.373E-11	2.37	15.43	6.52	0.95	0.87	1.00
cg08310519	11	ME3	6.96E-25	0.38	30.13	78.28	0.95	0.87	1.00
cg08887114	1	C1orf26	4.58E-27	0.50	35.26	71.14	0.95	0.87	1.00
cg10110884	3	AADAC	1.768E-26	0.49	38.07	77.04	0.95	0.87	1.00
cg11643991	11	CTNND1	2.842E-45	2.66	31.52	11.86	0.95	0.87	1.00
cg11867012	19	RPS19	6.921E-14	0.46	8.72	19.04	0.95	0.87	1.00
cg13165758	11	GAB2	1.456E-17	2.34	22.89	9.78	0.95	0.87	1.00
cg16328548	2	NCKAP5	1.159E-27	0.50	21.40	43.12	0.95	0.87	1.00
cg16383389	1	MIR760	1.246E-05	0.46	3.99	8.66	0.95	0.87	1.00
cg16794061	6	HLA-DRA	1.134E-42	2.29	26.04	11.36	0.95	0.87	1.00
cg17874670	5	FGF1	1.185E-26	0.47	33.37	71.41	0.95	0.87	1.00
cg18084215	5	PHAX	0.0126942	0.48	2.29	4.76	0.95	0.87	1.00
cg18190030	1	SLC35F3	4.688E-16	2.28	21.82	9.56	0.95	0.87	1.00
cg18537222	3	PPARG	1.148E-29	0.48	22.00	45.67	0.95	0.87	1.00
cg20287074	17	TTLL6	1.469E-10	2.10	17.42	8.30	0.95	0.87	1.00
cg21391790	15	C15orf41	9.482E-27	0.50	37.40	74.93	0.95	0.87	1.00
cg23991274	4	DCK	4.286E-45	2.60	31.43	12.10	0.95	0.87	1.00
cg25332377	2	CCDC108	5.029E-05	2.74	6.75	2.46	0.95	0.87	1.00
cg26305683	4	FAM190A	1.634E-27	0.45	27.24	60.85	0.95	0.87	1.00
cg26673722	4	TMPRSS11A	2.422E-26	0.45	32.96	72.68	0.95	0.87	1.00

cg26726490	15	RASGRP1	1.266E-26	0.47	34.12	72.31	0.95	0.87	1.00
cg26786623	1	PRPF3	7.335E-27	0.48	33.67	70.62	0.95	0.87	1.00
cg27203437	16	LOC100329108; GCSH	0.0140721	0.49	2.38	4.84	0.95	0.87	1.00
ch.1.1248405R	1	MACF1	1.135E-44	3.23	26.89	8.33	0.95	0.87	1.00
ch.18.308031F	18	SEH1L	2.061E-41	3.05	17.91	5.87	0.95	0.87	1.00
cg00804525	16	TMCO7	2.461E-26	0.44	31.20	70.96	0.94	0.86	1.00
cg01664727	21	RUNX1	1.098E-43	2.20	30.66	13.97	0.94	0.86	1.00
cg04152196	6	PDE7B	2.593E-43	3.72	21.82	5.86	0.94	0.86	1.00
cg04784410	14	SNORD113-4	7.082E-28	0.48	29.60	61.42	0.94	0.86	1.00
cg05285759	4	TBCK	3.8E-27	0.50	35.08	70.54	0.94	0.86	1.00
cg05977219	3	LOC344595; LOC100302640	1.125E-07	2.08	13.44	6.48	0.94	0.86	1.00
cg08209474	16	RFWD3	0.0376634	2.09	4.16	1.99	0.94	0.86	1.00
cg10443987	7	ZNF680	2.621E-06	2.22	10.25	4.61	0.94	0.86	1.00
cg10571168	7	LMBR1	2.292E-27	0.50	33.73	68.07	0.94	0.86	1.00
cg12944573	6	OPRM1	8.831E-25	0.47	16.80	35.49	0.94	0.86	1.00
cg13612055	5	RNU5E;RNU5D; ACOT12	5.069E-27	0.48	33.32	69.42	0.94	0.86	1.00
cg17445840	3	FAM55C;NFKBIZ	1.049E-17	2.14	26.00	12.15	0.94	0.86	1.00
cg18456803	13	ELF1	9.446E-47	2.63	35.87	13.63	0.94	0.86	1.00
cg18979559	17	CCDC144C	2.787E-12	0.44	7.09	16.01	0.94	0.86	1.00
cg20034856	3	SLMAP	1.59E-26	0.43	29.42	68.14	0.94	0.86	1.00
cg21188154	5	ODZ2	6.746E-17	2.16	24.50	11.32	0.94	0.86	1.00
cg23576842	18	SETBP1	3.451E-15	2.03	24.95	12.31	0.94	0.86	1.00
cg24860886	1	GRHL3	3.265E-13	2.48	16.69	6.72	0.94	0.86	1.00
cg27452109	11	SIAE	4.419E-43	2.90	23.67	8.17	0.94	0.86	1.00
ch.1.590396F	1	SDHB	1.049E-42	2.01	29.42	14.67	0.94	0.86	1.00
ch.4.3154602F	4	GALNTL6	6.172E-44	2.09	32.92	15.75	0.94	0.86	1.00
ch.7.2086776R	7	BUD31	7.414E-43	2.42	25.67	10.62	0.94	0.86	1.00
cg01005582	3	CTNNB1	6.431E-27	0.43	28.05	64.70	0.94	0.86	1.00
cg01029838	7	PHTF2;TMEM60	2.39E-07	0.46	4.89	10.70	0.94	0.86	1.00
cg01643123	12	PPM1H	4.051E-46	3.78	28.77	7.61	0.94	0.86	1.00
cg02801583	22	RIBC2;SMC1B	1.136E-05	0.47	4.23	8.98	0.94	0.86	1.00
cg04430637	4	COX18	1.532E-17	2.37	22.48	9.49	0.94	0.86	1.00
cg04442806	3	MOBP	2.653E-26	0.47	35.56	75.49	0.94	0.86	1.00
cg04470984	11	FLJ32810	1.599E-26	0.43	29.28	68.02	0.94	0.86	1.00
cg06052255	7	PTN	2.765E-27	0.49	33.54	68.29	0.94	0.86	1.00
cg06382236	7	C7orf58	5.17E-44	4.46	22.32	5.00	0.94	0.86	1.00
cg07194321	9	FAM108B1	6.878E-44	2.12	32.29	15.21	0.94	0.86	1.00
cg07895227	6	CD164	6.533E-44	2.23	31.04	13.92	0.94	0.86	1.00
cg10887385	1	NTNG1	9.922E-25	0.33	23.88	72.98	0.94	0.86	1.00
cg12553223	20	C20orf43	1.554E-45	2.61	32.66	12.53	0.94	0.86	1.00
cg13927247	5	DTWD2	7.293E-27	0.48	34.22	71.15	0.94	0.86	1.00
cg14971439	5	SNX24	3.339E-26	0.45	33.14	73.61	0.94	0.86	1.00
cg15698945	6	TAAR9	2.067E-26	0.47	34.61	73.95	0.94	0.86	1.00
cg16517186	14	MUDENG;EXOC5	5.586E-14	0.46	8.61	18.91	0.94	0.86	1.00
cg16534579	6	PARK2	1.694E-26	0.46	33.09	71.96	0.94	0.86	1.00

cg16698201	6	SNORD50B;SNHG5; SNORD50A	9.349E-44	2.04	32.98	16.15	0.94	0.86	1.00
cg19149314	2	HECW2	9.827E-27	0.47	33.47	71.08	0.94	0.86	1.00
cg19428472	2	MARCH7	1.557E-46	2.85	33.72	11.84	0.94	0.86	1.00
cg19651291	14	LRFN5	1.19E-26	0.41	26.23	64.28	0.94	0.86	1.00
cg20027157	12	PRH1;PRR4	6.738E-43	2.11	28.79	13.66	0.94	0.86	1.00
cg20577535	11	EXPH5	7.432E-45	2.00	37.73	18.84	0.94	0.86	1.00
cg21735068	8	PGCP	3.178E-28	0.44	23.68	53.83	0.94	0.86	1.00
cg22478210	6	HIST1H2BB	5.749E-42	2.41	22.59	9.37	0.94	0.86	1.00
cg23558337	20	C20orf94	6.583E-42	2.50	21.84	8.74	0.94	0.86	1.00
cg23994748	2	FAM168B	7.226E-16	2.60	18.46	7.11	0.94	0.86	1.00
cg24441068	11	EIF3M	2.965E-06	0.38	2.87	7.52	0.94	0.86	1.00
ch.9.1921717R	9	PSMD5	2.481E-43	2.28	28.46	12.46	0.94	0.86	1.00
cg01438737	20	SFRS6	1.923E-07	0.42	4.00	9.57	0.93	0.85	1.00
cg01681236	4	SNCA	1.63E-26	0.49	37.29	76.07	0.93	0.85	1.00
cg02722613	4	SEPSECS	1.429E-44	2.55	30.26	11.89	0.93	0.85	1.00
cg03145312	3	CMTM6	9.903E-15	2.09	23.19	11.11	0.93	0.85	1.00
cg03571129	2	ZC3H6	8.533E-45	2.66	30.11	11.32	0.93	0.85	1.00
cg04395462	1	FNBP1L	1.605E-25	0.45	36.22	80.57	0.93	0.85	1.00
cg06260815	13	CCNA1	6.416E-15	2.09	23.40	11.19	0.93	0.85	1.00
cg06394255	11	NRIP3	5.21E-10	2.14	16.11	7.52	0.93	0.85	1.00
cg07675399	18	CXXC1	0.0011173	0.47	2.93	6.23	0.93	0.85	1.00
cg08572049	12	RSRC2;KNTC1	7.529E-15	2.66	17.02	6.40	0.93	0.85	1.00
cg10122865	2	OTX1	5.581E-05	0.45	3.38	7.53	0.93	0.85	1.00
cg10776104	1	DOCK7	5.268E-26	0.44	32.61	74.19	0.93	0.85	1.00
cg14512008	14	PPP2R5C	2.727E-13	2.36	17.87	7.57	0.93	0.85	1.00
cg18902697	4	FHDC1	1.964E-27	0.48	31.37	65.38	0.93	0.85	1.00
cg22637759	4	SPRY1	2.547E-43	2.13	30.09	14.11	0.93	0.85	1.00
cg23949574	2	HDAC4	6.298E-17	2.85	17.74	6.22	0.93	0.85	1.00
cg27397850	7	EVX1	4.103E-16	2.25	22.38	9.96	0.93	0.85	1.00
ch.15.922567R	15	HERC1	1.703E-41	3.21	17.75	5.53	0.93	0.85	1.00
cg01492656	4	N4BP2;LOC344967	4.351E-42	3.55	18.75	5.28	0.93	0.84	1.00
cg04232649	5	CCNG1	0.0108018	0.50	2.55	5.12	0.93	0.84	1.00
cg05025239	8	RSPO2	3.33E-26	0.45	33.02	73.49	0.93	0.84	1.00
cg07919744	15	KIAA1370	6.334E-27	0.45	30.47	67.08	0.93	0.84	1.00
cg09238199	13	COL4A2	2.473E-16	2.17	23.75	10.95	0.93	0.84	1.00
cg09428349	21	C2CD2	3.158E-42	2.25	24.80	11.03	0.93	0.84	1.00
cg09509863	2	CUL3	1.283E-42	3.13	21.40	6.83	0.93	0.84	1.00
cg09656541	10	C10orf118	2.216E-11	0.48	8.52	17.58	0.93	0.84	1.00
cg11369071	6	LOC100270746; C6orf41	5.119E-07	0.45	4.61	10.17	0.93	0.84	1.00
cg11581263	19	ELOF1	9.235E-11	2.09	17.89	8.58	0.93	0.84	1.00
cg11779273	4	INTU	2.054E-12	2.69	14.52	5.40	0.93	0.84	1.00
cg12259918	8	RB1CC1	8.181E-07	2.21	11.01	4.98	0.93	0.84	1.00
cg12904880	6	TRIM15	5.916E-10	0.47	6.99	14.78	0.93	0.84	1.00
cg13914324	9	PRUNE2	1.963E-42	2.73	22.41	8.22	0.93	0.84	1.00
cg15104818	14	PSMA3	8.523E-45	2.66	30.14	11.35	0.93	0.84	1.00
cg15448144	8	OTUD6B	6.373E-14	2.16	21.06	9.76	0.93	0.84	1.00

cg15675420	19	ZNF136	0.0321635	0.46	1.76	3.84	0.93	0.84	1.00
cg15732657	2	ITGAV	8.206E-27	0.49	35.48	72.68	0.93	0.84	1.00
cg16382778	1	S100A11	6.982E-43	2.72	23.90	8.80	0.93	0.84	1.00
cg16705229	6	NCRNA00171	7.99E-27	0.49	35.55	72.69	0.93	0.84	1.00
cg20322299	2	INSIG2	3.368E-27	0.45	28.70	63.89	0.93	0.84	1.00
cg00777895	1	SRP9	1.265E-25	0.46	16.39	35.29	0.92	0.83	1.00
cg03229183	22	TOP3B	1.182E-13	2.08	21.87	10.49	0.92	0.83	1.00
cg03529432	7	HOXA6	2.04E-12	2.02	21.25	10.51	0.92	0.83	1.00
cg03613077	1	RGS7	2.221E-05	2.03	10.16	5.00	0.92	0.83	1.00
cg06935464	4	TLR10	7.201E-42	2.52	21.55	8.54	0.92	0.83	1.00
cg09751515	11	HBD	6.577E-47	2.09	43.15	20.64	0.92	0.83	1.00
cg14414971	7	SDK1	0.0006669	0.39	2.07	5.31	0.92	0.83	1.00
cg18668813	6	MARCKS	1.768E-15	2.25	21.59	9.60	0.92	0.83	1.00
cg18693004	7	SP4	9.259E-26	0.40	28.52	71.48	0.92	0.83	1.00
cg21123573	12	SIRT4	5.443E-47	2.25	40.70	18.06	0.92	0.83	1.00
cg22664064	2	NMI	6.349E-17	2.12	25.23	11.91	0.92	0.83	1.00
cg26159934	7	SRPK2	0.0012464	0.39	1.96	5.03	0.92	0.83	1.00
cg26690672	4	ABCE1	4.789E-27	0.47	31.31	67.29	0.92	0.83	1.00
cg27081973	5	C5orf44;TRIM23	1.731E-16	2.32	21.88	9.44	0.92	0.83	1.00
cg27086308	7	LOC100132707	7.613E-12	2.58	14.57	5.64	0.92	0.83	1.00
cg27130993	5	ABLIM3	4.307E-27	0.45	15.87	35.19	0.92	0.83	1.00
cg27143246	3	MYNN	4.338E-45	2.39	33.18	13.86	0.92	0.83	1.00
ch.11.942842R	11	PHF21A	8.589E-45	3.43	26.51	7.73	0.92	0.83	1.00
ch.15.979660F	15	PTPLAD1	2.155E-43	2.72	25.47	9.36	0.92	0.83	1.00
ch.4.2714611R	4	ARHGAP10	7.706E-44	3.16	24.87	7.88	0.92	0.83	1.00
cg00857660	1	GAS5;ZBTB37	6.105E-07	2.35	10.33	4.39	0.92	0.82	1.00
cg00949554	2	UBE2E3	0.0052735	0.45	2.28	5.01	0.92	0.82	1.00
cg07297802	11	SLC3A2;SNHG1	1.705E-17	2.36	22.11	9.35	0.92	0.82	1.00
cg09525994	12	PFKM	1.461E-45	2.16	37.55	17.37	0.92	0.82	1.00
cg12697789	4	TLR3	1.452E-42	2.55	23.81	9.35	0.92	0.82	1.00
cg13709449	9	PTCH1	1.4E-08	2.23	13.29	5.96	0.92	0.82	1.00
cg13977600	9	GLIS3	9.588E-27	0.49	36.13	73.68	0.92	0.82	1.00
cg14484356	7	HERPUD2	7.231E-27	0.45	30.64	67.55	0.92	0.82	1.00
cg15067827	1	EDEM3	2.159E-44	2.43	30.64	12.60	0.92	0.82	1.00
cg15716680	11	TEAD1	3.119E-45	2.94	29.66	10.07	0.92	0.82	1.00
cg16725607	3	KLHL6	1.738E-12	2.12	19.72	9.29	0.92	0.82	1.00
cg23288973	5	TRPC7	9.713E-44	2.00	33.54	16.74	0.92	0.82	1.00
cg23571456	6	BTN2A2	1.32E-14	2.01	24.54	12.22	0.92	0.82	1.00
cg26205771	8	NPBWR1	8.094E-44	3.64	23.38	6.43	0.92	0.82	1.00
cg27641628	7	TMEM140	3.514E-11	2.43	14.93	6.16	0.92	0.82	1.00
ch.2.3753920R	2	ZC3H15	1.637E-42	2.38	24.74	10.39	0.92	0.82	1.00
ch.21.366880F	21	URB1	1.582E-42	2.60	23.39	9.01	0.92	0.82	1.00
ch.4.2917740F	4	FNIP2	1.652E-42	2.60	23.33	8.98	0.92	0.82	1.00
cg01959896	6	PDSS2	3.888E-27	0.50	35.05	70.56	0.91	0.81	1.00
cg02837488	7	GTF2IRD1	0.0041619	2.67	4.60	1.72	0.91	0.81	1.00
cg03554194	3	PVRL3	8.622E-27	0.48	34.47	71.78	0.91	0.81	1.00
cg05981038	5	CARD6	3.533E-46	2.06	41.29	20.03	0.91	0.81	1.00
cg06948614	5	PARP8	3.087E-27	0.48	32.77	67.77	0.91	0.81	1.00

cg08720517	5	LOC389333	2.886E-10	2.15	16.41	7.64	0.91	0.81	1.00
cg10372121	13	LOC100188949	4.84E-07	2.30	10.76	4.68	0.91	0.81	1.00
cg11748417	20	LOC388789	1.267E-05	0.38	2.60	6.87	0.91	0.81	1.00
cg13206850	7	ATXN7L1	1.669E-26	0.47	33.87	72.71	0.91	0.81	1.00
cg13531667	5	MCC	7.207E-11	2.09	18.03	8.64	0.91	0.81	1.00
cg17190403	6	C6orf211;RMND1	5.27E-43	2.76	24.06	8.71	0.91	0.81	1.00
cg21301240	8	EIF3H	1.79E-26	0.49	37.69	76.69	0.91	0.81	1.00
cg21363050	4	PDGFRA	7.762E-43	2.17	27.88	12.87	0.91	0.81	1.00
cg24759859	6	SYNCRIP	4.482E-06	0.47	4.44	9.46	0.91	0.81	1.00
cg25032124	1	HBXIP	3.457E-27	0.44	27.59	62.84	0.91	0.81	1.00
cg27388983	19	ZNF256	4.508E-05	0.43	3.11	7.24	0.91	0.81	1.00
ch.12.1038646R	12	DIP2B	5.471E-44	2.57	28.27	11.00	0.91	0.81	1.00
ch.2.4817431F	2	COPS8	1.222E-43	2.59	27.05	10.45	0.91	0.81	1.00
ch.22.757911F	22	XRCC6	9.963E-13	2.13	19.94	9.36	0.91	0.81	1.00
cg01762827	16	HAS3	0.0007158	0.43	2.55	5.88	0.91	0.80	1.00
cg06529477	17	TEX14	2.902E-10	2.81	11.86	4.21	0.91	0.80	1.00
cg07792822	1	KIRREL	2.866E-08	2.04	14.77	7.24	0.91	0.80	1.00
cg08291886	8	ARMC1	5.808E-42	3.41	18.70	5.49	0.91	0.80	1.00
cg08463061	2	RND3	6.698E-09	2.07	15.42	7.46	0.91	0.80	1.00
cg08984614	3	PLCXD2	2.431E-13	2.66	15.61	5.88	0.91	0.80	1.00
cg09115705	1	GPA33	1.483E-10	2.21	16.10	7.28	0.91	0.80	1.00
cg09847027	10	WAC	7.406E-27	0.49	35.97	72.93	0.91	0.80	1.00
cg12601142	12	KDM5A	1.255E-27	0.50	33.02	66.06	0.91	0.80	1.00
cg12609829	15	C15orf53	4.242E-09	2.14	14.90	6.97	0.91	0.80	1.00
cg12766287	10	HNRNPF	1.184E-27	0.50	32.72	65.64	0.91	0.80	1.00
cg16732115	12	PWP1	2.388E-14	2.18	21.30	9.79	0.91	0.80	1.00
cg16955889	3	SOX2OT	3.917E-12	2.14	19.03	8.90	0.91	0.80	1.00
cg19085146	1	KIAA1026	4.813E-41	4.26	14.68	3.45	0.91	0.80	1.00
cg23485307	3	TGFBR2	1.466E-16	2.10	25.19	11.99	0.91	0.80	1.00
cg23946942	5	SCAMP1	1.514E-05	2.01	10.65	5.31	0.91	0.80	1.00
cg25277723	6	MAP3K5	2.504E-53	3.31	45.78	13.82	0.91	0.80	1.00
cg25593954	17	SPOP	2.43E-41	3.02	17.75	5.87	0.91	0.80	1.00
cg26788737	1	C1orf55	5.199E-43	2.29	27.29	11.93	0.91	0.80	1.00
cg01597741	7	MKRN1	2.563E-42	2.63	22.48	8.53	0.90	0.79	1.00
cg02743029	1	SERTAD4;C1orf133	7.746E-46	2.44	35.05	14.39	0.90	0.79	1.00
cg03637218	5	AP3S1	4.038E-29	0.39	16.71	42.76	0.90	0.79	1.00
cg03840259	22	GRAP2	1.109E-45	2.49	34.12	13.73	0.90	0.79	1.00
cg05841967	7	NAA38	2.116E-09	2.10	15.71	7.47	0.90	0.79	1.00
cg06151625	3	RPL15;NKIRAS1	1.294E-05	2.34	8.68	3.71	0.90	0.79	1.00
cg06334134	7	CASP2	3.13E-17	2.24	23.80	10.65	0.90	0.79	1.00
cg11039729	10	CCNY	6.624E-16	2.24	22.16	9.88	0.90	0.79	1.00
cg11224647	8	TMEM55A	0.0038551	2.02	6.27	3.10	0.90	0.79	1.00
cg12621514	10	DKK1	1.259E-43	3.26	23.91	7.33	0.90	0.79	1.00
cg13496098	10	C10orf131	3.921E-15	2.43	19.15	7.87	0.90	0.79	1.00
cg15819128	2	LRRFIP1	2.116E-49	2.45	44.78	18.31	0.90	0.79	1.00
cg15871215	5	ATG10	2.453E-28	0.47	26.80	56.42	0.90	0.79	1.00
cg17154187	17	SMCR7	0.0029515	0.35	1.48	4.22	0.90	0.79	1.00
cg20080247	8	TG	5.063E-44	2.67	27.72	10.38	0.90	0.79	1.00

cg22806557	3	PIK3CA	4.973E-43	2.97	23.23	7.83	0.90	0.79	1.00
cg23970275	2	KLF7	4.381E-47	2.45	38.49	15.69	0.90	0.79	1.00
cg24903527	10	PDCD4	2.391E-10	2.05	17.73	8.63	0.90	0.79	1.00
cg26010412	14	MDGA2	4.797E-26	0.44	33.07	74.42	0.90	0.79	1.00
ch.1.4885626R	1	HNRNPU	5.579E-45	2.57	31.34	12.21	0.90	0.79	1.00
cg00554702	11	CHEK1	1.133E-52	2.87	47.70	16.60	0.90	0.79	1.00
cg01838443	21	HSPA13	1.881E-25	0.36	24.81	69.55	0.90	0.79	1.00
cg03572772	5	PCDHB15	6.55E-15	2.23	21.27	9.55	0.90	0.79	1.00
cg05101437	7	CDK6	1.775E-45	2.02	39.71	19.68	0.90	0.79	1.00
cg09674502	1	GFI1	4.737E-25	0.45	14.32	32.06	0.90	0.79	1.00
cg13572892	9	LHX6	2.559E-08	2.13	13.80	6.47	0.90	0.79	1.00
cg14144771	14	SNORD114-15; SNORD114-16	1.081E-26	0.49	36.75	74.58	0.90	0.79	1.00
cg14860393	12	KIF21A	1.453E-26	0.46	32.49	71.00	0.90	0.79	1.00
cg15125763	2	VWC2L	8.934E-44	3.06	25.03	8.17	0.90	0.79	1.00
cg16373202	16	CDH3	6.245E-05	0.48	3.92	8.19	0.90	0.79	1.00
cg16678169	2	ALS2CR4	2.362E-08	2.19	13.30	6.06	0.90	0.79	1.00
cg18708075	19	ZNF175	2.847E-42	2.55	22.78	8.93	0.90	0.79	1.00
cg19252956	9	DOCK8	1.415E-43	2.48	27.60	11.13	0.90	0.79	1.00
cg21193484	14	PRKCH	3.319E-15	2.21	21.91	9.94	0.90	0.79	1.00
cg23517124	12	RACGAP1	0.0417713	0.43	1.49	3.44	0.90	0.79	1.00
cg25123566	12	FAM113B; LOC100233209	2.767E-12	2.36	16.69	7.06	0.90	0.79	1.00
cg25898092	12	TMEM233	1.301E-42	2.19	26.79	12.24	0.90	0.79	1.00
cg27308355	20	CSTF1;AURKA	1.886E-07	2.30	11.29	4.91	0.90	0.79	1.00
ch.1.3577855R	1	LAMC1	4.154E-11	2.09	18.26	8.72	0.90	0.79	1.00
ch.13.1524792R	13	DOCK9	1.886E-45	2.88	30.60	10.62	0.90	0.79	1.00
ch.15.663480R	15	LEO1	1.398E-42	2.36	25.13	10.64	0.90	0.79	1.00
ch.7.1637031F	7	GTF2I	4.733E-45	2.51	32.02	12.76	0.90	0.79	1.00
cg00061989	17	CDK12	3.15E-07	2.13	12.26	5.76	0.89	0.78	1.00
cg00922727	6	C6orf182;SESN1	8.267E-44	2.44	28.66	11.73	0.89	0.78	1.00
cg01179851	18	SALL3	3.29E-13	2.14	20.41	9.54	0.89	0.78	1.00
cg01336231	3	RFTN1	1.01E-44	2.82	28.88	10.23	0.89	0.78	1.00
cg01396855	19	ZNF225	7.891E-44	2.05	33.08	16.11	0.89	0.78	1.00
cg01878462	11	ETS1	3.827E-06	2.04	11.35	5.56	0.89	0.78	1.00
cg02829601	6	SYTL3	1.608E-13	2.15	20.71	9.65	0.89	0.78	1.00
cg03521113	12	LRMP	4.571E-46	3.29	30.27	9.21	0.89	0.78	1.00
cg05889842	3	FAM194A	0.0491716	2.05	4.01	1.96	0.89	0.78	1.00
cg06193958	12	HOXC10	6.966E-12	2.51	15.12	6.04	0.89	0.78	1.00
cg07307388	6	CMAH	1.035E-12	2.58	15.50	6.02	0.89	0.78	1.00
cg08269461	21	GART;SON	6.786E-43	2.32	26.56	11.43	0.89	0.78	1.00
cg09601770	2	DPP4	1.404E-05	0.49	4.57	9.37	0.89	0.78	1.00
cg11820270	14	KTN1	1.432E-26	0.43	29.42	67.90	0.89	0.78	1.00
cg14752838	7	FGL2;CCDC146	2.241E-42	3.26	20.30	6.23	0.89	0.78	1.00
cg16613143	17	METT10D	6.502E-10	0.38	4.09	10.78	0.89	0.78	1.00
cg17472953	4	SCLT1;C4orf33	2.672E-15	2.48	18.85	7.59	0.89	0.78	1.00
cg18158043	14	AK7	1.968E-07	2.09	12.95	6.20	0.89	0.78	1.00
cg19402236	13	FLT1	8.872E-44	2.20	30.88	14.01	0.89	0.78	1.00

cg21382890	2	NFE2L2	1.399E-42	3.53	20.21	5.72	0.89	0.78	1.00
cg26577454	16	CNTNAP4	3.407E-06	2.31	9.60	4.16	0.89	0.78	1.00
cg27510097	6	IRAK1BP1	4.852E-10	2.16	16.00	7.42	0.89	0.78	1.00
ch.11.975705R	11	C11orf49	3.59E-45	3.64	26.85	7.37	0.89	0.78	1.00
ch.12.1175666F	12	RNF41	2.87E-42	2.40	23.75	9.90	0.89	0.78	1.00
ch.2.2322915R	2	BUB1	1.661E-43	2.72	25.87	9.53	0.89	0.78	1.00
cg01074354	14	AKAP6	3.808E-27	0.48	32.13	67.59	0.89	0.77	1.00
cg02173849	5	PJA2	6.756E-44	2.72	27.01	9.91	0.89	0.77	1.00
cg03309180	12	PRICKLE1	1.088E-13	2.63	16.08	6.10	0.89	0.77	1.00
cg04880751	1	KMO	1.65E-43	2.14	30.68	14.33	0.89	0.77	1.00
cg06193723	12	AICDA	2.652E-48	2.51	41.22	16.44	0.89	0.77	1.00
cg07243941	4	CORIN	2.076E-10	2.36	14.53	6.15	0.89	0.77	1.00
cg07323919	7	ETV1	2.076E-42	2.23	25.62	11.48	0.89	0.77	1.00
cg09336228	16	DDX19A	0.001495	2.02	7.04	3.48	0.89	0.77	1.00
cg12396306	5	PCDHB14	1.758E-17	2.44	21.52	8.83	0.89	0.77	1.00
cg14640066	2	RTN4	6.187E-42	2.50	21.92	8.77	0.89	0.77	1.00
cg15019617	10	IFIT1	2.878E-13	2.32	18.20	7.83	0.89	0.77	1.00
cg16641411	2	CREB1	2.115E-44	2.08	34.80	16.74	0.89	0.77	1.00
cg16983211	12	HOXC4	0.0042617	0.43	2.07	4.83	0.89	0.77	1.00
cg19223299	6	PRDM13	1.323E-06	2.43	9.55	3.93	0.89	0.77	1.00
cg20752420	7	LOC100128542	2.188E-44	3.10	26.63	8.60	0.89	0.77	1.00
cg21140028	12	PLEKHA5	4.616E-27	0.49	34.70	70.60	0.89	0.77	1.00
cg26480862	22	TTC28	4.755E-13	2.06	21.52	10.46	0.89	0.77	1.00
cg26681912	1	SLC19A2	0.0014023	0.44	2.46	5.60	0.89	0.77	1.00
ch.18.196065F	18	KIAA0802	6.362E-16	2.18	23.11	10.60	0.89	0.77	1.00
ch.20.546216F	20	GINS1	1.186E-44	3.17	27.05	8.52	0.89	0.77	1.00
cg00544901	19	RPS11	2.366E-09	0.50	7.62	15.34	0.88	0.76	1.00
cg00635944	16	C16orf53	1.643E-06	2.40	9.58	4.00	0.88	0.76	1.00
cg01873391	8	MTDH	1.88E-42	2.21	26.01	11.78	0.88	0.76	1.00
cg07588263	8	C8orf40	0.0091089	0.47	2.33	4.92	0.88	0.76	1.00
cg07788438	17	MYO1D	8.382E-14	2.13	21.31	10.00	0.88	0.76	1.00
cg08637514	7	GIMAP7	4.807E-43	2.64	24.81	9.38	0.88	0.76	1.00
cg09364733	13	PDS5B	8.645E-27	0.49	36.37	73.69	0.88	0.76	1.00
cg10043253	17	SLC13A5	1.096E-06	2.02	12.46	6.17	0.88	0.76	1.00
cg11859594	17	CYTH1	6.918E-11	2.06	18.38	8.90	0.88	0.76	1.00
cg13169132	1	TMEM88B	1.042E-12	2.17	19.37	8.93	0.88	0.76	1.00
cg17861863	10	ADARB2	2.409E-14	2.03	23.83	11.76	0.88	0.76	1.00
cg18166947	22	BCL2L13	9.414E-12	2.15	18.36	8.53	0.88	0.76	1.00
cg21696208	8	MTFR1	2.6E-17	2.12	25.69	12.11	0.88	0.76	1.00
cg24467337	20	LOC284805	3.26E-42	2.42	23.42	9.68	0.88	0.76	1.00
cg26931437	1	SHE	1.216E-41	2.98	18.87	6.34	0.88	0.76	1.00
cg24881689	7	OR2A14	5.053E-42	2.84	20.57	7.23	0.88	0.76	1.00
cg02501186	17	FDXR	5.828E-11	2.14	17.48	8.18	0.88	0.75	1.00
cg03993154	7	SLC13A1	1.13E-13	2.12	21.31	10.05	0.88	0.75	1.00
cg04190784	20	C20orf199; SNORD12C	5.283E-05	0.41	2.84	6.86	0.88	0.75	1.00
cg04199473	14	STRN3	6.133E-45	2.42	32.42	13.37	0.88	0.75	1.00
cg04663487	7	TFEC	1.136E-44	2.21	33.96	15.40	0.88	0.75	1.00

cg05831784	20	HAO1	1.948E-14	2.25	20.44	9.09	0.88	0.75	1.00
cg06126019	11	DENND5A	2.339E-43	2.45	27.10	11.05	0.88	0.75	1.00
cg06329684	4	C4orf49	1.725E-48	2.59	40.82	15.76	0.88	0.75	1.00
cg07032258	11	ARNTL	5.376E-48	2.17	45.11	20.82	0.88	0.75	1.00
cg08146609	7	HDAC9	2.863E-43	2.16	29.57	13.69	0.88	0.75	1.00
cg08321576	12	CLEC2B	1.88E-45	2.96	30.16	10.18	0.88	0.75	1.00
cg09762252	7	CALN1	2.191E-44	2.18	33.26	15.24	0.88	0.75	1.00
cg09859456	12	TBC1D15	2.702E-41	3.32	16.85	5.07	0.88	0.75	1.00
cg09872926	10	EBF3	6.474E-45	2.79	29.64	10.63	0.88	0.75	1.00
cg10134889	3	IFT57	6.876E-27	0.46	31.97	68.77	0.88	0.75	1.00
cg10227024	17	SHISA6	8.325E-48	2.39	41.22	17.24	0.88	0.75	1.00
cg10272675	15	MFGE8	0.0236357	0.46	1.91	4.12	0.88	0.75	1.00
cg13190924	15	SERF2	0.002532	0.44	2.33	5.28	0.88	0.75	1.00
cg13916266	17	CHD3	1.068E-44	2.83	28.80	10.19	0.88	0.75	1.00
cg13930596	12	TBX3	4.472E-45	2.29	34.25	14.95	0.88	0.75	1.00
cg15611279	5	LOC645323	0.0001429	2.24	7.68	3.43	0.88	0.75	1.00
cg16217908	3	C3orf52	2.174E-12	2.24	18.14	8.11	0.88	0.75	1.00
cg16818768	21	PSMG1	9.32E-09	2.36	12.55	5.32	0.88	0.75	1.00
cg18309286	11	PAK1	0.0123206	0.48	2.34	4.83	0.88	0.75	1.00
cg18587683	7	FLJ40852	5.105E-27	0.47	32.38	68.50	0.88	0.75	1.00
cg19140085	12	LMO3	3.966E-06	2.59	8.40	3.25	0.88	0.75	1.00
cg19610638	6	HIST1H3E	1.421E-48	2.39	43.35	18.14	0.88	0.75	1.00
cg25143235	1	CHD5	9.133E-12	2.76	13.53	4.90	0.88	0.75	1.00
cg25918541	11	ATM;NPAT	4.397E-41	4.00	15.09	3.77	0.88	0.75	1.00
cg26014538	3	ZIC1	1.102E-43	2.82	25.85	9.16	0.88	0.75	1.00
cg26397265	12	LYRM5;CASC1	0.0012758	0.48	3.07	6.36	0.88	0.75	1.00
ch.13.24413F	13	ZMYM5	1.392E-42	2.35	25.23	10.73	0.88	0.75	1.00
ch.18.658499F	18	RPRD1A	2.002E-16	2.06	25.91	12.59	0.88	0.75	1.00
ch.20.773168F	20	CTNNBL1	2.987E-43	3.10	23.38	7.54	0.88	0.75	1.00
ch.3.1381988F	3	CADPS	3.652E-42	2.40	23.35	9.72	0.88	0.75	1.00
ch.8.1960908F	8	PTDSS1	7.911E-48	3.37	34.13	10.12	0.88	0.75	1.00
ch.9.2043201R	9	GAPVD1	2.88E-15	2.13	23.15	10.87	0.88	0.75	1.00
cg20765267	8	DCAF13;SLC25A32	3.695E-43	2.50	26.13	10.47	0.87	0.75	1.00
ch.1.930581F	1	RPA2	1.473E-46	3.43	30.91	9.00	0.87	0.75	1.00
cg01803886	3	CNTN4	5.259E-29	0.50	26.25	52.80	0.87	0.74	0.99
cg03143531	1	RBBP5	4.834E-17	2.53	20.07	7.93	0.87	0.74	0.99
cg03804847	2	PCBP1	2.038E-43	2.12	30.60	14.43	0.87	0.74	0.99
cg04149335	14	THTPA	0.026141	2.07	4.51	2.18	0.87	0.74	0.99
cg05587853	3	MSL2	0.0239056	0.48	2.03	4.25	0.87	0.74	0.99
cg06161698	22	LOC400927	1.618E-17	2.34	22.46	9.59	0.87	0.74	0.99
cg14077219	6	RPF2	2.384E-17	2.08	26.57	12.80	0.87	0.74	0.99
cg15896259	8	ENTPD4	1.01E-14	2.02	24.41	12.06	0.87	0.74	0.99
cg17042946	3	MED12L;P2RY14	2.052E-17	2.52	20.46	8.12	0.87	0.74	0.99
cg19519331	10	PANK1	2.352E-10	2.39	14.25	5.97	0.87	0.74	0.99
ch.11.966965R	11	CKAP5	2.996E-44	2.75	27.95	10.18	0.87	0.74	0.99
ch.19.1899367R	19	ZIK1	9.75E-13	2.20	18.99	8.63	0.87	0.74	0.99
ch.8.1247165R	8	LYN	8.45E-13	2.11	20.29	9.60	0.87	0.74	0.99
cg07347725	9	MORN5;NDUFA8	4.967E-16	2.18	23.30	10.70	0.87	0.74	0.99

cg15846718	6	COX7A2	9.555E-12	2.29	16.79	7.34	0.87	0.74	0.99
cg23327164	19	ZNF266	1.189E-42	2.35	25.45	10.82	0.87	0.74	0.99
ch.15.1310449F	15	MORF4L1	3.476E-47	3.70	31.46	8.49	0.87	0.74	0.99
ch.6.1274077F	6	C6orf142	1.736E-47	2.82	36.35	12.89	0.87	0.74	0.99
cg18855030	2	ARHGAP15	1.67E-42	2.28	25.56	11.23	0.87	0.74	0.99
cg00454770	10	ABLIM1	9.249E-18	2.04	27.64	13.52	0.86	0.74	0.99
cg00933153	20	C20orf56	5.158E-46	3.36	29.87	8.90	0.86	0.74	0.99
cg01086446	5	GABRA1	2.485E-44	2.25	32.29	14.36	0.86	0.74	0.99
cg02061956	1	FMO5	1.938E-17	2.49	20.84	8.37	0.86	0.74	0.99
cg05257202	13	UBAC2	1.829E-42	2.23	25.85	11.60	0.86	0.74	0.99
cg05530568	5	EBF1	7.577E-47	2.37	38.77	16.37	0.86	0.74	0.99
cg06197769	12	CDKN1B	1.87E-10	2.21	15.92	7.19	0.86	0.74	0.99
cg06360703	2	LASS6	2.001E-43	2.48	27.11	10.93	0.86	0.74	0.99
cg06457633	10	APBB1IP	0.0002216	2.05	8.34	4.07	0.86	0.74	0.99
cg09866743	3	ARPP-21	2.751E-43	2.05	31.02	15.11	0.86	0.74	0.99
cg10841831	10	FANK1	5.082E-10	2.12	16.37	7.71	0.86	0.74	0.99
cg11229610	12	MLL2;PRKAG1	1.725E-09	2.07	16.32	7.90	0.86	0.74	0.99
cg12415720	13	C13orf23;NHLRC3	2.145E-13	2.19	19.93	9.09	0.86	0.74	0.99
cg14278321	9	PRDM12	4.777E-10	2.01	17.93	8.92	0.86	0.74	0.99
cg16658931	19	SHANK1	6.214E-09	0.45	5.63	12.46	0.86	0.74	0.99
cg18516609	10	ZEB1	6.801E-27	0.49	35.18	71.95	0.86	0.74	0.99
cg21550141	2	MAP4K4	8.657E-15	2.34	19.71	8.41	0.86	0.74	0.99
cg22813140	3	DNAJC19	7.641E-14	2.53	16.99	6.72	0.86	0.74	0.99
cg23649161	17	AARSD1;RUNDC1	0.0238653	0.46	1.83	4.03	0.86	0.74	0.99
cg24112733	7	STARD3NL	2.136E-42	2.51	23.48	9.37	0.86	0.74	0.99
cg24856456	8	LYN;LYN	8.351E-18	2.00	28.65	14.30	0.86	0.74	0.99
cg26266862	1	ZMPSTE24	3.141E-06	2.04	11.49	5.63	0.86	0.74	0.99
cg26315984	1	TNFSF4	4.372E-42	2.32	23.64	10.17	0.86	0.74	0.99
ch.14.1251344R	14	GTF2A1	1.614E-44	2.50	30.50	12.22	0.86	0.74	0.99
ch.2.1056241F	2	LRPPRC	8.269E-44	2.53	27.96	11.03	0.86	0.74	0.99
ch.3.3938922F	3	LRCH3	3.831E-43	2.43	26.54	10.91	0.86	0.74	0.99
ch.5.389453F	5	ANKH	6.434E-43	2.85	23.38	8.20	0.86	0.74	0.99
ch.2.2007613R	2	CIAO1	8.511E-46	2.70	32.74	12.15	0.86	0.73	0.99
cg00296158	1	POU2F1	6.939E-15	2.20	21.68	9.88	0.86	0.73	0.99
cg01853638	2	INO80D	2.131E-42	2.19	26.02	11.91	0.86	0.73	0.99
cg02725370	4	PITX2	6.488E-05	2.09	8.99	4.30	0.86	0.73	0.99
cg03580106	16	ZNF778	1.993E-10	2.03	18.24	8.99	0.86	0.73	0.99
cg04294990	17	USP22	3.874E-43	2.42	26.65	11.03	0.86	0.73	0.99
cg04391722	8	ZBTB10	8.819E-43	2.17	27.60	12.70	0.86	0.73	0.99
cg04405266	1	IFI6	1.136E-10	2.20	16.31	7.40	0.86	0.73	0.99
cg06747888	2	TBR1	2.788E-42	2.52	23.00	9.12	0.86	0.73	0.99
cg08172552	15	TMEM87A;GANC	9.882E-46	2.96	30.91	10.43	0.86	0.73	0.99
cg08966155	3	RPL35A;IQCG	6.379E-13	2.53	16.07	6.36	0.86	0.73	0.99
cg10717387	6	HIST1H1C	5.427E-10	2.11	16.51	7.83	0.86	0.73	0.99
cg10900715	6	C6orf130	1.158E-15	2.09	24.39	11.69	0.86	0.73	0.99
cg11756029	12	TRAFD1	3.287E-43	2.34	27.50	11.74	0.86	0.73	0.99
cg13309012	21	MIR155HG;MIR155	2.482E-44	2.05	35.05	17.13	0.86	0.73	0.99
cg15912800	7	MIR196B	0.0047016	0.48	2.57	5.40	0.86	0.73	0.99

cg20596240	15	RNF111	8.05E-43	2.79	23.34	8.36	0.86	0.73	0.99
cg21123203	4	CC2D2A	3.495E-12	2.03	20.82	10.26	0.86	0.73	0.99
cg23488414	4	ANKRD17	2.191E-44	2.20	32.99	14.97	0.86	0.73	0.99
cg24221919	7	PTPRN2	3.979E-47	2.18	42.27	19.40	0.86	0.73	0.99
cg24861399	2	MGAT5	2.535E-16	2.02	26.53	13.13	0.86	0.73	0.99
cg25993851	11	ARHGAP1;ZNF408	3.494E-05	0.48	4.20	8.67	0.86	0.73	0.99
ch.11.1613249R	11	RNF169	1.822E-07	2.04	13.50	6.62	0.86	0.73	0.99
ch.4.1889364R	4	EIF4E	1.245E-12	2.17	19.26	8.87	0.86	0.73	0.99
ch.5.2679849F	5	ARHGAP26	2.338E-43	2.82	24.86	8.81	0.86	0.73	0.99
cg03252340	4	HNRNPD	2.043E-14	2.95	15.03	5.09	0.86	0.72	0.99
cg03897866	1	IER5	9.857E-11	2.14	17.14	8.01	0.85	0.72	0.99
cg08252887	10	CWF19L1	0.0038212	2.10	5.97	2.84	0.85	0.72	0.99
cg12867312	7	BRAF	7.337E-28	0.49	30.80	62.69	0.85	0.72	0.99
cg14025652	1	GPR177	7.887E-43	2.01	29.89	14.89	0.85	0.72	0.99
cg16882226	2	CHST10	0.0170098	0.47	2.09	4.43	0.85	0.72	0.99
cg21428833	6	LMBRD1	3.892E-07	2.12	12.19	5.75	0.85	0.72	0.99
cg22437074	2	SIX3	3.617E-06	2.30	9.59	4.16	0.85	0.72	0.99
cg24587175	10	OBFC1	2.682E-17	2.14	25.35	11.84	0.85	0.72	0.99
cg26522946	10	SRGN	3.767E-15	2.84	16.20	5.70	0.85	0.72	0.99
cg00591160	12	ING4	4.012E-43	2.06	30.24	14.66	0.85	0.72	0.99
cg02717454	16	CREBBP	3.37E-17	2.00	28.05	14.02	0.85	0.72	0.99
cg10934032	14	RNASE6	2.533E-13	2.17	20.11	9.26	0.85	0.72	0.99
cg12386061	3	CTDSPL	1.129E-46	2.76	34.70	12.59	0.85	0.72	0.99
cg12595461	2	VIT	1.214E-15	2.38	20.20	8.48	0.85	0.72	0.99
cg13071672	1	DYRK3	6.373E-42	2.49	21.93	8.80	0.85	0.72	0.99
cg15023571	2	STK17B	3.374E-44	2.49	29.51	11.83	0.85	0.72	0.99
cg15621971	7	H2AFV	1.258E-12	2.22	18.61	8.38	0.85	0.72	0.99
cg17074863	4	KLF3	4.273E-43	2.11	29.57	14.04	0.85	0.72	0.99
cg26593946	15	NR2F2	5.633E-16	2.00	26.51	13.25	0.85	0.72	0.99
cg15855450	6	HIST1H4C	4.717E-44	2.45	29.43	12.04	0.85	0.72	0.99
cg18637383	2	HEATR5B	2.439E-45	2.75	31.10	11.32	0.85	0.72	0.99
cg24330922	6	MRPS18B; PPP1R10	8.36E-44	2.74	26.67	9.75	0.85	0.72	0.99
cg03049243	19	PRPF31;TFPT	6.719E-08	0.31	2.37	7.58	0.85	0.71	0.99
cg03465028	15	KLF13	5.487E-13	2.38	17.31	7.26	0.85	0.71	0.99
cg03750742	1	ASPM	1.397E-42	2.19	26.69	12.20	0.85	0.71	0.99
cg03830585	3	ITPR1	2.31E-42	2.27	25.14	11.10	0.85	0.71	0.99
cg04541368	5	FLJ42709	4.441E-06	2.14	10.49	4.91	0.85	0.71	0.99
cg05340255	1	DNAJB4	1.542E-11	2.01	20.25	10.09	0.85	0.71	0.99
cg06097216	3	CNBP	1.16E-44	2.69	29.51	10.97	0.85	0.71	0.99
cg06240690	2	GALNT5	7.568E-44	2.19	31.31	14.31	0.85	0.71	0.99
cg07648207	18	C18orf10	2.446E-43	2.27	28.57	12.56	0.85	0.71	0.99
cg08406370	16	PRKCB	0.0001336	0.45	3.12	7.00	0.85	0.71	0.99
cg08771429	7	KRIT1	6.757E-12	2.14	18.70	8.73	0.85	0.71	0.99
cg10439431	17	CCL23;CCL23	7.322E-43	2.54	24.86	9.79	0.85	0.71	0.99
cg13885748	9	PTPN3	1.28E-42	2.28	25.98	11.41	0.85	0.71	0.99
cg14815005	22	MAPK1	0.0069629	0.48	2.48	5.17	0.85	0.71	0.99
cg15739181	3	DCUN1D1	5.051E-16	2.40	20.37	8.47	0.85	0.71	0.99

cg16741573	18	GALNT1	2.348E-46	2.01	42.84	21.27	0.85	0.71	0.99
cg18387085	18	ZADH2;TSHZ1	1.143E-42	2.93	22.27	7.60	0.85	0.71	0.99
cg19005662	14	TTC5	3.137E-05	2.03	9.90	4.87	0.85	0.71	0.99
cg22748740	6	ZNF311	0.0020803	2.34	5.66	2.42	0.85	0.71	0.99
cg23712405	1	BTBD8	0.0002143	2.02	8.52	4.21	0.85	0.71	0.99
cg24402880	4	PLAC8	1.332E-14	2.06	23.46	11.37	0.85	0.71	0.99
cg25537846	11	DDX25;PUS3	2.106E-07	2.25	11.57	5.14	0.85	0.71	0.99
cg26937809	1	CENPL;DARS2	0.0088476	2.35	4.68	1.99	0.85	0.71	0.99
cg27316970	19	LGI4	0.0079166	0.47	2.36	4.99	0.85	0.71	0.99
ch.17.1360189F	17	CA10	2.577E-43	2.91	24.34	8.38	0.85	0.71	0.99
ch.17.958355F	17	DDX52	9.617E-44	2.34	29.30	12.50	0.85	0.71	0.99
ch.20.393029F	20	SEC23B	6.709E-44	2.59	27.84	10.74	0.85	0.71	0.99
ch.7.128681R	7	C7orf27	3.493E-15	2.76	16.72	6.07	0.85	0.71	0.99
cg22178096	3	RPL32P3	2.988E-09	2.44	12.62	5.17	0.85	0.71	0.98
ch.9.2285199R	9	MED27	2.211E-15	2.37	20.05	8.46	0.85	0.71	0.98
cg01896926	17	GLOD4;RNMTL1	0.0006007	0.44	2.66	6.06	0.84	0.70	0.98
cg02794358	2	SPTBN1	1.74E-42	2.19	26.32	12.02	0.84	0.70	0.98
cg04099562	1	WDR26	5.829E-45	2.20	34.97	15.88	0.84	0.70	0.98
cg04620291	8	POLR2K	3.786E-11	2.23	16.70	7.50	0.84	0.70	0.98
cg07863159	22	MLC1	7.182E-14	2.10	21.83	10.38	0.84	0.70	0.98
cg07889938	4	AFF1	5.734E-44	3.19	25.12	7.88	0.84	0.70	0.98
cg11969526	2	PIKFYVE	1.829E-45	2.11	38.10	18.10	0.84	0.70	0.98
cg12236045	10	CTNNA3;LRRTM3	8.32E-42	2.56	21.15	8.27	0.84	0.70	0.98
cg18317589	6	PNRC1	1.842E-13	2.67	15.62	5.84	0.84	0.70	0.98
cg21594702	5	VCAN	2.329E-44	2.07	34.85	16.87	0.84	0.70	0.98
cg24374161	11	AMBRA1	2.179E-42	2.40	24.17	10.07	0.84	0.70	0.98
cg24833674	1	ILDR2	3.239E-45	2.27	35.00	15.45	0.84	0.70	0.98
cg24840062	3	CDCP1	1.469E-42	2.21	26.40	11.95	0.84	0.70	0.98
ch.1.1416966F	1	GPBP1L1	1.856E-45	2.42	34.03	14.04	0.84	0.70	0.98
ch.10.2007128F	10	HELLS	3.048E-43	2.38	27.25	11.43	0.84	0.70	0.98
ch.13.601939F	13	COG3	2.03E-46	2.96	32.73	11.05	0.84	0.70	0.98
ch.15.1525362R	15	ABHD2	1.581E-15	2.46	19.33	7.87	0.84	0.70	0.98
ch.17.795614F	17	ATAD5	7.401E-44	2.87	26.11	9.09	0.84	0.70	0.98
ch.6.2949012F	6	ESR1	1.666E-42	2.59	23.35	9.02	0.84	0.70	0.98
cg23942268	17	FAM64A	8.431E-06	0.37	2.49	6.81	0.84	0.70	0.98
cg00064255	1	LRRC8C	1.853E-14	3.87	12.35	3.19	0.84	0.70	0.98
cg00396484	11	KIRREL3	9.484E-11	2.11	17.61	8.36	0.84	0.70	0.98
cg04308224	19	RPL18;SPHK2	0.0033615	0.46	2.50	5.40	0.84	0.70	0.98
cg05319212	16	NAE1	1.189E-16	2.58	19.35	7.50	0.84	0.70	0.98
cg08905629	1	LAMC2	9.579E-11	2.42	14.51	6.01	0.84	0.70	0.98
cg12941374	17	SKA2;PRR11	2.364E-07	2.23	11.62	5.20	0.84	0.70	0.98
cg13452400	2	CMPK2	0.0380842	0.45	1.64	3.64	0.84	0.70	0.98
cg15028160	19	PPFIA3;C19orf73	8.56E-14	0.44	7.61	17.41	0.84	0.70	0.98
cg16441347	6	FIG4	2.959E-27	0.49	33.72	68.63	0.84	0.70	0.98
cg17962342	12	HIP1R	0.0020081	2.13	6.32	2.97	0.84	0.70	0.98
cg23054925	1	SPATA1;GNG5	5.483E-42	2.50	22.13	8.86	0.84	0.70	0.98
cg26876444	1	LEPR;LEPROT	2.154E-43	2.18	29.79	13.67	0.84	0.70	0.98
cg27164797	6	BAG2	2.022E-17	2.01	28.08	13.94	0.84	0.70	0.98

cg27182172	4	PPARGC1A	3.871E-10	2.85	11.62	4.08	0.84	0.70	0.98
cg04219725	2	COQ10B	1.884E-15	2.01	25.63	12.75	0.84	0.70	0.98
cg09597585	12	MED21	4.855E-08	2.16	13.21	6.13	0.84	0.70	0.98
cg10638657	6	HIST1H1D	6.579E-47	2.56	36.91	14.41	0.84	0.70	0.98
cg15575982	1	LMO4	2.876E-10	2.02	18.10	8.95	0.84	0.70	0.98
cg19305488	19	APC2	8.159E-15	2.31	20.10	8.70	0.84	0.70	0.98
cg26633751	4	CCNI	2.174E-10	2.45	13.86	5.66	0.84	0.70	0.98
ch.5.2763962F	5	JAKMIP2	2.26E-44	2.75	28.29	10.29	0.84	0.70	0.98
cg00240653	12	KIAA0748	1.845E-17	2.54	20.75	8.17	0.84	0.69	0.98
cg10005998	10	DNMBP	5.858E-42	2.59	21.53	8.32	0.84	0.69	0.98
cg26689203	4	MYOZ2	7.974E-43	2.63	24.18	9.19	0.84	0.69	0.98
cg00826203	1	SCMH1	1.633E-43	2.06	31.73	15.38	0.83	0.69	0.98
cg02075820	11	NUCB2	2.047E-45	2.28	35.50	15.58	0.83	0.69	0.98
cg04803572	17	C17orf81;DULLARD	0.005905	0.42	1.89	4.53	0.83	0.69	0.98
cg05527034	4	DCHS2	2.624E-15	2.35	20.24	8.63	0.83	0.69	0.98
cg08586426	11	LMO2	9.086E-09	2.19	13.86	6.32	0.83	0.69	0.98
cg10086030	11	EED	4.292E-11	2.84	12.52	4.40	0.83	0.69	0.98
cg10651618	6	RRAGD	0.0011273	0.47	2.91	6.20	0.83	0.69	0.98
cg12103037	14	NKX2-1	1.368E-42	2.16	26.98	12.47	0.83	0.69	0.98
cg13634151	19	EEF2	1.514E-08	2.09	14.65	7.02	0.83	0.69	0.98
cg17160660	8	MYC	2.278E-45	2.25	35.72	15.89	0.83	0.69	0.98
cg18945861	14	TTLL5;C14orf1	6.736E-44	2.05	33.37	16.27	0.83	0.69	0.98
cg20716209	17	STAT3	2.197E-10	2.02	18.27	9.04	0.83	0.69	0.98
cg21172319	8	NAT1	3.533E-08	2.18	13.16	6.03	0.83	0.69	0.98
cg21410633	1	CAP1	4.228E-42	2.29	23.95	10.45	0.83	0.69	0.98
ch.3.3584022R	3	ABCC5	3.114E-13	2.37	17.67	7.44	0.83	0.69	0.98
cg11713788	6	SLC22A23	4.232E-17	2.12	25.61	12.10	0.83	0.69	0.98
cg00666915	17	C17orf63	5.794E-13	2.14	20.09	9.38	0.83	0.68	0.97
cg03619586	6	CLIC1	1.751E-15	2.10	23.86	11.34	0.83	0.68	0.97
cg05729499	6	HIST1H2AL	4.099E-09	2.37	12.92	5.45	0.83	0.68	0.97
cg09244489	4	FAM13A	1.372E-49	2.60	43.50	16.75	0.83	0.68	0.97
cg11028769	6	CMAH;CMAH	2.317E-17	2.66	19.35	7.29	0.83	0.68	0.97
cg12527995	1	FAM159A	5.78E-43	2.35	26.59	11.33	0.83	0.68	0.97
cg13624631	14	DHRS7	2.792E-16	2.76	17.66	6.39	0.83	0.68	0.97
cg13697193	12	GPR19	0.0004483	0.36	1.86	5.15	0.83	0.68	0.97
cg16023991	13	TSC22D1	1.962E-27	0.50	33.47	67.48	0.83	0.68	0.97
cg17503995	13	SPATA13	1.2E-08	2.41	12.10	5.02	0.83	0.68	0.97
cg17589633	1	ATP2B4	1.73E-43	2.52	27.03	10.72	0.83	0.68	0.97
cg17993442	11	ZBTB44	8.794E-44	2.57	27.62	10.74	0.83	0.68	0.97
cg18057559	3	BBX	4.433E-12	2.21	18.06	8.17	0.83	0.68	0.97
cg18789918	13	SLITRK1	1.513E-45	2.98	30.33	10.18	0.83	0.68	0.97
cg19180624	2	HOXD1	0.03691	0.50	2.04	4.11	0.83	0.68	0.97
cg25962286	2	DLX2	0.0001048	2.08	8.68	4.17	0.83	0.68	0.97
cg27370993	2	PTPN4	5.191E-42	2.48	22.31	8.99	0.83	0.68	0.97
ch.15.1497565F	15	NTRK3	8.387E-43	3.24	21.63	6.68	0.83	0.68	0.97
cg24662666	10	LOC282997	1.416E-44	3.12	27.06	8.68	0.83	0.68	0.97
ch.18.4153F	18	USP14	2.304E-46	2.99	32.41	10.83	0.83	0.68	0.97
ch.21.507718F	21	DYRK1A	1.193E-42	2.46	24.66	10.03	0.83	0.68	0.97

cg02559423	3	PRKCD	0.0018	2.38	5.63	2.36	0.82	0.67	0.97
cg05825244	20	EBF4	0.000973	2.38	6.00	2.52	0.82	0.67	0.97
cg10364968	3	CASR	1.32E-43	2.15	30.87	14.33	0.82	0.67	0.97
cg10478867	7	SMARCD3	6.16E-05	2.39	7.62	3.19	0.82	0.67	0.97
cg12163407	12	GDF3	5.761E-17	2.28	22.78	9.98	0.82	0.67	0.97
cg12423311	7	DPP6	1.884E-17	2.55	20.60	8.07	0.82	0.67	0.97
cg18302582	14	CLMN	0.0002552	2.23	7.36	3.30	0.82	0.67	0.97
cg18484665	2	THUMPD2	2.785E-43	2.48	26.64	10.74	0.82	0.67	0.97
cg19060550	6	NRN1	1.782E-46	3.07	32.29	10.52	0.82	0.67	0.97
cg20521702	3	ST3GAL6	4.467E-44	2.28	31.09	13.65	0.82	0.67	0.97
cg24443054	6	ZDHHC14	0.0076782	0.40	1.67	4.18	0.82	0.67	0.97
cg25034941	13	LRCH1	3.832E-14	2.08	22.57	10.84	0.82	0.67	0.97
cg25035059	8	CHD7	5.811E-43	2.37	26.43	11.17	0.82	0.67	0.97
cg25831075	3	SLITRK3	1.431E-42	2.14	27.18	12.71	0.82	0.67	0.97
ch.1.3571292R	1	DHX9	6.306E-46	2.21	38.00	17.18	0.82	0.67	0.97
ch.3.55501R	3	TRNT1	2.09E-50	2.32	49.19	21.23	0.82	0.67	0.97
cg26092471	5	MSX2	2.188E-12	2.12	19.61	9.24	0.82	0.67	0.97
ch.2.1685296R	2	MTHFD2	2.283E-42	2.46	23.68	9.62	0.82	0.67	0.97
cg18557215	3	GUCA1C	2.476E-11	2.08	18.82	9.06	0.82	0.67	0.97
cg02774862	10	TET1	1.322E-10	2.13	17.04	7.99	0.82	0.67	0.97
cg10308629	7	BPGM	7.236E-10	2.16	15.71	7.27	0.82	0.67	0.97
cg14446615	5	OTP	1.114E-13	2.17	20.63	9.52	0.82	0.67	0.97
cg16203607	17	DDX5	3.122E-47	2.04	45.27	22.23	0.82	0.67	0.97
cg17752684	20	FLRT3;MACROD2	2.408E-13	2.06	21.79	10.55	0.82	0.67	0.97
cg18652683	7	AOAH	1.057E-09	2.15	15.62	7.27	0.82	0.67	0.97
cg21395723	22	GTPBP1	3.372E-05	2.18	8.91	4.10	0.82	0.67	0.97
cg21438527	6	HIST1H4D	9.84E-42	2.55	20.92	8.19	0.82	0.67	0.97
cg22355889	11	ELMOD1;LOC643923	3.171E-16	2.26	22.28	9.84	0.82	0.67	0.97
cg24107163	1	FAIM3	4.601E-43	2.72	24.46	8.99	0.82	0.67	0.97
cg26215849	13	MTRF1	8.753E-43	2.37	25.79	10.89	0.82	0.67	0.97
ch.10.2186853R	10	NOLC1	1.642E-08	2.52	11.35	4.50	0.82	0.67	0.97
ch.1.2435132F	1	HIAT1	1.184E-14	2.29	20.19	8.82	0.82	0.66	0.97
cg00186034	13	UFM1	0.0015065	0.46	2.71	5.88	0.81	0.66	0.97
cg02030187	1	NHLH2	4.439E-42	2.32	23.66	10.20	0.81	0.66	0.97
cg02404636	22	SFI1	5.771E-16	2.28	21.80	9.58	0.81	0.66	0.97
cg03892631	1	RGS4	3.859E-44	2.08	33.89	16.33	0.81	0.66	0.97
cg06206957	7	NT5C3	8.429E-44	2.63	27.27	10.36	0.81	0.66	0.97
cg07011945	6	PSMB1	1.191E-10	2.13	17.19	8.08	0.81	0.66	0.97
cg15002204	6	CENPQ;MUT	9.09E-10	2.05	16.97	8.29	0.81	0.66	0.97
cg16842643	4	CLCN3	5.513E-14	2.11	21.84	10.34	0.81	0.66	0.97
cg19906926	4	KLHL5	5.691E-17	2.29	22.77	9.94	0.81	0.66	0.97
cg25707676	7	DLX6AS	6.996E-16	2.09	24.60	11.77	0.81	0.66	0.97
ch.7.2864275F	7	CREB3L2	3.831E-46	2.88	32.48	11.29	0.81	0.66	0.97
ch.8.969355F	8	SFRP1	5.326E-15	2.63	17.41	6.63	0.81	0.66	0.97
ch.10.1947223F	10	BTAF1	5.564E-44	2.62	27.94	10.68	0.81	0.65	0.96
ch.2.1294774R	2	CCDC88A	3.743E-44	2.65	28.27	10.68	0.81	0.65	0.96
cg04533487	3	UBA5;ACAD11	0.0089009	0.49	2.57	5.20	0.81	0.65	0.96
cg04785900	8	KBTBD11	6.734E-09	2.09	15.14	7.24	0.81	0.65	0.96

cg05794695	1	CENPF	7.149E-43	2.25	27.15	12.07	0.81	0.65	0.96
cg10503334	11	BCO2	1.127E-06	2.06	12.00	5.82	0.81	0.65	0.96
cg13062935	11	MRGPRX2	7.66E-14	2.06	22.46	10.88	0.81	0.65	0.96
cg13504245	9	NTRK2	1.485E-13	2.20	19.98	9.07	0.81	0.65	0.96
cg18792022	1	FBXO2	3.732E-16	2.02	26.38	13.08	0.81	0.65	0.96
ch.10.176154R	10	C10orf18	4.583E-44	2.22	31.65	14.23	0.81	0.65	0.96
ch.2.113772948R	2	LOC440839	3.337E-15	2.20	22.01	10.01	0.81	0.65	0.96
cg01738022	16	ADCY9	1.827E-44	2.72	28.71	10.54	0.81	0.65	0.96
cg09994323	2	FOXN2	9.528E-43	2.33	25.96	11.13	0.81	0.65	0.96
cg24918705	5	PCDHB4	5.699E-17	2.27	22.95	10.13	0.81	0.65	0.96
cg00672507	8	HGSNAT	1.707E-09	2.05	16.58	8.11	0.80	0.64	0.96
cg01247537	6	HIST1H4L	1.076E-44	2.14	34.85	16.25	0.80	0.64	0.96
cg03002480	15	UBR1	7.548E-05	2.64	6.77	2.56	0.80	0.64	0.96
cg03065625	11	LRP4	4.48E-09	2.02	16.27	8.05	0.80	0.64	0.96
cg12420683	7	KIAA0087	1.217E-45	2.10	38.76	18.45	0.80	0.64	0.96
cg13577505	22	MAPK11	5.071E-07	2.05	12.66	6.17	0.80	0.64	0.96
cg14617041	5	TMEM171	0.0010696	2.02	7.32	3.62	0.80	0.64	0.96
cg19488505	12	SCNN1A	1.616E-44	2.67	29.21	10.94	0.80	0.64	0.96
cg22568403	5	LOC257358	5.353E-17	2.24	23.41	10.44	0.80	0.64	0.96
cg22741595	11	C11orf67;RSF1	6.392E-48	2.26	43.33	19.16	0.80	0.64	0.96
cg23148651	6	SOX4	6.61E-43	2.32	26.61	11.45	0.80	0.64	0.96
cg23912435	1	ENSA	4.952E-10	2.03	17.57	8.64	0.80	0.64	0.96
cg27353361	6	SCML4	9.115E-09	2.24	13.44	5.99	0.80	0.64	0.96
cg27560444	1	H3F3A;LOC440926	6.426E-17	2.13	25.11	11.81	0.80	0.64	0.96
ch.10.2545310R	10	EIF3A	7.957E-44	2.41	29.03	12.07	0.80	0.64	0.96
ch.14.624270R	14	DDHD1	4.168E-11	2.08	18.49	8.90	0.80	0.64	0.96
ch.5.754313F	5	RAI14	1.215E-42	2.56	24.00	9.38	0.80	0.64	0.96
cg01083093	1	C1orf162	2.157E-42	2.26	25.32	11.22	0.80	0.64	0.96
cg01768246	1	REG4	2.214E-43	2.42	27.43	11.33	0.80	0.64	0.96
cg02329767	5	STARD4	0.0004761	2.17	7.20	3.32	0.80	0.64	0.96
cg06503907	6	SYCP2L	0.0207454	0.49	2.16	4.46	0.80	0.64	0.96
cg07856295	2	VSNL1	7.868E-43	2.43	25.46	10.46	0.80	0.64	0.96
cg08956138	11	CD3E	2.26E-07	2.00	13.78	6.88	0.80	0.64	0.96
cg09735598	1	ARPC5;RGL1	7.25E-43	2.33	26.39	11.32	0.80	0.64	0.96
cg16642281	4	LEF1	2.82E-08	2.17	13.41	6.18	0.80	0.64	0.96
cg25596215	5	PCDHB16	3.815E-44	2.01	35.00	17.42	0.80	0.64	0.96
ch.15.465126R	15	ZFP106	2.52E-10	2.27	15.25	6.73	0.80	0.64	0.96
ch.18.486455F	18	TAF4B	1.94E-08	2.13	13.97	6.55	0.80	0.64	0.96
ch.6.115952F	6	PRPF4B	3.433E-15	2.10	23.55	11.21	0.80	0.64	0.96
cg00908027	16	USP10	1.044E-16	2.12	25.08	11.84	0.79	0.63	0.96
cg02653559	3	ZIC4	1.363E-08	2.54	11.34	4.47	0.79	0.63	0.96
cg02260340	6	RNF5P1;RNF5; AGPAT1	0.0325608	2.19	4.05	1.85	0.79	0.63	0.95
cg02343814	11	KIF18A;METT5D1	1.75E-43	2.52	26.99	10.69	0.79	0.63	0.95
cg02491717	1	PIP5K1A	9.319E-16	2.02	25.83	12.79	0.79	0.63	0.95
cg06653140	5	SKP2	2.693E-44	2.09	34.24	16.39	0.79	0.63	0.95
cg09102714	16	AARS	2.195E-14	2.28	20.01	8.78	0.79	0.63	0.95
cg15888290	2	EN1	7.48E-14	2.16	20.89	9.66	0.79	0.63	0.95

cg23954819	12	BRI3BP	0.0086798	0.28	0.91	3.25	0.79	0.63	0.95
cg27415552	6	HIST1H2BE	3.631E-42	2.26	24.42	10.78	0.79	0.63	0.95
ch.5.1085870R	5	MAP3K1	3.74E-42	2.28	24.28	10.67	0.79	0.63	0.95
cg07027174	1	FAM102B	1.471E-45	2.46	33.99	13.82	0.79	0.63	0.95
cg20541456	5	CYFIP2	6.895E-17	2.02	27.16	13.44	0.79	0.63	0.95
cg19591950	19	ZNF221	1.28E-12	2.11	20.12	9.54	0.79	0.62	0.95
ch.2.2613291R	2	CNTNAP5	4.453E-44	3.61	24.13	6.69	0.79	0.62	0.95
cg03193589	3	FGD5	1.349E-17	2.22	24.15	10.86	0.79	0.62	0.95
cg08224069	6	BTN3A2	4.708E-43	2.90	23.56	8.12	0.79	0.62	0.95
cg13107060	4	C4orf33	1.29E-42	2.04	28.51	13.95	0.79	0.62	0.95
cg13270873	8	TRPS1	1.044E-06	2.11	11.65	5.53	0.79	0.62	0.95
cg14981132	12	HNRNPA1	1.108E-15	2.74	17.30	6.32	0.79	0.62	0.95
cg16114141	6	C6orf115	6.175E-42	2.56	21.56	8.41	0.79	0.62	0.95
cg17091793	13	FNDC3A	1.12E-17	2.16	25.54	11.84	0.79	0.62	0.95
cg17762331	12	ATN1	1.367E-13	2.16	20.66	9.58	0.79	0.62	0.95
cg18343292	11	MS4A7	5.865E-11	2.36	15.15	6.41	0.79	0.62	0.95
cg20198108	10	VIM	1.483E-12	2.19	18.94	8.66	0.79	0.62	0.95
cg23352695	17	NF1;EVI2A	9.322E-14	2.10	21.80	10.40	0.79	0.62	0.95
cg24120753	5	SV2C	2.354E-17	2.06	26.79	12.99	0.79	0.62	0.95
cg25653204	19	FPR2	1.207E-16	2.09	25.53	12.23	0.79	0.62	0.95
cg26708100	2	HOXD10	7.542E-09	3.17	9.52	3.01	0.79	0.62	0.95
ch.12.2619313F	12	TMED2	8.651E-16	2.09	24.43	11.68	0.79	0.62	0.95
cg25011749	13	C13orf38	1.327E-15	2.17	22.93	10.58	0.78	0.62	0.95
ch.5.731560F	5	ADAMTS12	1.967E-07	2.01	13.73	6.82	0.78	0.62	0.95
cg04605607	6	EEF1A1	1.265E-17	2.22	24.44	11.01	0.78	0.61	0.95
cg08085267	17	C17orf57	7.356E-15	2.16	22.17	10.26	0.78	0.61	0.95
cg09146577	10	LOC729020	1.337E-09	2.14	15.55	7.26	0.78	0.61	0.95
cg09652526	20	ZNF831	1.347E-17	2.26	23.85	10.57	0.78	0.61	0.95
cg11753867	7	IGFBP3	3.264E-07	2.70	9.20	3.40	0.78	0.61	0.95
cg14141775	3	RABL3;GTF2E1	3.519E-09	2.15	14.85	6.90	0.78	0.61	0.95
cg16563660	12	CDK17	3.073E-43	2.49	26.42	10.61	0.78	0.61	0.95
cg17478371	6	BRD2	8.007E-42	2.48	21.61	8.70	0.78	0.61	0.95
cg18219951	5	GABRG2	4.188E-11	2.17	17.26	7.95	0.78	0.61	0.95
cg18327669	14	SSTR1	7.368E-12	2.03	20.28	9.97	0.78	0.61	0.95
cg18956547	2	CXCR1	9.617E-11	2.30	15.44	6.70	0.78	0.61	0.95
cg26970477	19	EID2	1.007E-41	2.69	20.20	7.49	0.78	0.61	0.95
cg27168493	1	IL23R	2.482E-48	2.75	38.99	14.17	0.78	0.61	0.95
ch.16.1049690F	16	LONP2	8.668E-45	2.55	30.89	12.11	0.78	0.61	0.95
ch.18.189560F	18	RAB12	9.242E-45	2.29	33.21	14.48	0.78	0.61	0.95
ch.3.2410502F	3	PTPLB	1.455E-10	2.29	15.34	6.71	0.78	0.61	0.95
cg05601201	6	FOXO3	1.301E-46	2.14	41.24	19.24	0.78	0.61	0.95
cg11566977	4	MSX1	4.125E-16	3.11	15.72	5.06	0.78	0.61	0.95
ch.12.2061504F	12	TXNRD1	7.85E-45	3.40	26.70	7.85	0.78	0.61	0.95
cg00329300	1	TSTD1	0.0003963	0.50	3.76	7.51	0.78	0.61	0.95
cg01802545	14	LGMN	4.291E-15	2.04	24.54	12.02	0.78	0.61	0.95
cg01964262	13	MBNL2	1.532E-11	3.04	12.20	4.01	0.78	0.61	0.95
cg02667677	6	PRDM1	3.888E-15	2.42	19.23	7.93	0.78	0.61	0.95
cg05343811	14	NEK9	4.579E-17	2.16	24.79	11.46	0.78	0.61	0.95

cg06726167	17	TMEM107	3.423E-10	2.38	14.12	5.92	0.78	0.61	0.95
cg08375286	6	DAXX	5.764E-14	2.26	19.69	8.69	0.78	0.61	0.95
cg17507887	6	TFAP2B	2.251E-11	2.18	17.45	7.99	0.78	0.61	0.95
cg23507945	6	IL22RA2	7.618E-16	2.11	24.13	11.42	0.78	0.61	0.95
cg27159719	8	TMEM71	2.001E-17	2.57	20.29	7.90	0.78	0.61	0.95
ch.15.934240F	15	SNX1	3.707E-17	2.32	22.42	9.66	0.78	0.61	0.95
ch.3.183336F	3	SETD5	2.181E-16	2.50	19.79	7.91	0.78	0.61	0.95
ch.11.2136200F	11	PDGFD	1.408E-44	2.95	27.80	9.41	0.77	0.60	0.94
cg02502145	2	SOS1	2.086E-47	2.22	42.40	19.07	0.77	0.60	0.94
cg02512074	2	TMEM131	7.803E-11	2.03	18.77	9.23	0.77	0.60	0.94
cg05839875	5	FYB	7.03E-47	2.66	35.96	13.51	0.77	0.60	0.94
cg07338836	7	SEPT7;	1.176E-43	3.53	23.21	6.58	0.77	0.60	0.94
cg08106706	1	ELAVL4	1.063E-43	2.10	31.92	15.20	0.77	0.60	0.94
cg09899173	8	MIR596	0.0142739	3.03	3.56	1.18	0.77	0.60	0.94
cg10879207	12	IRAK3	3.147E-42	2.65	22.11	8.35	0.77	0.60	0.94
cg12708807	21	CRYZL1	1.902E-43	2.43	27.57	11.34	0.77	0.60	0.94
cg22478261	20	PARD6B	3.777E-09	2.01	16.58	8.25	0.77	0.60	0.94
ch.19.1427912R	19	ZNF180	2.625E-42	2.28	24.82	10.89	0.77	0.60	0.94
ch.5.1450929R	5	PDE8B	1.982E-13	2.03	22.51	11.08	0.77	0.60	0.94
cg06098530	10	MYST4	7.147E-44	2.95	25.81	8.76	0.77	0.60	0.94
ch.1.4283968F	1	EPRS	7.409E-43	2.37	26.02	10.97	0.77	0.60	0.94
cg11198604	8	WHSC1L1	1.244E-43	2.30	29.30	12.71	0.77	0.59	0.94
cg12409601	1	PLK3	0.0222773	0.49	2.20	4.47	0.77	0.59	0.94
cg19413693	1	VAV3	1.455E-17	2.29	23.25	10.13	0.77	0.59	0.94
cg20316440	1	RERE	8.759E-18	2.07	27.52	13.29	0.77	0.59	0.94
cg27134342	14	RNASE4	3.385E-16	2.39	20.67	8.63	0.77	0.59	0.94
cg27147615	13	LOC646982	9.672E-14	2.11	21.54	10.20	0.77	0.59	0.94
ch.17.1348129R	17	MBTD1	2.585E-45	2.52	32.71	12.98	0.77	0.59	0.94
ch.18.672159R	18	FHOD3	8.904E-44	2.81	26.20	9.34	0.77	0.59	0.94
ch.5.1829349R	5	CHD1	3.495E-48	2.69	39.10	14.51	0.77	0.59	0.94
cg07218600	13	FGF14	5.283E-42	2.37	22.97	9.67	0.76	0.59	0.94
cg07421341	6	COL19A1	5.874E-16	2.20	22.81	10.36	0.76	0.59	0.94
ch.9.1675990R	9	IKBKAP	3.964E-15	2.20	21.84	9.91	0.76	0.59	0.94
cg00575005	6	RIPPLY2	0.0002302	2.28	7.22	3.17	0.76	0.58	0.94
cg05638359	1	FAM5C	7.352E-43	4.02	20.05	4.99	0.76	0.58	0.94
cg10087172	12	ARHGDIB	2.914E-09	2.32	13.48	5.81	0.76	0.58	0.94
cg23658874	6	EHMT2	2.291E-08	2.26	12.75	5.63	0.76	0.58	0.94
ch.17.620779R	17	ALDH3A2	6.211E-44	2.60	27.87	10.70	0.76	0.58	0.94
ch.2.4366798R	2	USP37	5.551E-42	2.59	21.56	8.31	0.76	0.58	0.94
ch.2.4702176R	2	ATG16L1	8.578E-44	2.84	26.08	9.18	0.76	0.58	0.94
ch.6.609614F	6	ACOT13	3.513E-42	2.45	23.12	9.45	0.76	0.58	0.93
cg04106641	2	ORC4L	6.963E-10	2.03	17.37	8.55	0.76	0.58	0.93
cg06824394	7	C7orf23	5.522E-43	2.67	24.49	9.18	0.76	0.58	0.93
cg07009002	22	MB	5.645E-10	2.02	17.76	8.81	0.76	0.58	0.93
cg07517893	19	PPP1R13L	2.538E-08	2.26	12.69	5.61	0.76	0.58	0.93
cg07641807	13	MIR19A;MIR17HG; MIR20A;MIR17; MIR19B1;MIR18A	7.184E-43	2.12	28.50	13.42	0.76	0.58	0.93

cg10504751	16	GNAO1	0.0027418	2.16	5.99	2.77	0.76	0.58	0.93
cg12158136	12	GUCY2C	1.571E-16	2.25	22.82	10.14	0.76	0.58	0.93
cg14481222	11	BIRC3	4.191E-10	2.05	17.41	8.49	0.76	0.58	0.93
cg14541915	11	PACS1	4.378E-07	2.64	9.26	3.50	0.76	0.58	0.93
cg14992181	4	SCFD2	1.911E-08	3.47	8.61	2.48	0.76	0.58	0.93
cg19499452	14	PACS2	1.412E-07	2.46	10.55	4.28	0.76	0.58	0.93
ch.3.2385974R	3	PARP9	5.43E-44	3.61	23.90	6.62	0.76	0.58	0.93
ch.5.2638848F	5	ARAP3	3.05E-12	2.23	18.05	8.10	0.76	0.58	0.93
cg02685990	4	FAM198B	4.993E-14	2.11	22.00	10.45	0.75	0.57	0.93
cg06818532	3	BBX;BBX	1.425E-10	2.48	13.86	5.58	0.75	0.57	0.93
cg21829783	1	SLAMF6	8.017E-16	2.17	23.16	10.67	0.75	0.57	0.93
ch.2.3405933F	2	B3GALT1	1.487E-42	2.48	24.18	9.74	0.75	0.57	0.93
cg00041401	1	PTPN22	1.053E-07	2.21	12.31	5.58	0.75	0.57	0.93
cg00395420	5	PCDHGA1	1.437E-25	0.49	19.12	39.07	0.75	0.57	0.93
cg07589355	12	RBMS2	3.065E-08	2.06	14.47	7.02	0.75	0.57	0.93
cg09670530	3	MRPL47;NDUFB5	2.216E-08	2.13	13.95	6.55	0.75	0.57	0.93
cg13782615	11	STIM1	5.99E-17	2.32	22.37	9.66	0.75	0.57	0.93
cg15545772	1	TDRD5	4.918E-05	2.17	8.69	4.00	0.75	0.57	0.93
cg18032891	8	C8orf48	3.105E-14	2.28	19.82	8.69	0.75	0.57	0.93
cg19360009	2	YPEL5	4.607E-43	2.42	26.37	10.91	0.75	0.57	0.93
cg21004325	7	ZNF277;DOCK4	1.208E-05	2.66	7.63	2.87	0.75	0.57	0.93
cg23239612	5	CDC23	2.684E-07	2.26	11.36	5.03	0.75	0.57	0.93
cg26869362	11	KBTBD3;AASDHPPT	3.555E-13	2.22	19.27	8.67	0.75	0.57	0.93
cg27482605	19	C19orf51	2.703E-05	2.00	10.25	5.12	0.75	0.57	0.93
ch.1.543953R	1	DDI2	4.413E-10	2.01	18.05	9.00	0.75	0.57	0.93
ch.10.509007F	10	CACNB2	2.979E-42	2.97	20.82	7.01	0.75	0.57	0.93
ch.15.492843F	15	TP53BP1	3.469E-16	2.51	19.46	7.75	0.75	0.57	0.93
ch.16.1684049R	16	GLG1	2.266E-10	2.02	18.36	9.11	0.75	0.57	0.93
cg26453169	5	GAPT	4.041E-13	2.07	21.41	10.35	0.75	0.57	0.93
ch.13.1117341F	13	LMO7	1.753E-17	2.50	21.18	8.49	0.75	0.57	0.93
