# Supplementary material for: Newborn blood DNA epigenetic variations and signaling pathway genes associated with Tetralogy of Fallot (TOF)
Source: PLoS One. 2018 Sep 13;13(9):e0203893. doi: 10.1371/journal.pone.0203893 (PMC6136787; doi:10.1371/journal.pone.0203893)
Supplement: S2 Table — ENCODE data showing the H3K27Ac layering on each CpG site presenting an open chromatin conformation. These CpG targets were also occupied with various transcription initiation factors, mostly PolR2A. The position of each CpG site was also noted in respect to the gene in which it resided. Some of the CpG sites that were differentially methylated resided in intronic or 1st exonic regions, signifying their essential function in modulating transcription. (PDF) [file pone.0203893.s005.pdf]

Target ID	position	correlation coefficient	p value	q value	exp mean	meth mean	H3k27Ac	transcription factors	location
cg27120934	129480619						no	polr2a	intronic
cg02645710	85430085	-0.1881684	0.01877	0.005184	3.3067569	0.267062	no	polr2a,sin3a,yy1	promoter
cg25697769	39097335						high	polr2a,atf2,phf8	promoter
cg04868078	197898229						moderate	gabpa,elf1	exoniccoding
cg23680282	85430336	-0.2844	0.02063	0.002832	0.8488	0.117552	high	polr2a,sin3a,yy1	first intron
cg24132989	88050339						no	none	intronic
cg21364560	95242555	-0.2940194	0.01687	0.002729	10.6345684	0.21759	no	polr2a,e2h2,ctcf,egr1	promoter
cg08821669	120875978	-0.1948	0.000799	0.0003744	11.521848	0.021354	high	polr,ctcf	1stexoncoding
cg14905634	73057369						no	none	1stexonicnoncoding
cg17030055	24645065	-0.1965188	0.01375	0.003963	5.1615058	0.069254	no	polr2a	1stexonicnoncoding
cg11641791	38821429	-0.3949692	0.001122	0.0003591	4.4024256	0.135569	no	no	promoter
cg17728974	105403577						high	polr2a,sin3a	promoter
cg11792281	26443366						no	none	intronic
cg12414181	75287860						no	polr2a,mx11,znf143	promoter
cg02062326	75643727	-0.3113	5.606E-08	1.263E-08	12.6038617	0.06726	high	polr2a,max,gabpa	promoter
cg23274377	220263237	-0.3465128	0.00858	0.0024	9.3818711	0.038806	high	polr,ctcf	promoter
cg18803079	64014643	-0.2201	7.707E-07	5.486E-08	6.7982044	0.74987	no	none	promoter
cg13114458	75905515	-0.3169	7.249E-11	1.306E-11	9.6442085	0.04388	high	polr2a,gabpa,e2f1	promoter
cg01311718	214015137	-0.1869449	0.00000945	0.000000594	7.7274229	0.04322	high	polr2a,znf263	promoter
cg02609279	182325952	-0.4569764	0	0	8.189539	0.692826	high	polr2a,gata1	intronic
cg02071276	224303989	-0.2753	5.283E-10	5.051E-11	10.0044862	0.068616	no	ctcf,Rad21	intronic
cg18295068	166060400	-0.4428	5.466E-07	1.759E-07	6.2330279	0.101475	no	ctcf,Rad21,smc3	1stexonicnoncoding
cg15946310	159436034	-0.483	6.689E-07	5.514E-07	9.945	0.018556	high	polr2a,jun,gabpa,elf1	promoter
cg09365677	240061611	-0.2185	0.000001373	1.464E-07	4.808942	0.035882	no	ctcf,Rad21,smc3	1stexonicnoncoding
ch.11.319992F	319992						high	none	exonic
cg12092090	13617584	-0.2332	0.0001631	0.00001583	6.380564	0.062255	no	none	promoter
cg19533977	57719682	-0.2762	6.976E-08	8.195E-09	13.3620395	0.150087	no	runx3	intronic
cg01400516	47175936	-0.4589059	0.0003817	0.0001314	8.5733169	0.019579	moderate	polr2a,ctcf,gata2	1stintron
cg20101489	224467074	-0.1794531	0.000003681	2.008E-07	5.077037	0.088753	moderate	polr2aq,ctcf,sp2	1stexonicnoncoding
cg23134869	77593233	-0.4681	0	0	5.5618344	0.25591	high	polr2a,yy1	promoter
cg25477497	87228808	-0.6991858	0	0	9.461778	0.367254	no	foxa1,foxa2	exonic
cg25947619	86233220						moderate	polr2a,spi1	intronic
cg14534336	65028980						high	polr2a,stat3,taf1	1stexonicnoncoding
cg07002382	44116782						high	polr2a,cebpb,taf1	1stexonicnoncoding

cg08264335	44116782	-0.3934405	0.002639	0.0009381	9.6083289	0.052874	moderate	none	exonic
cg12273284	12490844	-0.4832	3.467E-08	1.327E-08	7.6487782	0.126881	no	none	intronic
cg26345971	85430215	-0.3811535	5.775E-10	8.988E-11	1.9994466	0.268673	no	polr2a,sin3a	intronic
cg10225640	121790611						high	polr2a,taf1	promoter
ch.1.3587792F	3587792						no	none	intronic
cg00719685	220263698						moderate	polr,ctcf	promoter
cg03814610	89744940						high	polr2a,znf263	promoter
cg17616217	89744940	-0.3216	0.00001986	0.000005823	9.5973308	0.1142	no	none	intergenic
cg19579903	30583082						high	polr2a,yy1,taf1	intronic
cg18469624	53459998	-0.1918	0.00003889	0.000003701	9.505816	0.11861	high	polr2a,etf1,max	promoter
cg10558887	36919409	-0.5807849	0	0	9.162	0.139128	high	none	1stintronic
cg16951385	65029115	-0.1848534	0.01334	0.003867	10.1485358	0.058096	high	polr2a,stat3,taf1	promoter
cg23404012	71066865	-0.2523	1.261E-09	1.281E-10	8.09469	0.046598	high	none	intronic
cg03547245	55335015	-0.3859	1.371E-14	4.337E-15	6.716429	0.08912	moderate	max,ctcf	intronic
cg12129209	102276033	-0.4256	0	0	11.248696	0.059091	high	polr2a,tfx1	promoter
cg03846926	21807211						moderate	taf1,cebpb	1stexonicnoncoding
cg26800788	63649371						no	none	promoter5
cg04254487	134304883	-0.5187	2.031E-09	9.363E-10	8.3824493	0.212056	moderate	ctcf,tebpb	intronic
cg08757862	38807382	-0.3392271	1.85E-19	2.194E-20	7.227838	0.612254	moderate	polr2a,ctcf,ikzf1	promoter
cg10944144	64777807	-0.2851448	1.317E-13	1.141E-14	2.971848	0.021102	high	polr2a,myc,atf2	promoter
cg02981003	134965914						no	none	promoter
cg26401673	43431343	-0.3162	2.53E-13	3.317E-14	8.9418901	0.846658	high	polr2a,gata2,fos	intronic
ch.2.800013F	800013						no	none	intronic
cg19781109	91244526						no	none	intronic
cg06237608	91244584						no	none	promoter5
cg25510609	86233236						high	polr2a,spi1,max	intronic
cg02558537	107328586	-0.4164131	0	0	8.177458	0.027635	high	polr2a,yy1,e2f1	promoter
cg11378242	244999800	-0.2903	1.73E-16	1.529E-17	10.08	0.017718	high	polr2a	1stintronic
cg17485454	87,281,281						no	polr2a,yy1,spi1	1stexonicnoncoding
cg04838988	70984437						no	e2h2,rbbp5	promoter
cg27509202	35484972						high	polr2a,jun,fos	1stexonicnoncoding
cg19021985	22398190	-0.2973	5.944E-09	7.885E-10	7.7477895	0.779761	moderate	polr2a,SPI1	1stexonicnoncoding
cg00994804	36259383						high	polr2a,rbb5	exoniccoding
ch.1.659794R	659794						moderate	none	promoter
cg22664298	29680347						no	none	intergenic
