# Supplementary material for: Newborn blood DNA epigenetic variations and signaling pathway genes associated with Tetralogy of Fallot (TOF)
Source: PLoS One. 2018 Sep 13;13(9):e0203893. doi: 10.1371/journal.pone.0203893 (PMC6136787; doi:10.1371/journal.pone.0203893)

	Target ID	position	correlation coefficient	p value	q value	exp mean	meth mean
1	cg02645710	85430085	-0.188168	0.01877	0.005184	3.3067569	0.267062
2	cg23680282	85430336	-0.2844	0.02063	0.002832	0.8488	0.117552
3	cg21364560	95242555	-0.294019	0.01687	0.002729	10.634568	0.21759
4	cg08821669	1.21E+08	-0.1948	0.000799	0.0003744	11.521848	0.021354
5	cg17030055	24645065	-0.196519	0.01375	0.003963	5.1615058	0.069254
6	cg11641791	38821429	-0.394969	0.001122	0.0003591	4.4024256	0.135569
7	cg02062326	75643727	-0.3113	5.606E-08	1.263E-08	12.603862	0.06726
8	cg23274377	2.2E+08	-0.346513	0.00858	0.0024	9.3818711	0.038806
9	cg18803079	64014643	-0.2201	7.707E-07	5.486E-08	6.7982044	0.74987
10	cg13114458	75905515	-0.3169	7.249E-11	1.306E-11	9.6442085	0.04388
11	cg01311718	2.14E+08	-0.186945	0.00000945	5.94E-07	7.7274229	0.04322
12	cg02609279	1.82E+08	-0.456976	0	0	8.189539	0.692826
13	cg02071276	2.24E+08	-0.2753	5.283E-10	5.051E-11	10.004486	0.068616
14	cg18295068	1.66E+08	-0.4428	5.466E-07	1.759E-07	6.2330279	0.101475
15	cg15946310	1.59E+08	-0.483	6.689E-07	5.514E-07	9.945	0.018556
16	cg09365677	2.4E+08	-0.2185	1.373E-06	1.464E-07	4.808942	0.035882
17	cg12092090	13617584	-0.2332	0.0001631	0.00001583	6.380564	0.062255
18	cg19533977	57719682	-0.2762	6.976E-08	8.195E-09	13.36204	0.150087
19	cg01400516	47175936	-0.458906	0.0003817	0.0001314	8.5733169	0.019579
20	cg20101489	2.24E+08	-0.179453	3.681E-06	2.008E-07	5.077037	0.088753
21	cg23134869	77593233	-0.4681	0	0	5.5618344	0.25591
22	cg25477497	87228808	-0.699186	0	0	9.461778	0.367254
23	cg08264335	44116782	-0.393441	0.002639	0.0009381	9.6083289	0.052874
24	cg12273284	12490844	-0.4832	3.467E-08	1.327E-08	7.6487782	0.126881
25	cg26345971	85430215	-0.381154	5.775E-10	8.988E-11	1.9994466	0.268673
26	cg17616217	89744940	-0.3216	0.00001986	5.823E-06	9.5973308	0.1142
27	cg18469624	53459998	-0.1918	0.00003889	3.701E-06	9.505816	0.11861
28	cg10558887	36919409	-0.580785	0	0	9.162	0.139128
29	cg16951385	65029115	-0.184853	0.01334	0.003867	10.148536	0.058096
30	cg23404012	71066865	-0.2523	1.261E-09	1.281E-10	8.09469	0.046598
31	cg03547245	55335015	-0.3859	1.371E-14	4.337E-15	6.716429	0.08912
32	cg12129209	1.02E+08	-0.4256	0	0	11.248696	0.059091
33	cg04254487	1.34E+08	-0.5187	2.031E-09	9.363E-10	8.3824493	0.212056
34	cg08757862	38807382	-0.339227	1.85E-19	2.194E-20	7.227838	0.612254
35	cg10944144	64777807	-0.285145	1.317E-13	1.141E-14	2.971848	0.021102
36	cg26401673	43431343	-0.3162	2.53E-13	3.317E-14	8.9418901	0.846658
37	cg02558537	1.07E+08	-0.416413	0	0	8.177458	0.027635
38	cg11378242	2.45E+08	-0.2903	1.73E-16	1.529E-17	10.08	0.017718
39	cg19021985	22398190	-0.2973	5.944E-09	7.885E-10	7.7477895	0.779761

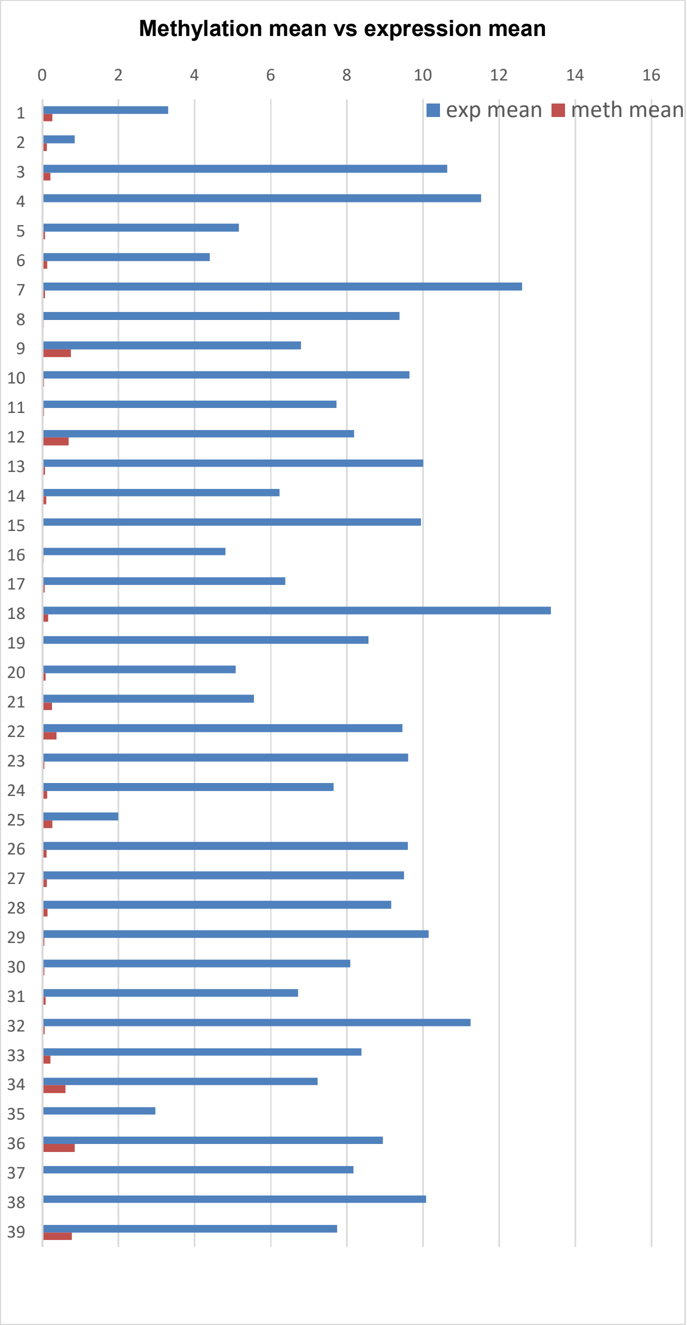

Supplement: S3 Table — 39 differentially methylated CpG targets were correlated with expression (RNA-seq) data. A bar chart was generated for each CpG target showing the proportion of methylation and mean of expression of the gene in which the CpG target resided. (PDF) [file pone.0203893.s006.pdf]
