# Supplementary material for: Newborn blood DNA epigenetic variations and signaling pathway genes associated with Tetralogy of Fallot (TOF)
Source: PLoS One. 2018 Sep 13;13(9):e0203893. doi: 10.1371/journal.pone.0203893 (PMC6136787; doi:10.1371/journal.pone.0203893)
Supplement: S4 Table — (PDF) [file pone.0203893.s007.pdf]

Target ID	Gene	CHR	% Methylation		Fold Change	FDR	AUC	CI_	CI_
			Cases	Controls		p-Val		upper	lower
ch.1.659794R	UBR4	1	13.09	6.08	2.15	2.15E-16	0.76	0.9	0.63
cg02609279	ITGA4	2	16.61	8.09	2.05	1.56E-39	0.92	1.0	0.83
ch.2.800013F	BIRC6	2	11.69	5.53	2.12	6.69E-14	0.80	0.9	0.67
cg26401673	ANO10	3	16.56	7.78	2.13	1.22E-39	0.80	0.9	0.67
cg08757862	TLR1	4	16.97	7.39	2.3	5.51E-40	0.82	0.9	0.69
cg17485454	MAPK10	4	16.60	8.24	2.01	1.84E-39	0.78	0.9	0.65
cg22664298	ADAMTS19	5	18.76	9.24	2.03	5.81E-40	0.75	0.9	0.61
cg15946310	TTC1	5	16.42	6.48	2.53	3.82E-40	0.91	1.0	0.83
cg26800788	PDE4D	5	18.92	8.92	2.12	3.6E-40	0.82	0.9	0.69
cg27120934	LAMA2	6	8.07	19.08	0.42	9.04E-26	0.98	1.0	0.95
cg04254487	TBPL1	6	23.75	11.35	2.09	3.01E-41	0.82	0.9	0.69
cg19021985	PPP3CC	8	36.66	18.23	2.01	3.05E-44	0.76	0.9	0.62
cg02981003	GPR123	10	15.51	7.72	2.01	3.19E-39	0.81	0.9	0.69
cg18469624	PRKG1	10	19.24	9.52	2.02	4.75E-40	0.84	1.0	0.73
cg12273284	CAMK1D	10	13.19	4.99	2.65	2.12E-39	0.87	1.0	0.77
cg27509202	CREM	10	9.96	4.96	2.01	3.24E-10	0.76	0.9	0.62
cg14534336	JMJD1C	10	9.75	4.78	2.04	2.7E-10	0.88	1.0	0.78
cg02558537	CWF19L2	11	10.03	4.97	2.02	2.08E-10	0.78	0.9	0.64
cg23680282	LRRIQ1	12	17.31	6.17	2.81	1.12E-40	0.97	1.0	0.92
cg14905634	TRHDE	12	15.37	32.00	0.48	3.66E-31	0.95	1.0	0.88
cg12129209	PPP2R5C	14	12.68	6.00	2.11	2.42E-15	0.83	1.0	0.71
cg25947619	AKAP13	15	18.92	8.64	2.19	2.72E-40	0.88	1.0	0.78
cg03547245	MSI2	17	10.26	4.40	2.33	5.14E-14	0.83	1.0	0.71
cg11792281	NLK	17	11.65	23.41	0.50	1.05E-23	0.94	1.0	0.87
cg00994804	RUNX1	21	13.65	6.17	2.21	4.28E-39	0.76	0.9	0.62
